# Nutrition interventions at point-of-sale to encourage healthier food purchasing: a systematic review

**DOI:** 10.1186/1471-2458-14-919

**Published:** 2014-09-05

**Authors:** Selma C Liberato, Ross Bailie, Julie Brimblecombe

**Affiliations:** Menzies School of Health Research, Charles Darwin University, John Mathews Building, Casuarina, NT Australia; School of Population Health, Division of Health Sciences, University of South Australia, P4-27 F Playford Building, City East Campus, Adelaide, SA Australia

## Abstract

**Background:**

Point-of-sale is a potentially important opportunity to promote healthy eating through nutrition education and environment modification. The aim of this review was to describe and review the evidence of effectiveness of various types of interventions that have been used at point-of-sale to encourage purchase and/or eating of healthier food and to improve health outcomes, and the extent to which effectiveness was related to intensity, duration and intervention setting.

**Methods:**

Records from searches in databases were screened and assessed against inclusion criteria. Included studies had risk of bias assessed. Intervention effectiveness was assessed for two outcomes: i) purchase and/or intake of healthier food options and/or nutrient intake; and ii) mediating factors that might effect the primary outcome.

**Results:**

The search identified 5635 references. Thirty-two papers met the inclusion criteria. Twelve studies had low risk of bias and were classified as strong, nine were moderate and 11 were weak. Six intervention types and a range of different outcome measures were described in these papers: i) nutrition education and promotion alone through supermarkets/stores; ii) nutrition education plus enhanced availability of healthy food; iii) monetary incentive alone; iv) nutrition education plus monetary incentives; v) nutrition intervention through vending machines; and vi) nutrition intervention through shopping online. The evidence of this review indicates that monetary incentives offered to customers for a short-term look promising in increasing purchase of healthier food options when the intervention is applied by itself in stores or supermarkets. There was a lack of good quality studies addressing all other types of relevant point-of-sale interventions examining change in purchase and/or intake of healthier food options. There were few studies that examined mediating factors that might mediate the effect on the primary outcomes of relevant interventions.

**Conclusions:**

A range of intervention types have been used at point-of-sale to encourage healthy purchasing and/or intake of healthier food options and to improve health outcomes. There is a need for more well designed studies on the effectiveness of a range of point-of-sale interventions to encourage healthier eating and improve health outcomes, and of the mediating factors that might impact these interventions.

**Electronic supplementary material:**

The online version of this article (doi:10.1186/1471-2458-14-919) contains supplementary material, which is available to authorized users.

## Background

**Figure 1:**
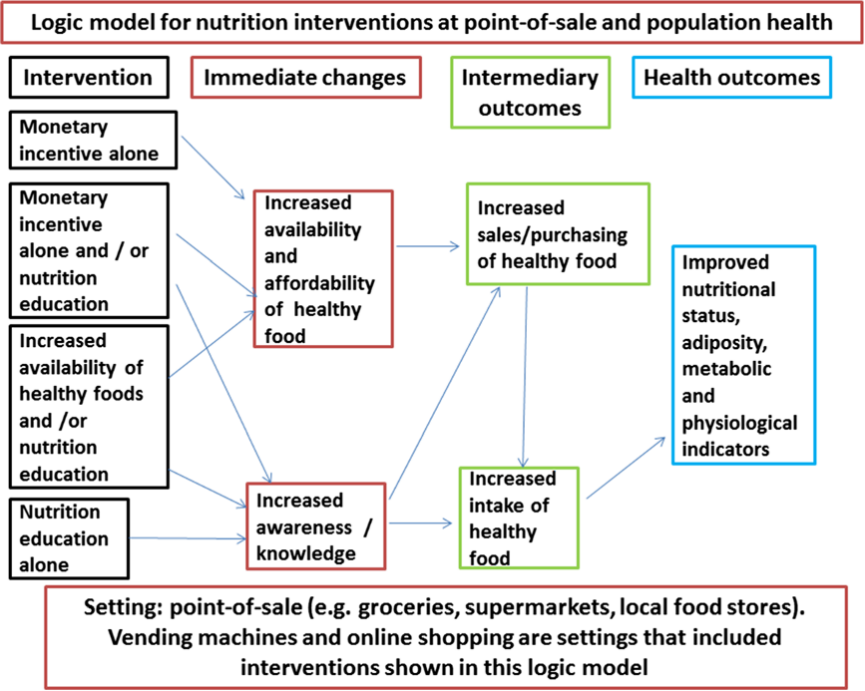
**Logic model for nutrition interventions and outcomes.**

Unhealthy eating contributes to increased prevalence of preventable chronic diseases including obesity, cardiovascular disease, type 2 diabetes and many forms of cancer [1]. These diseases pose a substantial threat to the health of populations. It is difficult to implement and maintain behaviours that promote good health, including healthy eating behaviours and this is made more difficult by an environment where convenient and cheap ready-to-eat foods of low nutritional value are readily available and frequently advertised. Interventions targeting a variety of cultural, physical and social environment factors, as well as those targeting personal factors may be effective in positively influencing healthy food intake [2, 3]. Point-of-sale, defined as the location where selection of food for purchase occurs, is a potentially important opportunity to promote healthy eating through environment modification and nutrition education. For example, interventions designed to increase availability and/or affordability of healthy foods such as fruit, vegetables, lean meats and reduced fat dairy products are expected to produce behaviour change by shaping the environment. This may include providing incentives and rewards to encourage behavior change. Interventions including nutrition education are also expected to lead to behaviour change by increasing levels of awareness and knowledge. This change may lead to intermediary outcomes such as an increase in purchase and/or intake of healthier foods, which in turn may lead to improved health outcomes. A logic model for nutrition interventions at point-of-sale and population health is shown in Figure 1.

In order to design interventions to yield positive behavior change it is important to consider behaviour change theories. Social Cognitive Theory is one such theory that proposes a triadic interaction of environmental and personal factors to explain the process of behavior change [4]. Environmental factors represent the situational influences in which behavior is performed (such as food affordability and availability) while personal factors include instincts, drives, traits, and other individual motivational forces. Some of the mediator factors that shape human learning and behavior change include self-efficacy, outcome expectations, self-control, reinforcements, emotional coping and observational learning [4]. Therefore, according to this theory, it may be important to provide support to raise individual confidence and self-efficacy, as well as environmental support to motivate behavior change, such as the provision of an incentive or reward.

Currently, there are eight systematic reviews of the effectiveness of point-of-sale interventions to support healthier food purchasing. One review [5] identified small-store intervention strategies aimed at increasing healthy food access and consumption. This review included eight trials published in peer-reviewed journals and eight evaluated trials from the grey literature. The quality of the studies was not assessed. Due to the limited number of studies and study variability no conclusive findings could be drawn. A second review examined the effectiveness of tailored nutrition education on objective outcome measures only [6]. There were substantial differences in the theoretical underpinnings of the interventions applied in the four included studies. Two trials provided participants with discount coupons for healthier food choices and the other two trials included goal-setting, making it difficult to draw conclusions on the effectiveness of tailored nutrition education. The third review [7] focused on describing retail grocery store marketing strategies and included 125 articles. This review concluded that access to healthy foods and less access to unhealthy foods may increase healthful eating; product packaging (size) and images affect purchase and consumption; coupons and cross-promotion increase product liking and purchase; healthy checkout aisles can be helpful for reducing unhealthy impulse purchases, and; shelf labels, samples and taste testing, and end-of-aisle displays are most noticed by customers [7]. The fourth review [8] focused on the effect of food price changes on food purchasing patterns in a laboratory-type environment testing hypothetical food decision-making processes. The review found that price changes modify purchases of targeted foods, but that research findings on the overall nutritional quality of purchases are mixed because of product substitution effects. A further finding was that there is mixed support for combining price changes with additional interventions [8]. The fifth review [9] used four categories to evaluate impact of intervention type on promotion of healthful food choices in supermarket and grocery stores: 1) price reduction, 2) increased availability, 3) promotion and advertising strategies and 4) point-of-purchase strategies. There appears to be some overlap and inconsistency in categorising strategies to these four intervention types. This review provides a description of available studies and quality of evidence. The sixth review [10] aimed at assessing effectiveness of monetary subsides in promoting healthier food purchases and consumption and included 24 articles on 20 studies in store. Other point-of-sale settings were not included. All but one study found subsidies on healthier foods to significantly increase the purchase and consumption of promoted products [10]. The seventh review [11] assessed the potential effectiveness of food and beverage taxes and subsidies for improving body weight outcomes. This review included 20 studies and found minimal impacts on weight due to soda taxes. On the other hand higher fast-food prices and lower fruit and vegetables prices were associated with lower weight outcomes. The eighth review [12] aimed to assess the effectiveness of providing food product health information at the point of purchase. This review included 16 articles (reporting on 17 studies) that derived their outcome measure from point-of-sale data. Several studies reported no significant effects of product health information on actual purchase behaviour. Interventions were more likely to be effective when they lasted for a longer time, when they included additional intervention components, and when they targeted the absence of unhealthy nutrients instead of or in addition to the presence of healthy nutrients [12].

None of the above mentioned reviews have examined the effectiveness of the range of interventions used at point-of-sale in supermarkets, grocery stores and vending machines nor considered the influence of intensity and duration on effectiveness. These reviews point to the broad range of intervention type (including length of intervention and intensity of the delivery of the intervention) and the need for increased use of more standardized measures. Even though this difference is particularly important to consider because of the cost involved in longer term/interactive interventions compared to shorter term and less interactive type interventions, as far as we know, no reviews to date have considered these differences.

The aim of this review was to address the evidence gap on the effectiveness of point-of-sale interventions to increase purchase and/or intake of healthier foods and more specifically: i) to describe the various types of interventions that have been used at point-of-sale to encourage purchase and/or intake of healthier foods and improve health outcomes; ii) to review the evidence of effectiveness of different types of interventions and the extent to which effectiveness was related to intensity, duration and intervention setting, and iii) if appropriate to perform a meta-analysis of relevant interventions.

The nutrition interventions included in this review comprise any intervention at point-of-sale aiming to influence any elements of the triadic interaction of behaviour, personal and environmental factors. These include interventions aiming to:

- increase the availability of healthier foods by increasing the store stock of healthy foods.
- increase the affordability of healthier foods by providing monetary incentives (also named as a financial incentives or monetary benefits) including food coupons or food vouchers of any value, offered to customers and/or monetary incentives offered to store-owners to promote increased availability of healthy foods.
- facilitate the adoption of healthier food purchasing and eating behaviour through nutrition education and promotion. This nutrition education and promotion may include interactive activities (intense) where there are interactions with customers and/or store-owners; or non-interactive activities (less-intense) such as use of mass media and promotional or educational flyers where there is no direct customer/store-owner interaction. The nutrition education and promotion may also include feedback to customers based on their product purchases.
- Or any combination of the above.

## Methods

The PubMed, EBSCO and Science Direct database was searched on 10^th^ Mar 2011 using the following search terms “Fast food*” or “convenience store*” or “take away*” or restaurant* or “dining room*” or cafeteria* or café* or diner or “food store*” or “food outlet*” or “corner store*” or supermarket* or grocer* or “vending machine*” or “automatic food dispenser*” or “community store*” in the title field combined with “diet” or “nutrition” or “food*” or “vegetable*” or “fruit*” in the abstract field and with availab* or affordab* or access* or strateg* or promotion* or program* or initiative* or intervention* or practice* or marketing* or activit* or “food quality” in the abstract field and found 3968 references. The search was run again on 16^th^ March 2012 and found 305 references and again on 3^rd^ Jan 2013 and found 563 references. The search was run again on 8^th^ May 2014 and found 796 new articles. There was no limit regarding date of publication specified in the search.

### Inclusion/exclusion criteria

The inclusion criteria used to identify studies is shown in Table 1.

**Table 1:** **Characteristics of the inclusion criteria**

Characteristics	Inclusion criteria requirements
Intervention	Aimed i) to impact availability, affordability and/or ability to choose healthier foods and drinks, or ii) to influence food and drink purchases (including, infrastructure or monetary incentives as well as marketing strategies including promotion and placement strategies).
	Implemented at point-of-sale in a supermarket, grocery store and/or vending machine.
	Clearly described to justify the study being included within the scope of interventions for this review.
Population	Conducted in stores/supermarkets or vending machines aimed at both the general population and/or organizations (e.g., monetary incentive to store to increase availability of healthy food options) but not including studies focusing on people with specific diseases. Age was not specified in the search.
Study design	Randomised controlled trials, controlled before and after studies or interrupted time series designs and analyses.
Outcomes	At least one primary outcome: Primary: nutritional/food intake, food purchasing Secondary: dietary biomarkers, consumer awareness, consumer knowledge, consumer self-efficacy, consumer outcome expectation, consumer beliefs, consumer attitudes, shelf-label use, shelf-label recall, healthier food stocking*, availability*, quality*, store retail practices*, policy, management* and/or organisational practices*, anthropometry, physiological measures, population health data such as mortality and/or morbidity data.
Comparator of interest	Intervention described above compared to no intervention reporting any of the outcomes described above.
Statistical analysis	Effect of the intervention in relation to an historical or concurrent control group for the primary and/or secondary outcome measures.
Language	English, Portuguese or Spanish as one author is fluent is these three languages.

### Screening

The references were screened against the inclusion criteria by two authors based on title and abstract. The findings from the two authors were cross-checked. References identified by at least one author were reassessed by both authors. Where both authors agreed, the references were fully assessed against inclusion criteria. Studies meeting the criteria were included in this review. Any discordance between reviewers for screening was discussed with the third author (RB).

### Quality criteria

All studies were assessed by two authors (SCL and JB) based on the following criteria: selection bias, study design, confounders, blinding, data collection methods, and handling of withdrawals and dropouts using a Quality Assessment Tool for review articles [13]. Studies were rated against each criterion as strong, moderate, or weak. Studies having low risk of bias were rated strong according to the Quality Assessment Tool for Quantitative Studies Dictionary developed for the Effective Public Health Practice Project [14] while those having high risk of bias were rated weak. The ratings against each criterion were used to determine an overall quality rating for each study (no weak ratings, one weak rating and more than one weak rating indicated a strong, moderate and weak overall rating, respectively). Any discrepancies were discussed with the third author (RB).

### Data extraction

Data were extracted into a standardized form by one author (SCL) and checked by a second author (JB). Data extracted from each eligible study included the following variables: study context, study design and quality, participant characteristics, intervention specifics including theoretical framework used to design the intervention and outcome effects.

### Data synthesis and analysis

The studies were synthesized according to the intervention applied and then further categorized according to the intervention duration and intensity. The unit of analysis varied across the studies as some studies analysed at both the store (e.g., differences in low-fat milk supermarket sales) and individual customer (e.g., proportion of high-fat milk drinkers) levels whereas others analysed at the store and/or individual level.

Summary measures included means or difference in means from baseline to follow up.

## Results

### Results of the search

**Figure 2:**
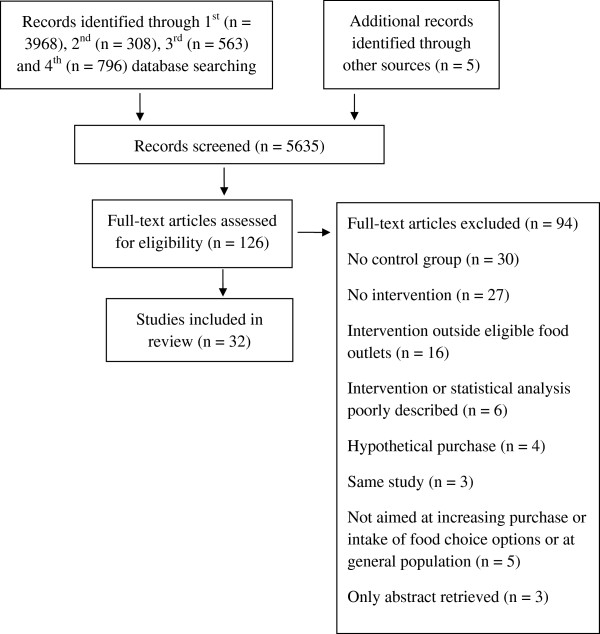
**Flow diagram.**

A total of 5630 references were retrieved from the database searches and five references identified from professional contacts. A total of 5635 references, based on title and abstract, were screened against the criteria by two authors (SCL and JB), and cross-checked, which reduced the number to 126 references. A total of 126 full papers were fully assessed against inclusion criteria (Figure 2).

### Excluded studies

Ninety-four studies were excluded (Additional file 1: Table S1). The main reasons for exclusion were: absence of a control group (30 studies), absence of intervention (27 studies), intervention not applied at eligible food outlets (16 studies), intervention or statistical analysis poorly described (six studies). Other reasons for exclusion included: the intervention was not aimed at improving purchase and/or intake of healthier food options and/ or at the general population; hypothetical purchase; abstract only retrieved; and, the same study reported with no additional relevant findings to that of an included study.

### Risk of bias in included studies

Sixteen studies were randomised controlled trials, 14 were controlled before and after trials (CBA), one was a CBA with a non-equivalent comparison group [15] and one was a quasi-experimental repeated measures study [16]. Twelve studies had low risk of bias and were classified as strong [16–27], nine were moderate [28–36] and 11 were weak [15, 37–46] (Additional file 2: Table S2). The studies classified as weak were still included as they may still have made an important, if lesser, contribution. Most of the studies had high risk of selection bias, low risk due to the allocation process, low risk of confounders, no mention of blinding of assessors awareness of control and intervention participants and of participants awareness of research question, valid data collection methods for primary outcomes, and low attrition bias.

### Included studies

Thirty-two studies were included in this review, 30 studies occurred in urban settings and two in rural settings [23, 24]. Six studies provided information on the population socio-economic status, and eleven, on the participant socio-economic status. Twenty-seven studies were undertaken in stores or supermarkets, four in vending machines located at schools and worksites [17, 20, 30, 41] and one with an online supermarket [34] (Additional file 5: Table S5). Definition on what was meant by a store, or small supermarket and how these might be different to each other and to other types of food outlets was generally not clearly reported. Therefore it was deemed appropriate in this review to use the same term to describe the point-of-sale location as that reported in the original study.

### Types of interventions applied in the included studies

There were substantial differences in the type of interventions applied in the included studies and the type of interventions used in different point-of-sale settings. Because of the wide range of different types of interventions and different mechanisms by which various interventions were expected to work, it was deemed appropriate to categorize the studies by more specific intervention types. The 32 studies were categorized in the following six groups according to intervention type and setting (Additional file 3: Table S3):

1. Nutrition education and promotion alone through supermarkets/stores including posters, signs, flyers, nutrition education sessions, store-tours, taste-testing and cooking demonstrations (15 studies; three short-term non-interactive, three long-term non-interactive and nine short-term interactive).
2. Nutrition education plus enhanced availability of healthy food through increasing the stock of healthy food and/or drinks (one long-term study).
3. Monetary incentive alone (four studies; three short-term and one long-term)
4. Nutrition education plus monetary incentives such as price discount or food coupons or food vouchers (nine studies) with two subcategories:
  Monetary incentives provided to customers (6 studies; 5 short-term and 1 long-term)
  Monetary incentives provided to customers and store-owners (three studies; one short-term and two long-term)
5. Nutrition intervention through vending machines (four short-term studies) with six subcategories:
  Nutrition education alone (two studies)
  Enhanced availability of healthy food options alone (two studies)
  Nutrition education and enhanced availability of healthy food options (two studies)
  Monetary incentive alone (one study)
  Nutrition education and monetary incentive (one study)
  Nutrition education and enhanced availability of healthy food options and price discount (one study)
6. Nutrition intervention through shopping online (one study)

Due to the range in duration and intensity of interventions described in the included studies it was deemed appropriate to categorize the studies according to duration and intensity. Subcategories for intervention duration included long-term if lasting more than six months, and short term if lasting six months or less. Subcategories for intervention intensity included less intense, (referred to as non-interactive), where no interaction with customers occurred such as in the case of hand-outs (flyers, recipes, guidelines), media advertisement, public events, posters, signs and shelf-takers; and more intense, (referred to as interactive), where interaction with customers occurred through activities such as store-tours, cooking demonstrations, taste-testing, or where nutrition education sessions included feedback to customers based on their food and beverage purchases and/or where opportunities for goal-setting were made available to customers at point-of-purchase (Additional file 3: Table S3 and Additional file 4: Table S4).

### Effectiveness of interventions applied in the included studies

The findings are reported for primary outcomes (effect on store sales/purchase/intake) and secondary outcomes (effect on mediator factors) according to the six intervention types described above.

### 1. Nutrition education and promotion alone through supermarkets/stores

Nutrition education alone was the intervention applied in 15 studies (Additional file 3: Table S3). Thirteen studies were undertaken in the USA, one in New Zealand and one in Canada (Additional file 5: Table S5). The studies included from one [15, 21, 45, 46] to 372 [28] supermarkets/stores and from 61 [46] to 2399 participants [40]. Most participants in all studies were female (Additional file 5: Table S5). The target products ranged from a single product such as low fat milk] [23, 24, 39] to fruits and vegetables [28, 31] and multiple food groups [15, 16, 19, 21, 22, 25, 32, 40, 45, 46] (Additional file 4: Table S4). The remaining eight studies targeted specific nutrients such as fibre (four studies), fat (four studies), calories (two studies), sodium (two studies), cholesterol (one study) and calcium (one study) and usually promoted foods from multiple food groups. The four studies that reported the criteria used to identify healthy food options used an established criteria [21, 22] or defined their own criteria [25, 32] (Additional file 4: Table S4).

The theoretical framework used to inform the nutrition education was reported in four studies with the Social Cognitive Theory used in two of these studies (Additional file 4: Table S4). The shortest intervention was a supermarket tour lasting two hours [15] and the longest intervention lasted two years [17, 26] and included media advertisement, shelf-labels, guidelines for assisting healthier product choice, and a list of target products and dietary hints available at the counter (Additional file 3: Table S3).

*Short-term non-interactive interventions*

Three studies [19, 28, 30] measured intervention effect on point-of-sale data (Table 2 and Additional file 4: Table S4). There were insufficient studies applying short-term non-interactive interventions to draw clear conclusions on their impact on purchase and/or intake of healthier food choices or their impact on factors that might effect the primary outcome (Table 2).

**Table 2:** **Primary and secondary outcomes of studies applying short-term non-interactive interventions classified as nutrition education and promotion alone**

References	Primary outcomes	Secondary outcomes
Achabal (moderate)^1^ [28]	No impact on sales data for any of the six products.	Perceived quality image of the produce department was improved.
Booth (weak) [28]	Compared to baseline there was an increase in self-reported purchase of the healthier milk option.	Compared to baseline there was an increase in intention, attitude and beliefs in the intervention group.
Jeffery (strong) [19]	Trends in sales data were likely to be unrelated to the intervention.	Knowledge increased in both intervention and control stores.

^1^Classification of the study regarding risk of bias between brackets.

*Long-term non-interactive interventions*

Three studies [16, 25, 40] applied long-term interventions including non-interactive nutrition education activities (Table 3). There were insufficient studies applying long-term non-interactive interventions to draw clear conclusions on their impact on purchase and/or intake of healthier food choices or their impact on factors that might effect the primary outcome (Table 3).

**Table 3:** **Primary and secondary outcomes of studies applying long-term non-interactive interventions classified as nutrition education and promotion alone**

References	Primary outcomes	Secondary outcomes
Ernst (weak)^1^ [40]	No difference between intervention and comparison in volume sold of target product. There was an increase in the target milk product as a percentage of total milk sales in the intervention group.	Customer knowledge increased.
Levy (strong) [16]	Positive program effect. Product sector market share increased in the intervention group compared to the same product sector market in the control study.	
Rodgers (strong) [25]	No intervention effect for self-reported purchase of healthier foods. Out of eight food categories assessed one food category showed a modest increase in percentage sales of recommended fresh produce to all fresh produce in the intervention stores.	No intervention effect for change in food preparation and knowledge.

^1^Classification of the study regarding risk of bias between brackets.

*Short-term interactive interventions*

Nine studies [15, 21–24, 31, 32, 45, 46] applied short-term interventions including interactive nutrition education activities (Table 4). The activities were delivered weekly in two studies [45, 46], monthly in one study [32] and tailored at each purchase in another study [22] (Additional file 3: Table S3). Frequency of activity delivery was not reported for the other five studies [15, 21, 23, 24, 31]. Six studies [21, 23, 24, 45, 46] collected point-of-sale data (Table 4 and Additional file 5: Table S5). There were insufficient studies applying short-term interactive interventions to draw clear conclusions on their impact on purchase and/or intake of healthier food options or on mediating factors that might effect the primary outcome (Table 4).

**Table 4:** **Primary and secondary outcomes of studies applying short-term interactive interventions classified as nutrition education and promotion alone**

References	Primary outcomes	Secondary outcomes
Connell (moderate)^1^ [31]	No difference in self-reported intake.	No difference in intention to increase intake of healthier food options. Knowledge increased but there was no difference between intervention and control communities in relation to attitude and beliefs.
Foster (moderate) [32]	Intervention group purchased more skim 1% fat milk, water and 2 of 3 types of frozen meals compared to the control group. There were no differences between groups for cereal, whole or 2% fat milk, beverages, or diet beverages.	
Milliron (strong) [21]	Intervention group purchased more fruit & dark-green vegetables but there were no differences in the total fat, saturated fat or vegetable serves purchased compared to the control group.	Awareness of shelf-talkers was higher in the intervention than in the control stores.
Ni Mhurchi (strong) [22]	There was no difference in the purchase of target foods or target nutrients between the intervention and control groups.	
Reger 1999 (strong)^1^ [23]	Purchase of healthier milk options increased from baseline to the end of the intervention and unhealthier options decreased compared to the control communities. This difference remained 6 months later.	
Reger 2000 (strong) [25]	No difference in sales of healthier milk options as a proportion of overall milk sales between intervention and comparison communities at the end of the intervention and at 6 months follow up.	
Silzer (weak) [15]	Intervention group reported more purchasing healthier food options.	Intervention group reported more reading of labels and preparation of healthier food options.
Winett, 1991 (weak) [45]	Purchase of healthier food options in two of the 13 categories increased in the intervention group compared to the control group.	
Winett, 1991 (weak) [46]	Purchase of healthier food options in two categories increased in the intervention group compared to the control group.	

^1^Classification of the study regarding risk of bias between brackets.

There were no studies applying long-term interactive interventions.

In summary, there is large heterogeneity in the outcome measures of the studies applying a short-term or long-term interactive or non-interactive intervention to draw clear conclusions on their impact on purchase and/or intake of healthier food options, or on factors that might mediate the effect of these interventions.

### 2. Nutrition education plus enhanced availability of healthy food

Only one study classified as strong [18] was included in this category (Table 5). It was conducted in food stores in low-income multiethnic communities in the USA targeting both children and their adult caregivers. The majority of the caregivers were female (Additional file 5: Table S5). The long-term nutrition education intervention consisted of four 2-month themed phases over eight months and aimed to increase the stock of healthy food options from multiple food groups. The criterion used to identify healthier food options was not reported (Additional file 4: Table S4). It was based on the Social Cognitive Theory and the Theory of Planned Behavior Change and included interactive and non-interactive nutrition education activities (Additional file 4: Table S4). Due to this being the only study in this category, no conclusion on the impact of nutrition education plus enhanced availability on purchase and/or intake of healthier food options or on mediating factors that might effect the primary outcome (Table 5) could be made.

**Table 5:** **Primary and secondary outcomes of the one study applying a long-term interactive intervention classified as nutrition education plus enhanced availability of healthy food**

Reference	Primary outcomes	Secondary outcomes
Gittelsohn 2010a (strong)^1^ [18]	No effect on healthier food intake was observed. Healthy eating index scores were higher for some food categories in the intervention group compared to the control group.	The intervention had a positive effect in improving some mediator factors (caregiver knowledge and awareness of healthier food options) but had no effect on others (self-efficacy, intention and health belief).

^1^Classification of the study regarding risk of bias between brackets.

### 3. Monetary incentives alone

Four studies were included in this category. Monetary incentives were applied for a short-term in three studies and included price discounts of 12.5% [22] and 50% [27] on selected food and drink products, or store coupon/vouchers valued at $10 per week [33]. A monetary incentive including a cash-back rebate of up to 25% for healthy food purchases was applied for a long-term in one study [26]. One study was conducted in the USA [33], one in South Africa [26] and one in The Netherlands [27] and the other in New Zealand [22] (Additional file 5: Table S5). One study was conducted in three sites of a city [33], one in four supermarkets [27], one in eight supermarkets in three cities [22] and one in over 400 supermarkets across all provinces in one country [26] (Additional file 5: Table S5). The number of study participants ranged from 173 [27] to 67,343 [26] and were mostly female (Additional file 5: Table S5). Fruits and vegetables were the target food groups in two studies [27, 33] (Additional file 4: Table S4). One study [22] targeted 1032 food products from multiple food groups and used the Heart Foundation’s Tick program criterion to identify healthier food options. The remaining study [26] in this category targeted more than 6000 items and provided no further information on the criteria used to identify the healthier food options (Additional file 4: Table S4).

Monetary incentive alone including short-term interventions seems to be effective in increasing purchase and/or intake of healthier food options when a relevant monetary incentive is offered to customers. There were insufficient number of studies to draw clear conclusions on long-term studies or on the mediating factors that might effect the primary outcome (Table 6).

**Table 6:** **Primary and secondary outcomes of studies applying short- and long- term interventions classified as monetary incentive alone**

References	Primary outcomes	Secondary outcomes
Herman (moderate)^1^ [33]	Intake in serves of healthy foods increased more at the intervention than at the control sites and the effect was sustained six months after the intervention.	
Ni Mhurchi (strong) [22]	There was no difference in sales of saturated fat between intervention and control groups but purchase of healthier discounted foods was higher in the intervention group than in the control group and these effects were sustained 12 months after the intervention.	
Waterlander (strong) [27]	Intake of fruit and vegetable increased in the intervention group compared to the control group.	
Sturm^2^ (strong) [26]	Participation in a rebate program for healthy foods led to increases in purchases of healthy foods and to decreases in purchases of less-desirable foods.	

^1^Classification of the study regarding risk of bias between brackets.

^2^Long-term intervention.

### 4. Nutrition education plus monetary incentives

Nine studies were included in this category (Additional file 3: Table S3).

**Table 7:** **Primary and secondary outcomes of studies applying short- and long- term interactive interventions classified as nutrition education plus monetary incentives aimed at customers**

References	Primary outcomes	Secondary outcomes
Ni Mhurchi (strong)^1^ [22]	There was no difference in sales of saturated fat between the intervention and control groups but purchase of healthier discounted foods was higher in the intervention than in the control groups and these effects were sustained 12 months after the intervention. Tailored nutrition education alone however showed no effect on the purchase of healthier food choices.	
Anderson 2001 (weak) [38]	Lower levels of fat, higher levels of fibre and higher levels of fruit and vegetable serves were observed in the intervention group compared to the control group.	There was improvement in some mediator factors but not in others.
Anderson 1997 (weak) [37]	Fibre, fruit and vegetable intake increased in the intervention sites from baseline to post-test compared to that for the control sites.	
Phipps 2014 (moderate) [35]	Purchase of fruit and vegetables was higher in the intervention group compared to the control group.	
Winett, 1997 (moderate) [36]	Lower levels of fat, higher levels of fibre and higher levels of fruit and vegetable serves in the intervention group were shown compared to the control group.	
Kristal (weak)^2^ [43]	There was no effect on reported purchase of fruit and vegetable.	

^1^Classification of the study regarding risk of bias between brackets.

^2^Long-term intervention.

*Aimed at customers*:

Six studies [22, 35–38, 43] offered monetary incentives to customers (Table 7). Monetary incentives included price discounts of 12.5% [22], 50% [35] or store coupon/vouchers [36–38, 43] valued from 50 cents per coupon [36, 43] to $10 per week [38]. Five studies were conducted in the USA and one in New Zealand [22] (Additional file 5: Table S5). Three studies were conducted in small towns [37, 38, 43], one in a rural town [36], one in eight supermarkets in three cities [22], and one in one supermarket in one city [35] (Additional file 5: Table S5). The number of study participants ranged from 58 [35] to 1104 [22] and were mostly female (Additional file 5: Table S5). Fruits and vegetables were the target food groups in two studies [35, 43] (Additional file 4: Table S4). One study [22] targeted 1032 food products from multiple food groups and used the Heart Foundation’s Tick program criterion to identify healthier food options (Additional file 4: Table S4). Three other studies applied interventions aimed at reduced fat products from multiple food groups and did not report the criteria to identify healthy food options. The Social Cognitive Theory informed the nutrition intervention in three studies [36–38], the Consumer Information Processing model was used in one study [43] and no theoretical framework was reported for the other two studies [22, 35]. One study [43] applied a long-term intervention including both interactive and non-interactive activities. All five other studies applied a short-term intervention. The non-interactive activities were available monthly at point-of-sale in one study [22] and the interactive activities were conducted at each shopping time by computer-generated messages [36–38] or via written reports [35] or via a shopping list tailored by individual shoppers’ usual food purchases [22] (Additional file 5: Table S5).

There were insufficient strong studies applying nutrition education plus monetary incentive offered to customers including a short-term or long-term interactive or non-interactive intervention to draw clear conclusions on their impact on purchase and/or intake of healthier food options or on the mediating factors that might effect the primary outcome (Table 7).

*Aimed at both store-owners and customers:*

Three studies [29, 42, 44] were included in this category. All studies were undertaken in low-income areas of the USA and included mostly females (Additional file 5: Table S5). Two studies [42, 44] applied a similar intervention that aimed to decrease sugar and fat intake from multiple food groups. The third study [29] included an intervention aimed at increasing fruit and vegetables. The criterion to identify healthier food options was reported in only one study [44] (Additional file 4: Table S4). Two studies [42, 44] included a long-term intervention consisting of five 2-month themed phases over 10 months and one study [29] included a short-term intervention (Additional file 3: Table S3). The theoretical framework to develop the nutrition education strategy is mentioned in one study [42] (Additional file 4: Table S4). All three studies used both non-interactive and interactive activities (Additional file 4: Table S4). The frequency in delivery of the nutrition education interactive activities was mentioned in two studies [29, 42] and was twice per month in one study [42] and monthly in the other study [29] (Additional file 3: Table S3). The value of the monetary incentive offered to store-owners to cover initial stocking costs of targeted products was reported in two studies and ranged from $25 - $50 per intervention phase [44] for one study and was $1000 in the other study [29]. The monetary incentive offered to customers included incentive cards (e.g., buy three get one free) and coupons for discounts on promoted food items [42, 44]. The value of these incentives was not mentioned. Incentives of $10 were provided to customers at each assessment point in one study [29].

There were insufficient studies to draw clear conclusions on the impact of nutrition education plus monetary incentive offered to both store-owners and customers on purchase and/or intake of healthier food options, or on mediating factors that might effect the primary outcome (Table 8).

**Table 8:** **Primary and secondary outcomes of studies applying short- and long- term interactive interventions classified as nutrition education plus monetary incentives aimed at both store-owners and customers**

References	Primary outcomes	Secondary outcomes
Song (weak)^1^ [44]	Recalled stocking and sales scores were higher in intervention stores than in control stores.	There was improvement in some specific mediator factors but not in overall mediator factors.
Gittelsohn 2010b (weak) [42]		No changes were shown in most mediator factors between intervention and control stores.
Ayala 2013 (moderate)^2^ [29]	The intervention increased availability of vegetables but not fruit.	Self-efficacy for consuming more fruits decreased.

^1^Classification of the study regarding risk of bias between brackets.

^2^Short-term intervention.

### 5. Nutrition intervention through vending machines

Four studies were undertaken in eight [30] to 55 [41] vending machines in schools [17, 20, 30, 41] and worksites [41] (Additional file 5: Table S5). Three studies [17, 30, 41] were conducted in the USA and one in the Netherlands [20] (Additional file 5: Table S5). Three studies [17, 20, 41] targeted low fat foods and low calorie foods and one targeted diet beverages [30] (Additional file 4: Table S4). All studies included short-term interventions of approximately five weeks and one [20] included three successive 6-week intervention phases (Additional file 3: Table S3). All studies included non-interactive activities only (Additional file 4: Table S4).

Nutrition education alone was applied in two studies [30, 41] (Table 9). There were insufficient studies to draw clear conclusions on the impact of nutrition education alone through vending machines on purchase and/or intake of healthier food options, or on mediating factors that might effect the primary outcome (Table 9).

**Table 9:** **Primary and secondary outcomes of studies applying short-term non-interactive interventions through vending machines including nutrition education alone**

References	Primary outcomes	Secondary outcomes
Bergen (moderate)^1^ [30]	Sales of sugar-sweetened soft drink were less in the intervention stores compared to the control stores but no difference in sales was observed at 2 weeks follow-up.	
French (weak) [41]	Promotion of healthier food options was associated with greater sales but not with sales volume. The total number of healthier food options did not differ by promotion condition.	

^1^Classification of the study regarding risk of bias between brackets.

Enhanced availability of healthier food options was applied in two studies [17, 20] (Table 10). There were insufficient studies to draw clear conclusions on the impact of enhanced availability of healthier food options through vending machines on purchase and/or intake of healthier food options, or on mediating factors that might effect the primary outcome (Table 10).

**Table 10:** **Primary and secondary outcomes of studies applying short-term non-interactive interventions through vending machines including enhanced availability of healthier food options**

References	Primary outcomes	Secondary outcomes
Fiske (strong)^1^ [17]	There was no difference in sales of healthier food options between intervention and control groups.	
Kocken (strong) [20]	Higher sales of healthier food options were observed in intervention groups compared to control groups.	

^1^Classification of the study regarding risk of bias between brackets.

Enhanced availability of healthier food options plus nutrition education was applied in two studies [17, 20] (Table 11). There were insufficient studies to draw clear conclusions on the impact of enhanced availability of healthier food options plus nutrition education through vending machines on purchase and/or intake of healthier food options, or on mediating factors that might effect the primary outcome (Table 11).

**Table 11:** **Primary and secondary outcomes of studies applying short-term non-interactive interventions through vending machines including enhanced availability of healthier food options plus nutrition education**

References	Primary outcomes	Secondary outcomes
Fiske (strong)^1^ [17]	There was no difference in the sales of healthier food options between intervention and control groups.	
Kocken (strong) [20]	Higher sales of healthier food options were observed in the intervention groups compared to the control groups.	

^1^Classification of the study regarding risk of bias between brackets.

Monetary incentive alone was applied in one study [41] (Table 12). There were insufficient studies to draw clear conclusions on the impact of monetary incentives alone through vending machines on purchase and/or intake of healthier food options, or on factors that might mediate the effect of the intervention (Table 12).

**Table 12:** **Primary and secondary outcomes of the study applying short-term non-interactive interventions through vending machines including monetary incentive alone**

Reference	Primary outcomes	Secondary outcomes
French (weak)^1^ [41]	Higher sales of healthier food options were observed with higher price reductions of 25% and 50% but no difference was observed with a 10% price reduction.	

^1^Classification of the study regarding risk of bias between brackets.

Monetary incentive plus nutrition education was applied in one study [41] (Table 13). There were not enough studies to draw clear conclusions on the impact of monetary incentives plus nutrition education through vending machines on purchase and/or intake of healthier food options, or on factors that might mediate the effect of the intervention (Table 13).

**Table 13:** **Primary and secondary outcomes of the study applying short-term non-interactive interventions through vending machines including monetary incentive plus nutrition education**

Reference	Primary outcomes	Secondary outcomes
French (weak)^1^ [41]	Price reduction was associated with an increase in healthier food option sales volume but nutrition education was unrelated to the change in healthier food option sales volume.	

^1^Classification of the study regarding risk of bias between brackets.

Monetary incentive plus nutrition education plus enhanced availability of healthier food options was applied in one study [20] (Table 14). There were insufficient studies to draw clear conclusions on the impact of monetary incentives plus nutrition education plus enhanced availability of healthier food options through vending machines on purchase and/or intake of healthier food options, or on mediating factors that might effect the primary outcome (Table 14).

**Table 14:** **Primary and secondary outcomes of the study applying short-term non-interactive interventions through vending machines including monetary incentive plus nutrition education plus enhanced availability of healthier food options**

Reference	Primary outcomes	Secondary outcomes
Kocken (strong)^1^ [20]	Higher sales of healthier food options were observed with higher price reductions of 25% and 50% but no difference was observed with a 10% price reduction.	

^1^Classification of the study regarding risk of bias between brackets.

In summary, there were insufficient studies to draw clear conclusions of the impact of any of the interventions applied for a short-term period through vending machines on purchase and/or intake of healthier food options, or on mediating factors that might effect the primary outcome.

### 6. Nutrition intervention through shopping online

The one study [34] classified as moderate in this category was undertaken in an online supermarket service in Sydney, Australia. Most of the participants were female and around forty years of age (Additional file 5: Table S5). The study targeted low fat products during a 5-month period (Additional file 4: Table S4). The intervention included interactive activities by providing tailored nutrition advice and opportunity to swap certain products for a healthier option at point-of-sale (Additional file 3: Table S3).

There were insufficient studies to draw clear conclusions on the impact of nutrition intervention through shopping online on purchase and/or intake of healthier food options, or on mediating factors that might effect the primary outcome (Table 15).

**Table 15:** **Primary and secondary outcomes of the study applying short-term interactive interventions through shopping online**

Reference	Primary outcomes	Secondary outcomes
Huang (moderate)^1^ [34]	Higher sales of healthier food options were observed in the intervention group compared to the control group.	

^1^Classification of the study regarding risk of bias between brackets.

Overall, it was not possible to conduct a meta-analysis as the studies varied widely in terms of approaches, types, duration and intensity of interventions and outcomes reported. Even within interventions types there were not enough studies of similar intervention duration and intensity.

## Discussion

Thirty-two studies met inclusion criteria for this review. The interventions were grouped into six types: i) Nutrition education and promotion alone through supermarkets/stores; ii) Nutrition education plus enhanced availability of healthy foods; iii) Monetary incentive alone; iv) Nutrition education plus monetary incentives; v) Nutrition intervention through vending machines; and, vi) Nutrition intervention through shopping online. There was considerable heterogeneity between the nature of the interventions, intervention duration, intensity of nutrition education activities delivered, and outcomes reported. As a result of this heterogeneity we were unable to make any assessment of the overall effectiveness of point-of-sale interventions in increasing purchase and/or intake of healthier food options. We were however able to analyse and describe the evidence of effect based on the different categories and subcategories and highlight where there are gaps in evidence and where more research is warranted to inform intervention and public policy in an area where there is considerable potential to influence customer food choice and thereby impact on health outcomes.

The evidence of this review indicates that monetary incentives offered to customers for a short-term seem promising in increasing purchase of healthier food options when the intervention is applied by itself in stores or supermarkets. This is consistent with that reported by An et al. [10]. There were also few studies that examined mediating factors that might effect primary outcomes.

Monetary incentives, through discounts, coupons, vouchers, and loans have been shown to be effective in increasing purchase of healthier food options [5, 10]. A key finding from the Gittelsohn et al. review [5] was a need for combined environmental (such as monetary incentive) and behavioral (such as nutrition education) approaches in small-store interventions. We found that there were an insufficient number of studies examining monetary incentive and nutrition education that met our inclusion criteria. As these two intervention types require different resources in terms of development, implementation and evaluation, it is imperative in informing practice and policy that further research examines the cost of these two intervention types in relation to size of the effect. Future research in this area should also consider: i) use of standard terminology for the type of food outlet; ii) clearly describing and examining intervention type, duration and intensity; iii) explicitly describing the theory of intervention; and iv) use of standard and consistent outcome measures and data collection methodologies. Another recommendation for future research is to assess the effect of the study context. The study context can influence intervention effect, for example, an approach to influence food spending through enhancing availability of healthier food options may have a different impact in food outlets in low-income, disadvantaged or remote areas that may be less likely to stock a wide range of healthier food options, compared to areas of higher socio-economic status. Only six studies in this review provided information on population socio-economic context, and eleven on participant socio-economic status.

A limitation of this review is the potential for publication bias. Other studies may exist that would meet this review’s criteria but have not been submitted or accepted for publication and therefore were not identified in this review. The likelihood of this is difficult to judge. Another limitation is the inclusion of studies reported in three languages only. Other studies published in other languages were not considered for inclusion in this review. Overcoming, detecting and correcting for publication bias is problematic. Funnel plots allow review authors to make a visual assessment of whether small-study effects may be present in a meta-analysis. Due to the range of outcome measures and data collection methodologies it was not possible to undertake a meta-analysis or funnel plots. This limits the ability to adequately consider overall effect.

## Conclusions

This study highlights the many different dimensions of interventions that have been examined in the endeavour to influence customer food choice. Although numerous studies at point-of-sale have been undertaken, there is a wide range of different types of interventions and different mechanisms by which various interventions are expected to work.

The evidence from this review indicates that monetary incentives offered to customers for a short-term seem promising in increasing purchase of healthier food options when the intervention is applied by itself in stores or supermarkets. There were insufficient studies to draw clear conclusions on the effectiveness in increasing purchase and/or intake of healthier food options when any of the interventions described in this review were applied. There were insufficient studies that examined mediating factors that might effect primary outcomes of relevant intervention to make an assessment of their impact in increasing purchase and/or intake of healthier food options.

This review suggests that there is a gap in good quality studies addressing several types of relevant point-of-sale interventions to increase purchase and/or intake of healthier food options. Due to the importance of the relationship between population health and dietary improvement there is a need for more well designed studies on the effectiveness of the different types of point-of-sale interventions to encourage healthier eating and to improve health outcomes. There is also a need for studies examining the mediating factors that might effect the primary outcomes of these interventions. There is also a need for study interventions to be more clearly defined in terms of their theoretical basis for changing behaviour and measurement of relevant outcomes.

## Electronic supplementary material

Additional file 1: Table S1: Excluded studies and reasons for exclusion. (DOCX 106 KB)

Additional file 2: Table S2: Bias risk assessment of the included studies. (DOCX 62 KB)

Additional file 5: Table S5: Characteristics of the included studies in alphabetical order. (DOCX 124 KB)

Additional file 3: Table S3: Interactive and non-interactive nutrition education activity type, frequency and duration by intervention type. (DOCX 70 KB)

Additional file 4: Table S4: Targeted products, criteria to identify healthy products, theoretical framework, intervention characteristics, data collection method and reported outcomes, of included studies by intervention type. (DOCX 78 KB)
