# Supplementary material for: Nutrition interventions at point-of-sale to encourage healthier food purchasing: a systematic review
Source: BMC Public Health. 2014 Sep 5;14:919. doi: 10.1186/1471-2458-14-919 (PMC4180547; doi:10.1186/1471-2458-14-919)
Supplement: Supplementary file 1 — Additional file 1: Table S1: Excluded studies and reasons for exclusion. (DOCX 106 KB) [file 12889_2014_7082_MOESM1_ESM.docx]

## Additional file 1: Table S1 - Excluded studies and reasons for exclusion

| **References** | **Reason for exclusion** |
| --- | --- |
| Adams, 2012 [[1](#_ENREF_1)] | No control group |
| An, 2013 [[2](#_ENREF_2)] | No control group |
| Anderson, 1998 [[3](#_ENREF_3)] | Intervention not described and seems to be outside store. |
| Andreyeva, 2011 [[4](#_ENREF_4)] | No intervention, it is a process evaluation |
| Andreyeva, 2012 [[5](#_ENREF_5)] | No control group |
| Beneke, 1988 [[6](#_ENREF_6)] | Intervention outside eligible food outlets and aimed at obese and overweight participants |
| Beydoun, 2008 [[7](#_ENREF_7)] | No intervention, it is a survey |
| Blakely 2011 [[8](#_ENREF_8)] | Same study reported in Ni Mhurchi. Only data by ethnicity reported. |
| Block, 2010 [[9](#_ENREF_9)] | Intervention outside eligible food outlets |
| Butler 2011 [[10](#_ENREF_10)] | No statistical analysis presented for the primary outcome |
| Caldwell, 2008 [[11](#_ENREF_11)] | No control group |
| Carter, 1995 [[12](#_ENREF_12)] | Intervention not aimed to increase purchase of healthy products |
| Cavanaugh 2014 [[13](#_ENREF_13)] | No control group |
| Condrasky 2010 [[14](#_ENREF_14)] | No control group |
| Cranage, 2003 [[15](#_ENREF_15)] | Intervention outside eligible food outlets |
| Cranage, 2004 [[16](#_ENREF_16)] | Intervention outside eligible food outlets |
| Cristall, 1999 [[17](#_ENREF_17)] | No intervention, it is a process evaluation |
| Curran, 2005 [[18](#_ENREF_18)] | No intervention |
| Dannefer, 2012 [[19](#_ENREF_19)] | No control group |
| Dixon, 2006 [[20](#_ENREF_20)] | No intervention |
| Dougherty 1990 [[21](#_ENREF_21)] | No control group |
| Dwivedi, 1997 [[22](#_ENREF_22)] | No control group |
| Eldridge, 1997 [[23](#_ENREF_23)] | Intervention outside eligible food outlets |
| Epstein, 2006 [[24](#_ENREF_24)] | Not aimed to improve nutrition value of food selected by consumers in food stores |
| Freedman, 2010 [[25](#_ENREF_25)] | No control group |
| French, 1997 [[26](#_ENREF_26)] | No control group |
| French 2010 [[27](#_ENREF_27)] | Data poorly described without statistical analysis. Unclear intervention effect due to variation of two components at the same time (price and availability) |
| French, 2003 [[28](#_ENREF_28)] | No intervention, it is a review |
| Fulkerson, 2004 [[29](#_ENREF_29)] | Intervention outside eligible food outlets |
| Geliebter 2013 [[30](#_ENREF_30)] | Aimed at obese participants |
| Giesen, 2012 [[31](#_ENREF_31)] | No real purchase, only hypothetical purchase |
| Gittelsohn, 2007 [[32](#_ENREF_32)] | Abstract |
| Gittelsohn, 2006 [[33](#_ENREF_33)] | No intervention |
| Gittelsohn, 2007 [[34](#_ENREF_34)] | No intervention, it is a process evaluation |
| Gittelsohn, 2010 [[35](#_ENREF_35)] | No intervention (process)(there are 3, process evaluation of Baltimore Healthy stores) |
| Gittelsohn 2013 [[36](#_ENREF_36)] | No intervention results |
| Gorton, 2010 [[37](#_ENREF_37)] | No control group |
| Goulet, 2008 [[38](#_ENREF_38)] | Intervention outside eligible food outlets |
| Gustafon, 2012 [[39](#_ENREF_39)] | Intervention outside eligible food outlets and aimed at weight loss |
| Han-Markey, 2012 [[40](#_ENREF_40)] | No control group |
| Hoffman, 2009 [[41](#_ENREF_41)] | No intervention, it is a process evaluation |
| Holmes, 2012 [[42](#_ENREF_42)] | Inappropriate analysis of the interrupted Time Series design |
| Horgen, 2002 [[43](#_ENREF_43)] | Intervention outside eligible food outlets |
| Hunt et al., 1990 [[44](#_ENREF_44)] | No control group |
| Izumi, 2011 [[45](#_ENREF_45)] | No intervention |
| Jacobson, 2000 [[46](#_ENREF_46)] | No intervention, it is a review |
| Jansen, 2010 [[47](#_ENREF_47)] | Intervention outside eligible food outlets |
| Jennings, 2012 [[48](#_ENREF_48)] | No control group |
| Jeter, 2010 [[49](#_ENREF_49)] | No control group |
| Kahn, 2003 [[50](#_ENREF_50)] | No control group |
| Kubik, 2010 [[51](#_ENREF_51)] | No intervention, it is a cross-sectional study |
| Kubik, 2011 [[52](#_ENREF_52)] | Intervention outside eligible food outlets |
| Lafferty, 2006 [[53](#_ENREF_53)] | No control group |
| Lang, 2000 [[54](#_ENREF_54)] | No control group |
| Lee, 1996 [[55](#_ENREF_55)] | No control group |
| Lee, 1995 [[56](#_ENREF_56)] | No statistical analysis presented for the primary outcome |
| Levy, 1987 [[57](#_ENREF_57)] | No control group |
| Light, 1989 [[58](#_ENREF_58)] | No intervention, it is a process evaluation |
| Macaskill [[59](#_ENREF_59)] | No intervention |
| Martin, 2012 [[60](#_ENREF_60)] | Cross-sectional design |
| Morland, 2010 [[61](#_ENREF_61)] | No intervention |
| Mullis, 1988 [[62](#_ENREF_62)] | No control group |
| Nederkoorn, 2011 [[63](#_ENREF_63)] | No real purchase only hypothetical purchase |
| Ni Mhurchu, 2007 [[64](#_ENREF_64)] | Pilot study published later |
| O’Loughlin, 1996 [[65](#_ENREF_65)] | No control group |
| Paine-Andrews, 1996 [[66](#_ENREF_66)] | No control group |
| Podolsky, 1998 [[67](#_ENREF_67)] | Abstract |
| Pollard, 2008 [[68](#_ENREF_68)] | No intervention |
| Potter, 1990 [[69](#_ENREF_69)] | No intervention |
| Powell, 2009 [[70](#_ENREF_70)] | No intervention, it is a review |
| Reger, 1998 [[71](#_ENREF_71)] | No intervention, it is a cross-sectional study |
| Reid, 2004 [[72](#_ENREF_72)] | No intervention |
| Rose, 2009 [[73](#_ENREF_73)] | No intervention |
| Rowell 2013 [[74](#_ENREF_74)] | Not aimed at increasing purchase of healthier food options |
| Rowley, 2000 [[75](#_ENREF_75)] | Intervention aimed at high risk cohort |
| Rowley, 2001 [[76](#_ENREF_76)] | No intervention, it is a cross-sectional study |
| Rowse, 1994 [[77](#_ENREF_77)] | No intervention, it is a survey |
| Ruzansky, 1998 [[78](#_ENREF_78)] | Intervention eligible outside food outlets |
| Sacks, 2009 [[79](#_ENREF_79)] | No control group |
| Scott, 1991 [[80](#_ENREF_80)] | No control group |
| Scrimgeour, 1994 [[81](#_ENREF_81)] | No control group |
| Song, 2007 [[82](#_ENREF_82)] | Abstract |
| Sutherland, 2010 [[83](#_ENREF_83)] | No control group |
| Sutherland, 2010 [[84](#_ENREF_84)] | No intervention, it is a comment |
| Taylor, 2001 [[85](#_ENREF_85)] | Intervention eligible outside food outlets |
| Thorndike, 2012 [[86](#_ENREF_86)] | Intervention eligible outside food outlets |
| Vermeer, 2010 [[87](#_ENREF_87)] | Intervention eligible outside food outlets |
| Waterlander et al., 2012 [[88](#_ENREF_88)] | No real purchase only hypothetical purchase |
| Waterlander et al., 2012 [[89](#_ENREF_89)] | No real purchase only hypothetical purchase |
| Weinehall, 2001 [[90](#_ENREF_90)] | No intervention, it is a methodology description |
| Wiggers, 2001 [[91](#_ENREF_91)] | No control group |
| Winett, 1988 [[92](#_ENREF_92)] | Intervention not well described, it is not mentioned how many stores were involved in the study |
| Wootan, 2005 [[93](#_ENREF_93)] | Same study reported in Reger 1999 and Booth Butterfield 2004 and does not add new findings |
| No author, 2010 [[94](#_ENREF_94)] | No intervention, it is a process evaluation |

**References**

1. Adams J, Halligan J, Watson DB, Ryan V, Penn L, Adamson AJ, White M: **The Change4Life Convenience Store Programme to Increase Retail Access to Fresh Fruit and Vegetables: A Mixed Methods Process Evaluation**. *PLoS ONE* 2012, **7**(6):1-7.

2. An R, Patel D, Segal D, Sturm R: **Eating Better for Less: A National Discount Program for Healthy Food Purchases in South Africa**. *American journal of health behavior* 2013, **37**(1):56-61.

3. Anderson AS, Cox DN, McKellar S, Reynolds J, Lean MEJ, Mela DJ: **Take Five, a nutrition education intervention to increase fruit and vegetable intakes: impact on attitudes towards dietary change**. *British Journal of Nutrition* 1998, **80**(2):133-140.

4. Andreyeva T, Middleton AE, Long MW, Luedicke J, Schwartz MB: **Food retailer practices, attitudes and beliefs about the supply of healthy foods**. *Public Health Nutr* 2011:1-8.

5. Andreyeva T, Luedicke J, Middleton AE, Long MW, Schwartz MB: **Positive Influence of the Revised Special Supplemental Nutrition Program for Women, Infants, and Children Food Packages on Access to Healthy Foods**. *Journal of the Academy of Nutrition & Dietetics* 2012, **112**(6):850-858.

6. Beneke WM, Davis CH, Vander Tuig JG: **Effects of a behavioral weight-loss program food purchases: instructions to shop with a list**. *Int J Obes* 1988, **12**(4):335-342.

7. Beydoun MA, Powell LM, Wang YF: **The association of fast food, fruit and vegetable prices with dietary intakes among US adults: Is there modification by family income?** *Social Science & Medicine* 2008, **66**(11):2218-2229.

8. Blakely T, Ni Mhurchu C, Jiang Y, Matoe L, Funaki-Tahifote M, Eyles HC, Foster RH, McKenzie S, Rodgers A: **Do effects of price discounts and nutrition education on food purchases vary by ethnicity, income and education? Results from a randomised, controlled trial**. *Journal of epidemiology and community health* 2011:aheadofprint.

9. Block JP, Chandra A, McManus KD, Willett WC: **Point-of-purchase price and education intervention to reduce consumption of sugary soft drinks**. *Am J Public Health* 2010, **100**(8):1427-1433.

10. Butler R, Tapsell L, Lyons-Wall P: **Trends in purchasing patterns of sugar-sweetened water-based beverages in a remote Aboriginal community store following the implementation of a community-developed store nutrition policy**. *Nutrition & Dietetics* 2011, **68**(2):115-119.

11. Caldwell EM, Miller Kobayashi M, DuBow WM, Wytinck SM: **Perceived access to fruits and vegetables associated with increased consumption**. *Public Health Nutr* 2009, **12**(10):1743-1750.

12. Carter N, Kindstedt A, Melin L: **Increased sales and thefts of candy as a function of sales promotion activities: Preliminary findings**. *Journal of applied behavior analysis* 1995, **28**(1):81-82.

13. Cavanaugh E, Green S, Mallya G, Tierney A, Brensinger C, Glanz K: **Changes in food and beverage environments after an urban corner store intervention**. *Prev Med* 2014.

14. Condrasky MD, Frost S, Lee A, Simmons S, Hrabski T: **What's cooking? A culinary nutrition research program with dietetic interns**. *Topics in Clinical Nutrition* 2010, **25**(3):280-288.

15. Cranage DA, Conklin MT, Bordi PL: **CAN YOUNG ADULTS BE INFLUENCED TO EAT HEALTHIER SNACKS: THE EFFECTS OF CHOICE AND NUTRITIONAL INFORMATION ON TASTE, SATISFACTION AND INTENT TO PURCHASE**. *Foodservice Research International* 2003, **14**(2):125-137.

16. Cranage DA, Conklin MT, Lambert CU: **Effect of Nutrition Information in Perceptions of Food Quality, Consumption Behavior and Purchase Intentions**. *Journal of Foodservice Business Research* 2004, **7**(1):43-61.

17. Cristall A: **Healthy Foods, Healthy Hearts**. *Journal of Nutrition Education* 1999, **31**(1):60.

18. Curran S, Gittelsohn J, Anliker J, Ethelbah B, Blake K, Sharma S, Caballero B: **Process evaluation of a store-based environmental obesity intervention on two American Indian Reservations**. *Health Education Research* 2005, **20**(6):719-729.

19. Dannefer R, Williams DA, Baronberg S, Silver L: **Healthy Bodegas: Increasing and Promoting Healthy Foods at Corner Stores in New York City**. *American Journal of Public Health* 2012, **102**(10):e27-e31.

20. Dixon H, Scully M, Parkinson K: **Pester power: snackfoods displayed at supermarket checkouts in Melbourne, Australia**. *Health Promot J Austr* 2006, **17**(2):124-127.

21. Dougherty MF, Wittsten AB, Guarino MA: **PROMOTING LOW-FAT FOODS IN THE SUPERMARKET USING VARIOUS METHODS, INCLUDING VIDEOCASSETTES**. *Journal of the American Dietetic Association* 1990, **90**(8):1106-1108.

22. Dwivedi G, Harvey J, St. John L, Close A: **Taste-test booth: an innovative tool in health promotion**. *J Can Diet Assoc* 1997, **58**(2):90-93.

23. Eldridge AL, Snyder MP, Faus NG, Kotz K: **Development and evaluation of a labeling program for low-fat foods in a discount department store foodservice area**. *Journal of Nutrition Education* 1997, **29**(3):159-161.

24. Epstein LH, Dearing KK, Handley EA, Roemmich JN, Paluch RA: **Relationship of mother and child food purchases as a function of price: A pilot study**. *Appetite* 2006, **47**(1):115-118.

25. Freedman MR, Connors R: **Point-of-purchase nutrition information influences food-purchasing behaviors of college students: a pilot study**. *Journal of the American Dietetic Association* 2010, **110**(8):1222-1226.

26. French SA, Jeffery RW, Story M, Hannan P, Snyder MP: **A pricing strategy to promote low-fat snack choices through vending machines**. *American Journal of Public Health* 1997, **87**(5):849-851.

27. French SA, Hannan PJ, Stat M, Harnack LJ, Mitchell NR, Toomey TL, Gerlach A: **Pricing and Availability Intervention in Vending Machines at Four Bus Garages**. *Journal of Occupational & Environmental Medicine* 2010, **52**(1S):S29-S33.

28. French SA: **Pricing effects on food choices**. *Journal of Nutrition* 2003, **133**(3):841S-843S.

29. Fulkerson JA, French SA, Story M, Nelson H, Hannan PJ: **Promotions to increase lower-fat food choices among students in secondary schools: description and outcomes of TACOS (Trying Alternative Cafeteria Options in Schools)**. *Public Health Nutrition* 2004, **7**(5):665-674.

30. Geliebter A, Ang I, Bernales‐Korins M, Hernandez D, Ochner CN, Ungredda T, Miller R, Kolbe L: **Supermarket discounts of low‐energy density foods: Effects on purchasing, food intake, and body weight**. *Obesity* 2013, **21**(12):E542-E548.

31. Giesen JC, Havermans RC, Nederkoorn C, Jansen A: **Impulsivity in the supermarket. Responses to calorie taxes and subsidies in healthy weight undergraduates**. *Appetite* 2012, **58**(1):6-10.

32. Gittelsohn J, Suratkar S, Hee-Jung S, Rasooly I, Sacher S, Rajan R, Sharma S, Anliker J: **Process evaluation of a food store-based intervention for low income residents of Baltimore City**. *FASEB Journal* 2007, **21**(5):A305-A305.

33. Gittelsohn J, Dyckman W, Tan ML, Boggs MK, Frick KD, Alfred J, Winch PJ, Haberle H, Palafox NA: **Development and implementation of a food store-based intervention to improve diet in the Republic of the Marshall Islands**. *Health Promot Pract* 2006, **7**(4):396-405.

34. Gittelsohn J, Dyckman W, Frick KD, Boggs MK, Haberle H, Alfred J, Vastine A, Palafox N: **A pilot food store intervention in the Republic of the Marshall Islands**. *Pacific health dialog* 2007, **14**(2):43-53.

35. Gittelsohn J, Suratkar S, Song HJ, Sacher S, Rajan R, Rasooly IR, Bednarek E, Sharma S, Anliker JA: **Process evaluation of Baltimore Healthy Stores: a pilot health intervention program with supermarkets and corner stores in Baltimore City**. *Health Promot Pract* 2010, **11**(5):723-732.

36. Gittelsohn J, Dennisuk LA, Christiansen K, Bhimani R, Johnson A, Alexander E, Lee M, Lee SH, Rowan M, Coutinho AJ: **Development and implementation of Baltimore Healthy Eating Zones: a youth-targeted intervention to improve the urban food environment**. *Health Educ Res* 2013, **28**(4):732-744.

37. Gorton D, Carter J, Cvjetan B, Ni Mhurchu C: **Healthier vending machines in workplaces: both possible and effective**. *N Z Med J* 2010, **123**(1311):43-52.

38. Goulet J, Lamarche B, Lemieux S: **A nutritional intervention promoting a Mediterranean food pattern does not affect total daily dietary cost in North American women in free-living conditions**. *Journal of Nutrition* 2008, **138**(1):54-59.

39. Gustafson AA, Sharkey J, Samuel-Hodge CD, Jones-Smith JC, Cai J, Ammerman AS: **Food Store Environment Modifies Intervention Effect on Fruit and Vegetable Intake among Low-Income Women in North Carolina**. *Journal Of Nutrition And Metabolism* 2012, **2012**:932653-932653.

40. Han-Markey TL, Wang L, Schlotterbeck S, Jackson EA, Gurm R, Leidal A, Eagle K: **A public school district's vending machine policy and changes over a 4-year period: Implementation of a national wellness policy**. *Public Health* 2012, **126**(4):335-337.

41. Hoffman JA, Morris V, Cook J: **THE BOSTON MIDDLE SCHOOL-CORNER STORE INITIATIVE: DEVELOPMENT, IMPLEMENTATION, AND INITIAL EVALUATION OF A PROGRAM DESIGNED TO IMPROVE ADOLESCENTS' BEVERAGE-PURCHASING BEHAVIORS**. *Psychology in the Schools* 2009, **46**(8):756-766.

42. Holmes AS, Estabrooks PA, Davis GC, Serrano EL: **Effect of a Grocery Store Intervention on Sales of Nutritious Foods to Youth and Their Families**. *Journal of the Academy of Nutrition & Dietetics* 2012, **112**(6):897-901.

43. Horgen KB, Brownell KD: **Comparison of price change and health message interventions in promoting healthy food choices**. *Health Psychology* 2002, **21**(5):505-512.

44. Hunt MK, Lefebvre C, Hixson ML, Banspach SW, Assaf AR, Carleton RA: **Pawtucket Hearth Health Program Point-of-Purchase Nutrition Education Program in Supermarkets**. *American Journal of Public Health* 1990, **80**(6):730-732.

45. Izumi BT, Zenk SN, Schulz AJ, Mentz GB, Wilson C: **Associations between Neighborhood Availability and Individual Consumption of Dark-Green and Orange Vegetables among Ethnically Diverse Adults in Detroit**. *Journal of the American Dietetic Association* 2011, **111**(2):274-279.

46. Jacobson MF, Brownell KD: **Small taxes on soft drinks and snack foods to promote health**. *Am J Public Health* 2000, **90**(6):854-857.

47. Jansen E, Mulkens S, Jansen A: **How to promote fruit consumption in children. Visual appeal versus restriction**. *Appetite* 2010, **54**(3):599-602.

48. Jennings A, Cassidy A, Winters T, Barnes S, Lipp A, Holland R, Welch A: **Positive effect of a targeted intervention to improve access and availability of fruit and vegetables in an area of deprivation**. *Health & Place* 2012, **18**(5):1074-1078.

49. Jetter KM, Cassady DL: **Increasing fresh fruit and vegetable availability in a low-income neighborhood convenience store: a pilot study**. *Health Promot Pract* 2010, **11**(5):694-702.

50. Kahn RF, O'Sullivan P, Vannatta PM: **Supermarket tour: The effect of presentation mode on nutrition knowledge and attitudes**. *Family Medicine* 2003, **35**(10):721-725.

51. Kubik MY, Wall M, Shen L, Nanney MS, Nelson TF, Laska MN, Story M: **State but not District Nutrition Policies Are Associated with Less Junk Food in Vending Machines and School Stores in US Public Schools**. *Journal of the American Dietetic Association* 2010, **110**(7):1043-1048.

52. Kubik MY, Lytle LA, Farbakhsh K: **School and District Wellness Councils and Availability of Low-Nutrient, Energy-Dense Vending Fare in Minnesota Middle and High Schools**. *Journal of the American Dietetic Association* 2011, **111**(1):150-155.

53. Lafferty A, Marquart L, Reicks M: **Hunting for whole grains: A supermarket tour**. *Journal of Nutrition Education and Behavior* 2006, **38**(3):197-198.

54. Lang JE, Mercer N, Tran D, Mosca L: **Use of a supermarket shelf-labeling program to educate a predominately minority community about foods that promote heart health**. *Journal of the American Dietetic Association* 2000, **100**(7):804-809.

55. Lee AJ, Hobson V, Katarski L: **Review of the nutrition policy of the Arnhem Land Progress Association**. *Australian & New Zealand Journal of Public Health* 1996, **20**(5):538-544.

56. Lee AJ, Bonson APV, Yarmirr D, Odea K, Mathews JD: **SUSTAINABILITY OF A SUCCESSFUL HEALTH AND NUTRITION PROGRAM IN A REMOTE ABORIGINAL COMMUNITY**. *Medical Journal of Australia* 1995, **162**(12):632-635.

57. Levy AS, Stokes RC: **EFFECTS OF A HEALTH PROMOTION ADVERTISING CAMPAIGN ON SALES OF READY-TO-EAT CEREALS**. *Public Health Reports* 1987, **102**(4):398-403.

58. Light L, Tenney J, Portnoy B, Kessler L, Rodgers AB, Patterson B, Mathews O, Katz E, Blair JE, Evans SK *et al*: **Eat for health: a nutrition and cancer control supermarket intervention**. *Public Health Reports* 1989, **104**(5):443-450.

59. Macaskill L, Paul A, Pitcher B, Cullinane D: **Consumer Acceptance of Lower Fat Foods**. *Journal of Nutrition Education* 1998, **30**:410A.

60. Martin KS, Havens E, Boyle KE, Matthews G, Schilling EA, Harel O, Ferris AM: **If you stock it, will they buy it? Healthy food availability and customer purchasing behaviour within corner stores in Hartford, CT, USA**. *Public Health Nutrition* 2012, **15**(10):1973-1978.

61. Morland KB: **An Evaluation of a Neighborhood-Level Intervention to a Local Food Environment**. *American Journal of Preventive Medicine* 2010, **39**(6):E31-E38.

62. Mullis RM, Pirie P: **Lean meats make the grade--a collaborative nutrition intervention program**. *Journal of the American Dietetic Association* 1988, **88**(2):191-195.

63. Nederkoorn C, Havermans RC, Giesen JC, Jansen A: **High tax on high energy dense foods and its effects on the purchase of calories in a supermarket. An experiment**. *Appetite* 2011, **56**(3):760-765.

64. Ni Mhurchu C, Blakely T, Wall J, Rodgers A, Jiang Y, Wilton J: **Strategies to promote healthier food purchases: a pilot supermarket intervention study**. *Public Health Nutr* 2007, **10**(6):608-615.

65. O'Loughlin J, Ledoux J, Barnett T, Paradis G: **La Commande du Coeur ("Shop for Your Heart"): a point-of-choice nutrition education campaign in a low-income urban neighborhood**. *American journal of health promotion : AJHP* 1996, **10**(3):175-178.

66. Paine-Andrews A, Francisco VT, Fawcett SB, Johnston J, Coen S: **Health marketing in the supermarket: using prompting, product sampling, and price reduction to increase customer purchases of lower-fat items**. *Health Mark Q* 1996, **14**(2):85-99.

67. Podolsky MS, Gregoire MB, Lafferty LJ, Lipson S: **The Impact of Marketing Healthy Items on Customers' Perceptions of a University/Medical Center Cafeteria**. *Journal of the American Dietetic Association* 1998, **98**(9, Supplement 1):A104-A104.

68. Pollard CM, Lewis JM, Binns CW: **Selecting interventions to promote fruit and vegetable consumption: from policy to action, a planning framework case study in Western Australia**. *Aust New Zealand Health Policy* 2008, **5**:27.

69. Potter JD, Graves KL, Finnegan JR, Mullis RM, Baxter JS, Crockett S, Elmer PJ, Gloeb BD, Hall NJ, Hertog J *et al*: **The Cancer and Diet Intervention Project: a community- based intervention to reduce nutrition-related risk of cancer**. *Health Education Research* 1990, **5**(4):489-503.

70. Powell LM, Chaloupka FJ: **Food Prices and Obesity: Evidence and Policy Implications for Taxes and Subsidies**. *Milbank Quarterly* 2009, **87**(1):229-257.

71. Reger B, Wootan MG, Booth-Butterfield S, Smith H: **1% or less: A community-based nutrition campaign**. *Public Health Reports* 1998, **113**(5):410-419.

72. Reid RD, D'Angelo MES, Dombrow CA, Heshka JT, Dean TR: **The heart and stroke foundation of Canada's health check food information program - Modelling program effects on consumer behaviour and dietary practices**. *Canadian Journal of Public Health-Revue Canadienne De Sante Publique* 2004, **95**(2):146-150.

73. Rose D, Hutchinson PL, Bodor JN, Swalm CM, Farley TA, Cohen DA, Rice JC: **Neighborhood Food Environments and Body Mass Index The Importance of In-Store Contents**. *American Journal of Preventive Medicine* 2009, **37**(3):214-219.

74. Rowell AE, Binkley M, Alvarado C, Thompson L, Burris S: **Influence of food safety training on grocery store employees’ performance of food handling practices**. *Food Policy* 2013, **41**:177-183.

75. Rowley KG, Daniel M, Skinner K, Skinner M, White GA, O'Dea K: **Effectiveness of a community-directed `healthy lifestyle' program in a remote Australian Aboriginal community**. *Australian & New Zealand Journal of Public Health* 2000, **24**(2):136.

76. Rowley KG, Su Q, Cincotta M, Skinner M, Skinner K, Pindan B, White GA, O'Dea K: **Improvements in circulating cholesterol, antioxidants, and homocysteine after dietary intervention in an Australian Aboriginal community**. *American Journal of Clinical Nutrition* 2001, **74**(4):442-448.

77. Rowse T, Scrimgeour D, Knight S, Thomas D: **Food-purchasing behaviour in an Aboriginal community. 1. Results of a survey**. *Aust J Public Health* 1994, **18**(1):63-67.

78. Ruzansky AS, Whiting S, Dobson JD: **Determining the Use and Perceived Effectiveness of a Point-of-Purchase Cafeteria Nutrition Education Program**. *Canadian journal of dietetic practice and research : a publication of Dietitians of Canada = Revue canadienne de la pratique et de la recherche en dietetique : une publication des Dietetistes du Canada* 1998, **59**(2):90-92.

79. Sacks G, Rayner M, Swinburn B: **Impact of front-of-pack 'traffic-light' nutrition labelling on consumer food purchases in the UK**. *Health Promotion International* 2009, **24**(4):344-352.

80. Scott JA, Begley AM, Miller MR, Binns CW: **Nutrition education in supermarkets: the Lifestyle 2000 experience**. *Aust J Public Health* 1991, **15**(1):49-55.

81. Scrimgeour D, Rowse T, Knight S: **Food-purchasing behaviour in an Aboriginal community. 2. Evaluation of an intervention aimed at children**. *Aust J Public Health* 1994, **18**(1):67-70.

82. Song H-J, Gittelsohn J, Suratkar S, Sharma S, Frick K, Miyong K: **Development and implementation of an intervention for Korean American corner stores in Baltimore City**. *FASEB Journal* 2007, **21**(5):A305-A305.

83. Sutherland LA, Kaley LA, Fischer L: **Guiding Stars: the effect of a nutrition navigation program on consumer purchases at the supermarket**. *American Journal of Clinical Nutrition* 2010, **91**(4).

84. Sutherland L, Kaely L, Fischer L: **Comment on Guiding Stars: The Effect of a Nutrition Navigation Program on Consumer Purchases at the Supermarket**. *Nutrition in Clinical Practice* 2010, **25**(5):560-561.

85. Taylor GA: **Coupon response in services**. *Journal of Retailing* 2001, **77**(1):139-151.

86. Thorndike AN, Sonnenberg L, Riis J, Barraclough S, Levy DE: **A 2-Phase Labeling and Choice Architecture Intervention to Improve Healthy Food and Beverage Choices**. *American Journal of Public Health* 2012, **102**(3):527-533.

87. Vermeer WM, Alting E, Steenhuis IHM, Seidell JC: **Value for money or making the healthy choice: the impact of proportional pricing on consumers’ portion size choices**. *European Journal of Public Health* 2010, **20**(1):65-69.

88. Waterlander WE, Steenhuis IH, de Boer MR, Schuit AJ, Seidell JC: **Introducing taxes, subsidies or both: The effects of various food pricing strategies in a web-based supermarket randomized trial**. *Prev Med* 2012.

89. Waterlander WE, Steenhuis IH, de Boer MR, Schuit AJ, Seidell JC: **The effects of a 25% discount on fruits and vegetables: results of a randomized trial in a three-dimensional web-based supermarket**. *The International Journal Of Behavioral Nutrition And Physical Activity* 2012, **9**:11-11.

90. Weinehall L, Hellsten G, Boman K, Hallmans G: **Prevention of cardiovascular disease in Sweden: the Norsjo community intervention programme--motives, methods and intervention components**. *Scand J Public Health Suppl* 2001, **56**:13-20.

91. Wiggers J, Considine R, Hazell T, Haile M, Rees M, Daly J: **Increasing the practice of health promotion initiatives by licensed premises**. *Health Education & Behavior* 2001, **28**(3):331-340.

92. Winett RA, Kramer KD, Walker WB, Malone SW, Lane MK: **MODIFYING FOOD PURCHASES IN SUPERMARKETS WITH MODELING, FEEDBACK, AND GOAL-SETTING PROCEDURES**. *Journal of Applied Behavior Analysis* 1988, **21**(1):73-80.

93. Wootan MG, Reger-Nash B, Booth-Butterfield S, Cooper L: **The cost-effectiveness of 1% or less media campaigns promoting low-fat milk consumption**. *Prev Chronic Dis* 2005, **2**(4):A05.

94. **Health tips. Outsmart your supermarket**. *Mayo Clin Health Lett* 2010, **28**(1):3.
