# Supplementary material for: Nutrition interventions at point-of-sale to encourage healthier food purchasing: a systematic review
Source: BMC Public Health. 2014 Sep 5;14:919. doi: 10.1186/1471-2458-14-919 (PMC4180547; doi:10.1186/1471-2458-14-919)
Supplement: Supplementary file 2 — Additional file 2: Table S2: Bias risk assessment of the included studies. (DOCX 62 KB) [file 12889_2014_7082_MOESM2_ESM.docx]

## Additional file 2: Table S2 - Bias risk assessment of the included studies

| Reference | Selection bias (represent population) | Study design (randomisation) | Confounders (differences between groups at baseline) | Blinding (assessors and participants aware) | Data collection (valid reliable) | Attrition (drop-outs) | Overall^1^ |
| --- | --- | --- | --- | --- | --- | --- | --- |
| **Nutrition education and promotion alone** | | | | | | | |
| Achabal [[1](#_ENREF_1)] | moderate | strong | weak | moderate | strong | strong | moderate |
| Booth-Butterfield (2004) [[2](#_ENREF_2)] | weak | moderate | weak | moderate | weak | moderate | weak |
| Connell (2001) [[3](#_ENREF_3)] | moderate | strong | strong | moderate | weak | strong | moderate |
| Ernst 1986 [[4](#_ENREF_4)] | weak | strong | weak | weak | strong | moderate | weak |
| Foster (2014) [[5](#_ENREF_5)] | moderate | strong | weak | moderate | strong | strong | moderate |
| Jeffery (1982) [[6](#_ENREF_6)] | strong | strong | strong | moderate | strong | strong | strong |
| Levy (1985) [[7](#_ENREF_7)] | moderate | strong | strong | moderate | strong | strong | strong |
| Milliron (2012) [[8](#_ENREF_8)] | strong | strong | strong | moderate | moderate | moderate | strong |
| Ni Mhurchi et al. (2010)^2^ [[9](#_ENREF_9)] | moderate | strong | moderate | strong | strong | strong | strong |
| Reger (1999) [[10](#_ENREF_10)] | strong | strong | moderate | moderate | strong | moderate | strong |
| Reger (2000) [[11](#_ENREF_11)] | strong | strong | moderate | moderate | strong | moderate | strong |
| Rodgers (1994) [[12](#_ENREF_12)] | strong | strong | strong | moderate | strong | moderate | strong |
| Silzer (1994) [[13](#_ENREF_13)] | weak | weak | weak | moderate | moderate | strong | weak |
| Winett (1991) [[14](#_ENREF_14)] | weak | strong | weak | strong | strong | strong | weak |
| Winett (1991) brief report [[15](#_ENREF_15)] | weak | strong | weak | moderate | strong | weak | weak |
| **Nutrition education plus enhanced availability of healthy foods** | | | | | | | |
| Gittelsohn (2010a) [[16](#_ENREF_16)] | strong | strong | strong | moderate | strong | moderate | Strong |
| **Monetary incentive alone** | | | | | | | |
| Herman et al. (2008) [[17](#_ENREF_17)] | moderate | moderate | weak | moderate | strong | moderate | moderate |
|  | | | | | | | |
| Ni Mhurchi et al. (2010) ^2^ [[9](#_ENREF_9)] | moderate | strong | moderate | strong | strong | strong | strong |
| Sturm (2013) [[18](#_ENREF_18)] | moderate | strong | moderate | moderate | strong | strong | strong |
| Waterlander (2013) ^3^ [[19](#_ENREF_19)] | moderate | strong | strong | moderate | strong | moderate | strong |
| **Nutrition education plus monetary incentive** | | | | | | | |
| **To customers** |  |  |  |  |  |  |  |
| Ni Mhurchi et al. (2010) ^2^ [[9](#_ENREF_9)] | moderate | strong | moderate | strong | strong | strong | strong |
| Anderson (1997) [[20](#_ENREF_20)] | weak | strong | strong | weak | strong | weak | weak |
| Anderson (2001) [[21](#_ENREF_21)] | weak | strong | strong | moderate | strong | weak | weak |
| Kristal et al. (1997) [[22](#_ENREF_22)] | weak | strong | strong | moderate | weak | weak | weak |
| Winett (1997) [[23](#_ENREF_23)] | weak | strong | strong | strong | strong | strong | moderate |
| Phipps et al. (2014) [[24](#_ENREF_24)] | weak | strong | moderate | moderate | strong | strong | moderate |
| **To both customers and store owners** | | | | | | | |
| Song et al. (2009) [[25](#_ENREF_25)] | weak | strong | weak | moderate | strong | strong | weak |
| Gittelsohn (2010b) [[26](#_ENREF_26)] | weak | strong | strong | moderate | strong | weak | weak |
| Ayala (2013) [[27](#_ENREF_27)] | strong | strong | weak | moderate | strong | moderate | moderate |
| **Vending machines** | | | | | | | |
| Bergen (2006) [[28](#_ENREF_28)] | weak | strong | moderate | moderate | strong | strong | moderate |
| Fiske (2004) [[29](#_ENREF_29)] | strong | strong | strong | moderate | strong | moderate | strong |
| French et al. (2001) [[30](#_ENREF_30)] | weak | strong | moderate | moderate | strong | weak | weak |
| Kocken (2012) [[31](#_ENREF_31)] | strong | strong | strong | moderate | strong | moderate | strong |
| **Online shopping** | | | | | | | |
| Huang (2006) [[32](#_ENREF_32)] | weak | strong | strong | moderate | strong | strong | moderate |

^1^Overall rating: Strong = no weak; moderate = one weak; weak = 2 or more weak [[33](#_ENREF_33)]; ^2^ Study is mentioned twice because there are two treatments in addition to the control, and each of the treatments fit a different intervention category;

^2^Study mentioned three times because there are three treatments in addition to the control, and each of the treatments fit a different intervention category.

^3^ The study has four treatments. Only the price component was considered because the nutrition education component was outside store which therefore did not meet the inclusion criteria.

1. Achabal DD, McIntyre SH, Bell CH, Tucker N: **The Effect of Nutrition P-O-P Signs on Consumer Attitudes and Behavior**. *Journal of Retailing* 1987, **63**(1):9.

2. Booth-Butterfield S, Reger B: **The message changes belief and the rest is theory: the "1% or less" milk campaign and reasoned action**. *Preventive Medicine* 2004, **39**(3):581-588.

3. Connell D, Goldberg JP, Folta SC: **An intervention to increase fruit and vegetable consumption using audio communications: In-store public service announcements and audiotapes**. *Journal of Health Communication* 2001, **6**(1):31-43.

4. Ernst ND, Wu M, Frommer P, Katz E, Matthews O, Moskowitz J, Pinsky JL, Pohl S, Schreiber GB, Sondik E *et al*: **Nutrition education at the point of purchase: the foods for health project evaluated**. *Prev Med* 1986, **15**(1):60-73.

5. Foster GD, Karpyn A, Wojtanowski AC, Davis E, Weiss S, Brensinger C, Tierney A, Guo W, Brown J, Spross C *et al*: **Placement and promotion strategies to increase sales of healthier products in supermarkets in low-income, ethnically diverse neighborhoods: a randomized controlled trial**. *The American journal of clinical nutrition* 2014.

6. Jeffery RW, Pirie PL, Rosenthal BS, Gerber WM, Murray DM: **Nutrition education in supermarkets: an unsuccessful attempt to influence knowledge and product sales**. *J Behav Med* 1982, **5**(2):189-200.

7. Levy AS, Matthews O, Stephenson M, Tenney JE, Schucker RE: **The Impact of a Nutrition Information Program on Food Purchases**. *Journal of Public Policy & Marketing* 1985, **4**(1):1-13.

8. Milliron BJ, Woolf K, Appelhans BM: **A point-of-purchase intervention featuring in-person supermarket education affects healthful food purchases**. *J Nutr Educ Behav* 2012, **44**(3):225-232.

9. Ni Mhurchu C, Blakely T, Jiang YN, Eyles HC, Rodgers A: **Effects of price discounts and tailored nutrition education on supermarket purchases: a randomized controlled trial**. *American Journal of Clinical Nutrition* 2010, **91**(3):736-747.

10. Reger B, Wootan MG, Booth-Butterfield S: **Using mass media to promote healthy eating: A community-based demonstration project**. *Preventive Medicine* 1999, **29**(5):414-421.

11. Reger B, Wootan MG, Booth-Butterfield S: **A comparison of different approaches to promote community-wide dietary change**. *American Journal of Preventive Medicine* 2000, **18**(4):271-275.

12. Rodgers AB, Kessler LG, Portnoy B, Potosky AL, Patterson B, Tenney J, Thompson FE, Krebs-Smith SM, Breen N, Mathews O *et al*: **"Eat for Health": A Supermarket Intervention for Nutrition and Cancer Risk Reduction**. *American Journal of Public Health* 1994, **84**(1):72-76.

13. Silzer JS, Sheeshka J, Tomasik HH, Woolcott DM: **AN EVALUATION OF SUPERMARKET SAFARI NUTRITION EDUCATION TOURS**. *Journal of the Canadian Dietetic Association-Revue De L Association Canadienne Des Dietetistes* 1994, **55**(4):179-183.

14. Winett RA, Moore JF, Wagner JL, Hite LA, Leahy M, Neubauer TE, Walberg JL, Walker WB, Lombard D, Geller ES *et al*: **Altering shoppers' supermarket purchases to fit nutritional guidelines: an interactive information system**. *J Appl Behav Anal* 1991, **24**(1):95-105.

15. Winett RA, Wagner JL, Moore JF, Walker WB, Hite LA, Leahy M, Neubauer T, Arbour D, Walberg J, Geller ES *et al*: **An experimental evaluation of a prototype public access nutrition information system for supermarkets**. *Health Psychology* 1991, **10**(1):75-78.

16. Gittelsohn J, Vijayadeva V, Davison N, Ramirez V, Cheung LWK, Murphy S, Novotny R: **A Food Store Intervention Trial Improves Caregiver Psychosocial Factors and Children's Dietary Intake in Hawaii**. *Obesity* 2010, **18**:S84-S90.

17. Herman DR, Harrison GG, Afifi AA, Jenks E: **Effect of a targeted subsidy on intake of fruits and vegetables among low-income women in the special supplemental nutrition program for women, infants, and children**. *American Journal of Public Health* 2008, **98**(1):98-105.

18. Sturm R, An R, Segal D, Patel D: **A cash-back rebate program for healthy food purchases in South Africa: results from scanner data**. *Am J Prev Med* 2013, **44**(6):567-572.

19. Waterlander WE, de Boer MR, Schuit AJ, Seidell JC, Steenhuis IH: **Price discounts significantly enhance fruit and vegetable purchases when combined with nutrition education: a randomized controlled supermarket trial**. *The American journal of clinical nutrition* 2013, **97**(4):886-895.

20. Anderson ES, Winett RA, Bickley PG, Walberg-Rankin J, Moore JF, Leahy M, Harris CE, Gerkin RE: **The Effects of a Multimedia System in Supermarkets To Alter Shoppers' Food Purchases: Nutritional Outcomes and Caveats**. *Journal of Health Psychology* 1997, **2**(2):209-223.

21. Anderson ES, Winett RA, Wojcik JR, Winett SG, Bowden T: **A computerized social cognitive intervention for nutrition behavior: Direct and mediated effects on fat, fiber, fruits, and vegetables, self-efficacy, and outcome expectations among food shoppers**. *Annals of Behavioral Medicine* 2001, **23**(2):88-100.

22. Kristal AR, Goldenhar L, Muldoon J, Morton RF: **Evaluation of a supermarket intervention to increase consumption of fruits and vegetables**. *American Journal of Health Promotion* 1997, **11**(6):422-425.

23. Winett RA, Anderson ES, Bickley PG, Walberg-Rankin J, Moore JF, Leahy M, Harris CE, Gerkin RE: **Nutrition for a Lifetime System©: A multimedia system for altering food supermarket shoppers' purchases to meet nutritional guidelines**. *Computers in Human Behavior* 1997, **13**(3):371-392.

24. Phipps EJ, Braitman LE, Stites SD, Singletary SB, Wallace SL, Hunt L, Axelrod S, Glanz K, Uplinger N: **Impact of a Rewards-Based Incentive Program on Promoting Fruit and Vegetable Purchases**. *Am J Public Health* 2014.

25. Song HJ, Gittelsohn J, Kim M, Suratkar S, Sharma S, Anliker J: **A corner store intervention in a low-income urban community is associated with increased availability and sales of some healthy foods**. *Public Health Nutrition* 2009, **12**(11):2060-2067.

26. Gittelsohn J, Song HJ, Suratkar S, Kumar MB, Henry EG, Sharma S, Mattingly M, Anliker JA: **An urban food store intervention positively affects food-related psychosocial variables and food behaviors**. *Health education & behavior : the official publication of the Society for Public Health Education* 2010, **37**(3):390-402.

27. Ayala GX, Baquero B, Laraia BA, Ji M, Linnan L: **Efficacy of a store-based environmental change intervention compared with a delayed treatment control condition on store customers' intake of fruits and vegetables**. *Public Health Nutr* 2013, **16**(11):1953-1960.

28. Bergen D, Yeh MC: **Effects of energy-content labels and motivational posters on sales of sugar-sweetened beverages: Stimulating sales of diet drinks among adults study**. *Journal of the American Dietetic Association* 2006, **106**(11):1866-1869.

29. Fiske A, Cullen KW: **Effects of promotional materials on vending sales of low-fat items in teachers' lounges**. *Journal of the American Dietetic Association* 2004, **104**(1):90-93.

30. French SA, Jeffery RW, Story M, Breitlow KK, Baxter JS, Hannan P, Snyder MP: **Pricing and promotion effects on low-fat vending snack purchases: The CHIPS study**. *American Journal of Public Health* 2001, **91**(1):112-117.

31. Kocken PL, Eeuwijk J, Kesteren NMCV, Dusseldorp E, Buijs G, Bassa-Dafesh Z, Snel J: **Promoting the Purchase of Low-Calorie Foods From School Vending Machines: A Cluster-Randomized Controlled Study**. *Journal of School Health* 2012, **82**(3):115-122.

32. Huang A, Barzi F, Huxley R, Denyer G, Rohrlach B, Jayne K, Neal B: **The effects on saturated fat purchases of providing internet shoppers with purchase- specific dietary advice: a randomised trial**. *PLoS Clin Trials* 2006, **1**(5):e22.

33. EPHPP: **Quality Assessment Tool for Quantitative Studies Dictionary**. 2009.
