# Supplementary material for: Nutrition interventions at point-of-sale to encourage healthier food purchasing: a systematic review
Source: BMC Public Health. 2014 Sep 5;14:919. doi: 10.1186/1471-2458-14-919 (PMC4180547; doi:10.1186/1471-2458-14-919)
Supplement: Supplementary file 3 — Additional file 5: Table S5: Characteristics of the included studies in alphabetical order. (DOCX 124 KB) [file 12889_2014_7082_MOESM3_ESM.docx]

**Additional file 5: Table S5 – Characteristics of the included studies in alphabetical order**

| **Reference** | **Design, setting, store number, participant number and characteristics** | **Intervention** | **Outcomes** |
| --- | --- | --- | --- |
| Achabal [[1](#_ENREF_1)] | RCT, 372 stores in three metropolitan areas of USA, Dallas, San Francisco and Washington DC and 283 participants from 12 stores in San Francisco Bay area. | Three treatments randomly provided to triplets of stores as similar as possible (with regards to store size and county) for 4 weeks included:   - control group (no intervention) - purchase and nutrition information including point-of-purchase (POP) signage (3½x6 inches) for placement over six items (carrots, broccoli, cabbage, cauliflower, kiwifruit, tomatoes) in the produce department during the test period. It contained pictures of food item, selection advice, calorie content and list of nutrients. - Purchase information only and no information about nutrition.   Consumer attitude survey (13 items), completed by 85% of customers intercepted was used to measure: i) perception towards nutrition; ii) consumer attitudes of signage. | Analysis was performed to estimate the influence of i) signage treatment (control, plain sign and nutrition sign); ii) commodity factor; and, iii) the week factor. The baseline period was used as a covariate to remove extraneous variation in sales during test period. There was no significant impact of the signage across sales of the six commodities (F=0.480, p<0.904) nor did the effectiveness of the signage vary significantly from one week to the next during the test period, (F=0.285, p<0.945). However, differences in customer attitudes of signage were shown for different treatments: 3.5, 3.6 and 3.9 (1 = very poor and 5 = very good) for control stores, stores with plain signs, and stores with nutrition signs, respectively.  Only 5.8% of customers interviewed reported seeing the signage and even less actually read it. |
| Anderson (2001) [[2](#_ENREF_2)] | RCT, 5 supermarkets in small towns in south-western, Virginia including 296 participants, 96% were female, 92% were white, had a median income of $35000, and had a mean of 14.8 years of education | Participants recruited in 5 supermarkets by brief face-to-face contact followed by a mail back of enrolment materials including a demographic survey and at least 4 weeks of annotated food shopping receipts. After enrolling, participants continued to send food shopping receipts and completed a food frequency questionnaire (FFQ) and a food beliefs survey after the intervention. Four to 6 months after intervention, participants completed a final FFQ and submitted 6 weeks of food shopping receipts. On recruitment, participants were randomly assigned to:   - no-treatment control condition - intervention consisting of 15 weekly segments (10 content segments and 5 segments devoted to maintenance and delivered through a store ‘kiosk’) focused on increasing purchases and consumption of cruciferous vegetables, fruits, high-fibre cereals, low fat dairy and lean protein sources as well as on decreasing consumption of fat from butter, beef and snacks. Education segments also suggested strategies for monitoring and planning food purchases and meal preparation, and provided opportunities for personalized goal setting and feedback for each targeted food group or behaviour change strategy. Target food coupons from $8 to $12 in total each week were available and redemption was limited to the participants at the kiosk store within 1 week of printing (mean of $34 per participant in coupons during intervention). Completion of all parts of each education segment required 5 to 10 min.   Data collection included food shopping receipts, FFQs, and a composite of the two measures. Values for percentage kcals from fat, fiber grams per 1,000 kcals purchased, and servings of fruits and vegetables (F&V) per 1,000 kcals were derived from each measure. The composite measures were calculated at each assessment point (baseline, post-test, and follow-up). In addition to providing an outcome measure incorporating both nutrition instruments, these composites served as the basis for gauging the magnitude of nutritional change associated with the intervention and for determining nutritional goal attainment. | Intervention group had lower levels of fat in the food shopping receipts, the FFQ, and the composite fat measure. The composite percentage of kcals from fat increased slightly among control group (from 32.74 (6.85) to 33.36 (7.02) and decreased by 9% in the intervention group from 33.24 (7.28) to 30.10 (6.92) from baseline to post-test (F = 15.23 (1, 189) to follow-up (F = 4.535 (1, 155)).  At the post-test, compared to control group, intervention group had higher levels of fibre grams per 1000 kcals on the food shopping receipts (9.96 (4.72) vs 8.95 (4.14), F = 4.438 (1. 255)), on FFQ (10.87 (3.08), vs 8.97 (3.7), 23.487 (1.200)), and in composite fibre measure (10.63 (3.29 vs 8.87 (3.21), F = 19.808 (1.189). Compared to control group, intervention group had higher levels of F&V serving per 1000 kcal in the FFQ (3.52 (1.31) vs 3.37 (1.79), F = 14.436 (1,200) and the composite F & V measure (3.37 (1.79 vs 2.77 (1.27), F = 6.871 (1.189) but not in the food shopping receipts (3.08 (2.61) vs 2.75 (1.95), F 1.571 (1.254). Composite serving per 1000 kcal increased by 20% (from 2.78 (1.06) to 3.37 (1.79) among intervention group and it was maintained at follow-up (3.35 (1.56), F = 3.286 (1.155)). There were no differences between study groups on measures of post-test self-efficacy and outcome expectations except that intervention group had higher levels of self-efficacy for decreasing fat in meals than control group, and higher expectations at post-test that families would accept healthier foods and that these would be satisfying. None of the demographic covariates influenced any of the post-test social cognitive variables.  At post-test, intervention group attained goal for fat (30% or fewer kcals from fat) at almost 4 times the rate of the control group and goal for fibre (25g/d) and F&V a day at about 2 times the rate of control participants at post-test. At follow-up, intervention group attained fat goals at 4 times the rate of the control group but were not more likely to reach fibre and F&V goals. |
| Anderson (1997) [[3](#_ENREF_3)] | RCT, 2 small supermarkets in small towns in southwestern Virginia including 104 participants, 86% were female, 95% were white, had a median age of 40 years, median income of $30000, and had a mean of 16 years of education. House hold size ranged from 1 to 8 members. | Same intervention described in Anderson 2001. Outcome measures were: i) daily per person purchases of grams of fat; ii) grams of fibre, iii) servings of fruit and vegetables.  This re-examination of data was performed as an increase in calories observed in both treatment and control groups may have confounded earlier findings reported by Winnet et al [[4](#_ENREF_4)], that the NLS intervention contributed to significantly lower levels of calories from fat. | There was a general upward trend in supermarket purchases in both study groups. Daily per person grams of fiber from total-foods purchased increased from baseline to posttest among treatment group families (from 7.50 (4.27) to 10.17 (4.19), but not among control group families (9.35 (4.61) to 9.31 (3.77).  Grams of servings of F&V also increased from baseline to posttest among treatment group families (2.45 (1.66) to 3.75 (2.55), but not among control group families (3.34 (2.49) to 3.34 (2.45).  Daily per person servings of F&V also increased from baseline to post-test among treatment group families (1.61 (1.06) to 2.32 (1.48)), but not among control group families (2.02 (1.57) to 2.01 (1.47). Daily per person grams of fat from total foods, dairy foods, meat, cooking & table, snack foods and prepared food and daily grams of fiber from bread and from cereal did not change or increased slightly from baseline to post-test among both intervention and control groups.  The intervention contributed to lower levels of total daily per person fat from post-test food purchases, to lower levels of fat from post-test purchases of table and cooking fats and from purchases of snack foods. The treatment did not influence the daily per person levels of fat in participants’ purchases of dairy, meat or prepared foods. Higher coupon redemption was associated with greater decreases in total, daily per person grams of fat (χ2= 17.26; p=0.002) and greater increases in daily per person servings of F&V (χ2= 11.91;p=0.018), but with no effect on fibre (χ2= 7.81; p=0.10) |
| Ayala (2014) [[5](#_ENREF_5)] | RCT with 9 tiendas meeting the criteria (availability of some F&V, presence of other food services (e.g. butcher, prepared foods) and more than one employee besides the manager) out of 37 audited in Central North Carolina. 179 customers recruited and 119 were available for outcome analysis. Most of the study participants were females  (66 %), young adults (mean age 32 years) and married or living as married (75 %). A majority (83 %) reported living with family in households of approximately four individuals. Over a third had 6 years of education or less. The majority was employed (71 %), including 36% as labourers, and the average household monthly income was approximately $US1500.00. Most rented their home or apartment (74 %) and nearly a quarter were on some sort of food assistance programme. All of the participants were born outside the USA, and specifically 84% were of Mexican origin. On average respondents had lived in the USA for 8 (SD 5) years. | Tiendas in central North Carolina were randomly  assigned to one of two conditions: (i) an environmental change intervention to promote store customers’ intake of F&V; compared with (ii) a delayed treatment control condition, an intervention similar to the one described here but implemented after the follow-up assessment.  Intervention activities were 4 months in duration, including 2 months for employee- and manager-directed activities followed by 2 months of a customer-directed intervention. The intervention focused on the business aspect of store management including employee training, point-of-purchase promotion and merchandising produce, all designed to promote sales of F&V. The 4-month intervention involved: (i) employee and manager trainings; (ii) an 8-week food marketing campaign that included a point-of-purchase component, food demonstrations and an audio-based media campaign; and (iii) equipment to make structural changes.  The training sessions were designed to occur during three 30-min sessions and focused on how to sharpen business skills key to promoting produce sales. basic nutrition information about F&V, how to successfully market produce in their tiendas and how to work with produce distributors to ensure the purchase of high-quality produce, as well as methods for how to store and display produce to maximize sales and minimize waste.  An 8-week food marketing campaign was conducted in each intervention tienda and consisted of food demonstrations, distribution of recipe and business cards with health promotion messages, and an audio novela. Each week two project staff conducted a food demonstration using commonly prepared Latino dishes but modified to include more fruits or vegetables, or new F&V dishes to spark the interest of individuals who may be more inclined to try something new. The food demonstration was accompanied by distribution of traditional recipe cards plus health promoting cards, the size of a business card and billed as such with the tienda’s name. The two tiendas also received posters, price tags, shelf tags, streamers (aka papel picado; a traditional Mexican form of art that consists of a long string of paper-cut designs, commonly used at celebrations such as birthdays), nutrient information tables for produce, and other point-of-purchase materials showcasing the fruit or vegetable promoted that week. Finally, to more fully capture the attention of customers, the campaign included the airing of an audio novela over the store public announcement system that described the journey of eight fruit and vegetable characters from Mexico and Central America to the USA. Images of these characters were included in all print material developed for the campaign.  To initiate structural changes to promote the sales of F&V, each of the intervention tiendas was allocated $US 1000.00 to purchase equipment and other materials. The tiendas requested a buffet bar to display ready-to-eat F&V. The bar was equipped with freezable bins.  Implementation of the structural changes was delayed due to challenges with obtaining and setting up equipment; however, ultimately they were implemented as planned.  Evaluation of the intervention was assessed at the customer and tienda levels. Additional customer changes were examined on behavioural and psychosocial variables. Research assistants consented and then collected baseline data from customers during face-to-face interviews in the tiendas. Telephone interviews were used to complete the post-intervention assessment. All assessments required approximately 45 min to complete. | The intervention condition participants reported consuming nearly an additional daily serving of F&V. The same was not observed among the delayed treatment condition participants. Contrary to what was expected, self-efficacy for purchasing and consuming F&V decreased in the intervention condition and increased in the delayed treatment condition. No other significant group-by-time interactions were observed on the other outcomes including fruit variety, vegetable variety, behavioural strategies for fat, behavioural strategies for fibre and perceived barriers.  There were larger increases in the availability of fresh and canned vegetables in the intervention v. the control tiendas. There were similar decreases in fresh fruits in both study conditions, and similar increases in canned fruits. |
| Bergen (2006) [[6](#_ENREF_6)] | CBA, 8 vending machines located in an American urban college separated by one floor within the main building. The mean number of beverages sold in 8 vending machines ranged from 794.91 during baseline to 994.57 during intervention phase. | The vending machines contained 12 beverage choices including water, diet beverages and sugar-sweetened soft drinks and were randomly assigned to:   - 0 calories, 0 carbohydrate labels on the selection panels - Labels + motivational posters and education information - No changes in the control machines.   The total of all beverages sold and machine revenue were recorded at: baseline (2-week baseline data collection preceded the randomization), intervention (5 weeks) and post-intervention (2 weeks) periods. | Sugar-sweetened soft drink growth less in the intervention labels + motivational posters condition than in the control condition.  Total revenue from all beverage sales increased by 25.12% in the intervention period. Of that increase, 48.54% was due to water bottles sales, 21.98% from diet soft drinks, and 29.48% from sugar-sweetened soft drink sales. Post-intervention, neither sugar-sweetened soft drink nor water and non-energy containing soft drink sales were not significantly different from baseline compared with controls. |
| Booth-Butterfield (2004) [[7](#_ENREF_7)] | CBA, 2 supermarkets in small size communities in West Virginia, USA. West Virginia had high rates of cardiovascular disease and high-fat diet and regular consumption of milk were reported. The relatively small size of West Virginia communities (no metropolitan area larger than 100,000) and their geographic isolation made it possible to conduct relatively inexpensive media interventions. 366 and 285 shoppers completed a survey in pre and 6 weeks later in the intervention stores. At control stores, the figures were 374 and 258. Participants were most female (67%) aged 50.3y and 48% reported some education past high school | Treatments included:   - 1 control community outside the media market - 1 intervention community with paid advertisements and public events for 6 weeks.   Supermarket milk data were collected and randomly selected intervention and comparison community residents were surveyed via telephone to assess milk use during the month immediately before and the month immediately after the campaign. | Store sale data from control community is not mentioned or compared with intervention community but is reported in Reger 1999. Only survey data is reported. . All analyses reported were based only upon participants who completed both pre and post test surveys and reported using high-fat milk at pre-test (135 and 134 at the intervention and comparison).  The intervention community participants reported more switching (34%) than the comparison community (4%), while the intervention community’s low-fat milk share of total milk sales increased from 29% to 46%. The intervention was effective by increasing intention (F = 43.14, p < 0.001) and attitude (F = 30.04, p < 0.001). The intervention increased beliefs about healthiness (F = 9.29, p < 0.003), taste (F = 16.24, p < 0.001), and cost (F = 4.12, p < 0.04) of low-fat milk. The intervention had a direct effect upon attitude (b = 0.312, t = 5.38, p < 0.001), which had an effect upon intention (b = 0.479, t = 9.21. p < 0.00000001), which had an effect on behaviour (b = 0.558, t = 10.22, p < 0.000000001. Attitude had a direct effect on behaviour (b = 0.130, t = 2.42, p < 0.01619) and the largest source of variance switching came from intention, followed by direct effects from the intervention and, finally, direct effects from attitude. Intervention had a direct effect on intention (b = 0.209, t = 3.99. p < 0.00008) and on behaviour (b = 0.141, t = 2.95, p < 0.00343). Attitude had a direct effect on behaviour (b = 0.130, t = 2.42, p < 0.01619) and the largest source of variance switching came from intention, followed by direct effects from the intervention and, finally, direct effects from attitude. Intervention communities reported more milk use, more low-fat milk use, and greater likelihood of purchasing milk in supermarkets. |
| Connell (2001) [[8](#_ENREF_8)] | CBA, 6 stores from a large supermarket chain in Eastern Massachusetts, USA divided into three pairs, matched by geographic area and by median household income of shoppers who used them. One pair was primarily low income and two were mainly middle income. The study was conducted in Northeast and it was not able to generalize to stores in other parts of the country. However, a significant number of low-income individuals were purposely included in the study and there is reason to believe that these results would also apply to individuals with greater economic resources. Because the intended audience included a range of cultures, no attempt was made to target a subpopulation. The study included 374 and 378 shoppers in the intervention and control stores- 78% to 81% were women, nearly half reported incomes of $30,000 or less. | Study aim was to evaluate the effectiveness of two audio formats on knowledge, attitudes and beliefs about fruit and vegetables and intake of these. Six stores were divided into three pairs, matched by geographic area and by median household income. One pair was mainly low income and two were mainly middle income. One store from each pair was randomly selected for the intervention and the other served as the control. A sample of shoppers recruited once over seven consecutive days (up to 18 people per day if they were regular shoppers in target stores, aged 18 years or older and their total purchases were > $25) during the week prior to the intervention were interviewed before and after the intervention period. Participants completed a baseline survey, took home and listened to the audiotapes over the four weeks of the intervention and were interviewed by telephone at the end of the intervention. They received a $10 voucher for purchases at any store in the supermarket chain at the time of the recruitment and again after completing the follow-up interview at the end of the 4-week intervention. The intervention over 4 weeks included 5 a Day messages promoting value of eating F&V through:   - In-store audio public service announcements broadcasted through the in-store every 30 min for 30 to 60s. - Take-home audiotapes (2 each with 1hr program) about F&V preparation and a nutrition knowledge test   Shoppers in the control stores received audiocassettes about relaxation techniques.  Outcome measures were: Knowledge about F&V (7 items), benefits of F&V (9 items), F&V self-reported intake (using a 7-item questionnaire developed for the NCI a Day for Better Health Effort) and behavioural intention (7 items) using means and proportions. | A total of 87.7% out of 374 and 93.7% out of 378 of the shoppers in the intervention and control stores completed both the baseline survey and the telephone follow-up. Of 96% of shoppers who had completed follow-up interviews and had returned to the market where they had originally been interviewed, only marginally more shoppers in the intervention than in the control stores reported hearing an in-store audio related to the value of eating fruits and vegetables (19.7% vs 15.3%). However, shoppers in intervention stores who did hear the audio rated the information segments higher than shoppers in control stores. When asked about their nutrition information sources, approximately 37% of shoppers in both groups said they listened to one or more audiotapes on that topic over the intervention period. Awareness of the meaning of the 5 a Day message was similar and quite high in both the intervention and control groups at pretest. At follow-up, intervention store shoppers were more likely (87.2%) to be aware of the meaning of the 5 a Day slogan compared to control shoppers (76%, F = 16.1, p < 0.01). At baseline, a greater proportion of control group shoppers provided the correct answer to one question. At follow-up, however, intervention store shoppers provided the correct answers more frequently on four of the seven items and achieved higher total scores. At posttest, the total percentage of correct responses increased for both groups and it was greater in intervention store shoppers (15% above baseline) than in the control store shoppers (4.5% above the baseline, F = 10.7, p < 0.001). No differences were observed between the intervention and control groups at baseline or between baseline and follow-up for the total score statements measuring beliefs or attitudes toward food and nutrition concepts. At baseline, 28.5% and 23.1% and at follow-up, 45% and 35% of those in the intervention and control group, respectively reported that they consumed diets “high” or “very high” in fibre. The difference from baseline to follow-up was significant within each of the groups and the magnitude was greater in the intervention group (X^2^ = 4.1, p < 0.05). Average intake of F&V at baseline was similar in the 2 groups (5.39 (4.5) versus 5.38 (4.5)). This persisted when fried potatoes were excluded. At follow-up, reported intake rose both within the intervention stores (F = 9.9, p < 0.01) and when compared with the control stores (F = 2.9, p < 0.05). There were no differences from baseline to follow-up or between the intervention and control groups in the percent of shoppers reporting that they would increase their intakes of fibre or eat more F&V over the next six months. In fact, there was a decline at post-test in the percentage of shoppers reporting intentions to make these positive behaviour changes. |
| Ernst, 1986 [[9](#_ENREF_9)] | CBA, 10 food stores from a major food retailing outlet in Washington area receiving intervention and 10 stores in Baltimore area, USA had store sales data collected.. 2399 respondents completed surveys out of 10930 randomly selected contacts. Across the three stores and 2 time points, the mean age of the survey participants ranged from 41.2 to 43.6, the percentage of male ranged from 8.2% to 21.0%, total number of persons in household ranged from 2.7 to 3 and education level was around 4 in a 6 point scale where 1 is grade school and 6 is postgraduate. | Intervention consisted of: Nutrition education at the point-of-purchase at the intervention stores including:   - a series of four-page brochures (3 million in total over 48 weeks) appeared in the dairy aisle where 95% of shoppers passed every 2 weeks containing new issues, facts about cardiovascular health, nutrition information, practical suggestions for food selection, recipes, ideas about food preparation, tips on eating behaviour, and information about the program itself and aimed to reduce fat, cholesterol and calories in the diet. - 2000 shelf signs placed near targeted food items containing messages consistent with the program. - A graduate series of media cues including advertisements in food sections newspapers, special advertisements - a series of 60-sec radio spots focusing on current topics - A press briefing to introduce the project. - Promotion techniques included displaying window signs, 3000 posters and banners, located in areas where consumers usually stop for service – in the produce, seafood and bakery sections.   Control group received none of the nutrition information materials or media promotion disseminated in the intervention area.  Weekly sales of 246 individual food items were monitored for a 48-week period. Meat and poultry items were collected manually from warehouse shipment records for 52-week period. Three telephone surveys were conducted prior to, in the middle of, and at the end of the study period, in both study cities. Two sets of questions were used to test knowledge of cardio vascular diseases and nutrition issues and food habits.  Outcome measures were: consumer awareness, knowledge and food purchases, before, during and after campaign. | There was no statistically significant difference in food sales between intervention and comparison groups.  There was a weekly increase of 0.02% in low-fat milk sales as a percentage of total milk sales, estimated at 0.96% from 48 to 48.96% in the intervention stores for the 48-week study period. There was a significant increase in knowledge score in the intervention stores (increase of 4%) in correct responses to questions about fat and cholesterol content in foods; an increase of 5.8% increase in questions about dietary fat- cholesterol) and a decrease in knowledge score in the control areas of 2.6% and 1.9% respectively to same two sets of questions. This represented a statistically significant difference in knowledge score between intervention and control communities of 9.2% and 11.2% respectively for these two sets of questions. |
| Fiske, 2004 [[10](#_ENREF_10)] | CBA, 10 vending machines, each located in a different elementary or middle school teachers’ lounge within a southeast Texas school district. The total number of food items sold in baseline (2-weeks), and mean over 4 weeks was 222.5 and 199.8, respectively in the 10 vending machines | The vending machines holding 28 similar snack food items and 5 gum selections were randomly exposed over 4-week periods to:   - 2 control machines holding 5 low fat items (containing 5g or less per product) - 4 intervention “I” machines holding 8 low fat items + labels - 4 intervention “II” machines holding 8 low fat items + labels + large signs with yellow price strips that highlighted the price stickers for the low-fat items. A matching small yellow sign indicating that the yellow items were low-fat was placed on the front glass of the machine above the selection buttons.   Large motivational signs encouraging low-fat selections were also placed on top of the intervention machines.  Vending machines sales were tracked on a weekly basis at each of the 10 locations during a 2-week baseline period and during a 4-week intervention period.  Outcome measures: i) no. of low-fat items sold; ii) total machine revenue. | There was no difference in the number of low-fat snacks sold (F = 0.781, p > 0.05) or in the total dollar sales (F = 1.024, p > 0.05) from low-fat items among control and intervention groups.  There was no difference in total machine revenue by experimental condition (F = 0.14, p > 0.05) as a result of the additional low-fat products and as a result of promotional materials. |
| Foster (2014)[[11](#_ENREF_11)] | RCT in eight urban supermarkets in low-income, high-minority neighbourhoods in Philadelphia and Wilmington, United States. | The purpose of this study was to evaluate the effects of in-store marketing strategies to promote the purchase of specific healthier items. The strategies focused on placement and product availability and targeted 5 food and beverage categories (milk, ready-to-eat cereal, frozen meals, in aisle beverages, and checkout cooler beverages).  The intervention consisted of multiple facings: increased the number of facings of the recommended products; 2) prime placement: placed recommended products at arm/eye level and in the middle of the category aisle and reordered types of milk so that 2% milk was located on the left-hand side of the dairy  case followed by 1%, skim and then whole milk; 3) signage: placed call-out signs with the recommended product’s name and price, and shelf runners below recommended products; and 4) secondary placement: mimicked shelf strategies (1 and 2) in all secondary placements (end caps, dead space stacks, etc). In  addition, other strategies were used as appropriate to the category, including 5) cross promotion (cereal and beverages only): displayed recommended products in 2 product categories together, through dead space stacks and end caps (eg, cereal and bananas, soda and water); and 6) taste-testing (milk only): offered free samples of recommended products to increase shoppers’ exposure to healthier options (1 d/mo for 2–3 h). It is important to note that the strategies were “stealth” with regard to health or nutrition claims, there were no prompts to buy one product instead of another, and there were no reductions in the price of the targeted products. The overall approach was to simply increase the visibility of, and access to, healthier options through increased number and optimal placement of the recommended products, signage, and taste-testing. | Milk sales generally declined over the 6-mo intervention period. In the intervention stores, however, skim milk sales remained relatively stable, whereas sales declined significantly (P = 0.0078 for time-by-treatment interaction) in control stores, and sales of 1% milk improved significantly in intervention stores as compared with control stores (P = 0.0014 for time-by treatment interaction). No significant differences were found between intervention and control stores in the amount of whole or 2% milk purchased.  Overall, the sales of the 2 targeted cereals were relatively stable over the intervention period, and no significant differences were found between intervention and control stores. Intervention stores had increased sales compared with decreases in the control stores for 2 of the 3 targeted frozen meals, turkey dinner and chicken nugget (P = 0.0326 and 0.0074, respectively). For turkey dinner frozen meals, there was a significant decrease in the number of units sold in the control stores and no significant change in the intervention stores. For the frozen chicken nugget dinner, there was a non-significant decrease in sales in control stores, a non-significant increase in intervention stores, and a significant time-by-treatment interaction favouring the intervention stores. No differences were found between the intervention and control stores in sales over time for the third targeted meal, Salisbury steak.  No differences were found between the intervention and control stores in the sales of in-aisle regular or diet versions of the targeted Pepsi product (PepsiCo). Sales of water in the aisle decreased in both the control and intervention stores; however, the decrease was less in the intervention stores (P = 0.0109). Of the 3 types of beverage cooler products analyzed (regular products, diet or low-calorie products, and water), the only difference between groups was in water sales. Water sales increased in the intervention stores and were significantly different from control stores (P = 0.0002), which showed declines over time. |
| French et al. (2001) [[12](#_ENREF_12)] | RCT using a 2 settings 4 pricing and 3 promotion factorial design. Vending machines located in a convenient sample of 12 secondary schools and 12 worksites, in Minneapolis, USA. Overall sales volume averaged across all treatment conditions in the 55 vending machines was 1389 products per machine per period. | 12 treatments: four price levels (0, 10%, 25% and 50% discount), 3 promotion conditions (none, low fat label, low fat label + promotion sign) in 2 settings (schools and worksites) of low fat snacks (< 3g of fat per package) for 12 mo. Ten out of 60 low-fat snacks were available in all machines. Each of the 12 treatment conditions was implemented at each of the 24 sites for a 4-week period in a randomly assigned sequence in such a way that period effects were balanced over experimental conditions during each month. Sales data were recorded continuously throughout the intervention. Outcome measures were: i) proportion of, and ii) absolute turnover of low fat snack items; and iii) net profits. | Over all treatment conditions, the average percentages of low fat snack sold were 12.6% at schools and 16.9% at worksites (F = 12.66, p < 0.02). The average percentage of low-fat snack sales in the equal price/no label condition was 9.9%. Price reduction was significantly associated with percentage of low-fat snack sales (F = 156.89, p < 0.001).  Price reductions of 50%, 25%, and 10% were associated with increases in low-fat snack sales of 93%, 39%, and 9%, respectively. The total number of low fat snacks sold was different by price reduction condition (F = 96.98, p < 0.001)  Number of low-fat snacks sold in the 10% price reduction condition did not differ from the number of low-fat snacks sold in the equal price condition. Price reductions of 25% and 50% were associated with significant increases in the absolute number of low-fat snacks sold relative to the equal price and 10% price reduction conditions. The total number of low-fat snacks sold differed between the 25% and 50% price reduction conditions. There were significant interaction between setting and price reduction (F = 13.9, p < 0.001). The size of the increase in the number of low-fat snack sales in the 50% price reduction condition was slightly larger at schools than at worksites. Promotion of low-fat snacks was associated with greater low-fat snack sales (F = 3.48, p < 0.4). The percentage of low-fat snacks sold in the no-label, label-only, and label-plus-sign conditions were 14.3, 14.5 and 15.4, respectively. Only the label-plus-sign condition differed significantly from the no-label condition in post hoc mean comparisons (p<0.05). Total number of low-fat snacks sold did not differ significantly by promotion condition. Profits per machine per treatment period were significantly higher at schools than at worksites (F = 35.84, p < 0.001). There were no significant main effects for price or promotion on vending machine profits. Sales volume at schools was significantly higher than at worksites (F = 29.10, P < 0.01). Promotion was unrelated to sales volume. However, price reduction was significantly associated with sales volume (F = 11.01, p < 0.01). |
| Gittelsohn (2010a) [[13](#_ENREF_13)] | CBA, 4 communities in two islands, Oahu and Hawaii, USA, with a higher prevalence of Native Hawaiian residents, with populations of 10,506 and 5,748, respectively. Comparison communities on each island had populations of 3,664 and 2,997, respectively. Income levels are low, with >75% of the population below the poverty level. Five stores in two communities were selected as implementation venues and two other communities were selected to act as comparison communities. The majority were female (95%), with a mean age of 41 years, had a mean of 12.5 years of schooling and 34% were unemployed. The children were aged 9.9y and 50% of them were girls. | The intervention comprised 4x6-8 wks with a 1-2-week break between the phases aimed to increase availability of healthy food in stores. The phases were developed in a participative process and targeted healthier beverages, healthier snacks (whole grain, lower sugar cereals, low-fat milk, F&V with low-fat dips), healthier condiments (low-fat dressings) and healthier meals (tuna in water). The intervention included:   - posters - educational displays - shelf labels - 4-6 cooking demonstrations/taste tests per phase at each intervention store, with brochures and recipe cards   Control communities had no intervention  Outcomes were assessed in 116 of 130 required 8-12y-child-caregiver dyads randomly selected from local health centre patient databases or Census 2000 data respondents. Data collection methods included:   - Questionnaires at baseline and post intervention about socio demographic characteristics, FFQ focusing on promoted foods and their less healthy alternatives - Survey tools included a Customer impact questionnaire and a child customer impact questionnaire to assess food-related self-efficacy, intentions, and knowledge. - A single 24-h dietary recall   Healthy eating index (HEI) was determined as the sum of the nine components, each ranging from 0 to 9. The primary study aim was to assess program impact on adult care-givers and children’s psychosocial, food related behaviour and children’s food intake. Variables measured included HEI score; % energy from fat; no. serves of fruit; and, no. serves of vegetables. | No significant differences with respect to demographic characteristics were observed between the intervention and comparison samples, with the exception that there were fewer caregivers in the intervention sample who had worked >40 h a week and they were more likely to be unemployed (p < 0.0001). Thus, employment was controlled in analytic models.  There was improvement in food-related knowledge and in the perception that healthy foods are convenient among caregivers in the intervention sample, and this improvement was significant after adjustment for caregiver, age, sex, education, employment, time period between pre- and post measurements, and other demographic factors (unadjusted P = 0.0970; adjusted P = 0.0171). No impacts were observed in terms of other caregiver psychosocial factors (knowledge, self-efficacy, intention and health belief), behaviours (healthy food purchasing, unhealthy food purchasing and healthy food consumption), or in HEI components or target food consumption for caregivers.  No changes were observed in children’s psychosocial variables. Intervention showed increased total HEI scores, increased HEI grain scores, and total water consumption as compared to control children. These were modest increases. No impact was shown in the gram consumption of any of the other targeted foods. |
| Gittelsohn (2010b) [[14](#_ENREF_14)] | CBA, 17 food stores of different sizes and types to reach different types of consumers in Baltimore, where food suppliers for small corner stores include vendors, supermarkets, and wholesale stores and where the lack of availability of specific healthier foods at corner stores is sometimes linked to lack of these foods at wholesale stores or large chain supermarkets. East and West Baltimore are geographically distinct areas of the city, roughly 2 miles apart, and are separated by a highway. East Baltimore had 96% African American of the population with 57% of the households headed by females and with median income ranging from $11,000 to $13,000. West Baltimore had 91.8% African American of the population with 27.8% of the households headed by females and median income ranging from $10,000 to $15,000, and 21.3% of the households with children have incomes below the poverty line. Both East and West Baltimore have many food sources with a limited range of available foods but an abundance of energy-dense foods high in fat and sugar. Food sources include several supermarkets, full-service and fast-food restaurants, corner stores, and convenience stores. This study included 175 customers, where 91.8% to 96% were African Americans with 27.8% to 57% of the females heading households and with the median income ranging from $10,000 to $15,000. | The intervention over 12 months comprised 4x6-8 wks with a 1-2-week break between the phases aimed to increase availability of healthy food in stores. The phases targeted i) healthier beverages, ii) healthier snacks (whole grain, lower sugar cereals, low-fat milk, F&V with low-fat dips), iii) healthier condiments (low-fat dressings) and iv) healthier meals (tuna in water). The intervention included:   - Interactive nutrition education sessions (cooking demonstrations and taste tests twice a month and promoting product giveaways) - Nutrition education and education material to corner store owners to encourage the stocking of healthier food options and encourage positive interactions between storeowners and their customers. - Communication tools (posters, educational displays, shelf labels and flyers) - Incentive cards and coupons for discounts on promoted food items given to customers. - Wholesaler gift cards and/or provision of small amounts of the target foods for promotion to small store owners to initiate the stocking of healthier foods promoted in each phase   Control communities had no intervention  Outcomes were assessed by:   - Customer Impact Questionnaire including socio demographic characteristics, frequency of food getting (including procuring foods through church, food stamps), food-related self-efficacy, intentions, and nutrition knowledge and label comprehension - A quantitative FFQ focusing on promoted foods and their less healthy alternatives - Intervention exposure evaluation | Only 48% of the original 175 baseline respondents were reinterviewed at follow-up.  Only exposure to the educational displays was significantly higher among intervention respondents compared to the control group. There was no difference between intervention and control respondents on the reported education materials seen at stores. Intervention stores were more visited by respondents than comparison stores and when the number of visits to stores reported by respondents was considered (added), the overall exposure score was higher among intervention respondents than among comparison respondents.  There was no significant difference in changes in most psychosocial factors including knowledge, self-efficacy, and intentions neither from baseline to follow-up nor from between intervention and comparison stores. Respondents in the most exposed quartile showed a positive trend in healthy food intentions as compared to those in the least exposed quartile (p= 0.0663).  Healthy food preparation scores improved in the intervention group compared to the comparison group. Purchasing a promoted food because of a shelf label was more likely among intervention than among comparison participants.  Taste testing showed high attendance and participation. The use of shelf labels for the promotion of target foods was reported as a relatively simple way to encourage purchasing of healthy foods. A range of approaches was reported to be needed when working in small food stores to reinforce key messages and optimize the potential for success. |
| Herman et al. (2008) [[15](#_ENREF_15)] | CBA, 602 postpartum women enrolled at the special Supplementation Nutrition Program for women, infants and children (WIC) at 3 program centres in Los Angeles, California, selected on the basis of similarity regarding caseload, ethnic backgrounds of participants, and geographical proximity of a major supermarket and a year-round farmers’ markets. The mean household income was $1233 per month. The sample was 89.1 % Hispanic, 5.9% African American, 2.8% non-Hispanic White, 1.9% Asian American, and 0.2% American Indian. The average age was 27.5 y with 9.3 y of education and average family size was 4.0 persons. Mean household income was $1233 per month. Participants had lived in the United States for an average of 12.5 y and participated in the WIC on average 2.8 y. BMI averaged 28.1 kg/m^2^. | Following a 2-month monitoring period to document baseline F&V intake, intervention was carried out for 6 mo and participants’ diets were followed for an additional 6 mo.   - Intervention included $10 worth of vouchers per week to buy produce of participants’ choice, issued bimonthly, and that could be spent over the ensuing 2-month period. - Control included coupons value of $13 per month redeemable for disposable diapers, in compensation for their time.   24h dietary multiple pass method was collected 4 times at control sites and 6 times at the intervention sites over 14 months.  Household demographic characteristics were also collected. BMI and food security status were assessed at baseline and final interview.  Study aimed to assess whether an additional economic subsidy for WIC participants for fresh fruit and vegetables would result in an increased consumption. | Intake of serving of F&V per 4186 kJ increased more at the intervention sites from 2.9 to 4.1 whereas at the control sites, the increase was from 2.6 to 3.0 servings (F = 9.75; p < 0.001). Six months after the intervention the increase in F&V intake reported by participants at the intervention sites was sustained (F = 6.66; p = 0.01). Participants at the intervention sites reported eating 4 serving of F&V per 4186 kJ, whereas those at the control site reported an average of 3.1 servings per 4186 kJ. There was no difference in the consumption of fruits alone between intervention and control sites at baseline (F = 2.15; p = 0.12), post intervention (F = 0.95; p = 0.39) or at follow-up. Vegetable consumption alone at the control site at baseline was significantly higher than at the farmers’ market, but not at the supermarket site. When beans and potatoes were removed from the total number of vegetables consumed, there was no difference in the pattern of average consumption of serving of vegetables per 4186 kJ at the intervention and control sites (F = 1.7; p= 0.18). At the end of the intervention, participants at the both intervention sites reported eating more servings of vegetables per 4186 kJ on average than the control site (F = 11.0; p < 0.001) and this difference was maintained when adjusted for multiple comparisons, excluding beans and potatoes (F = 9.33; P < 0.001) and at follow-up. However, after adjustment for multiple comparisons, the difference was statistically significant only when the supermarket site was compared with the control site (F = - 0.40; p = 0.13; for control vs farmers’ market, and F = - 0.59; p = 0.01 for control vs supermarket). When beans and potatoes were excluded from the average servings of vegetables consumed per 4186 kJ, both of the intervention sites had higher consumption of vegetables than the control site, and this difference remained significant after adjustment for multiple comparisons (F = - 0.81; p = 0.07 for control vs farmers’ market and F = -0.75; p = 0.23 for control vs supermarket). |
| Huang (2006) [[16](#_ENREF_16)] | RCT, a total of 4548 individuals were offered participation, 497 were randomised and 456 completed at least one episode of shopping that included one or more of the 524 foods studied in an online supermarket service in Sydney, NSW, Australia, recruited via an online electronic pop-up message were randomised (by a central computerised process with minimisation by age, sex, and number of individuals the food was being purchased for) and 456 completed at least one episode of shopping that included one or more of the 524 foods studied. The mean age was, 39 and 41, 86% and 89% were female, 80% and 80% were married, 90% and 83% were Caucasian or European, 63% and 62% had university education, 82% and 80% were employed, the household income was AUD$105,774 and 112,698 and the mean number of people buying for was 3 and 3, in intervention and control group, respectively.  Around 1 in 10 of consumers who accessed the internet shopping service participated in the study. | Treatment during 5-mo recruitment and follow-up period (average 35d)   - Intervention included advice tailored to the food items customers selected for purchase and opportunity to either retain the product or swap it for the alternative lower in saturated fat. 383 commonly purchased food items that contained 1% or more saturated fat (range 1% to 92%) and offered a suitable lower-fat alternate for each. A simple side-by-side on—screen presentation of the original item and the suggested alternate was used. This was available for 524 discrete food items (items not pre-packaged such as meat were excluded) - Control group received general non-specific advice about how to choose a diet lower in saturated fat.   Percentage of saturated fat and cost of the purchased items among the 524 foods studied was collected.  Outcome measures were: difference in saturated fat (g/100g of food in shopping basket) and difference in cost/ gm. | For the first occasion on which advice was offered, the amount of saturated fat in the food purchased by the intervention group after advice was a mean of 0.66% lower (P < 0.001) than in the corresponding foods purchased by the control group which is equivalent to an approximate 10% reduction in saturated fat content of foods purchased. There was an intervention effect on primary outcome of 0.62% (0.46-0.79, p<0.001) lower. The mean cost per 100g of the food purchased by the intervention group was not different from that in the control group (AUD0.63 (0.58-0.68)/100g vs control AUD0.62 (0.58-0.67)/100g, p=0.19). The foods higher in saturated fat that were most commonly present in the shopping basket prior to advice being offered but absent after the advice had been offered were higher-fat dairy products. The intervention was similarly effective in the different sub-groups studies. 115 participants completed six shopping episodes over the study period. Among this group, reduction in saturated fat achieved was greater in earlier compared to later shopping, suggesting that customers learnt to select lower fat options after receiving advice on just one occasion. Subgroup analyses provided some evidence that the intervention had a greater effect among those with higher BMI and among those >40yrs of age. |
| Jeffery (1982) [[17](#_ENREF_17)] | CBA, 8 stores from a chain of supermarkets headquartered and serving the greater Twin Cities located in the metropolitan area within Minneapolis, St Paul, and suburban areas of Minnesota. These stores, roughly equal in size, served a variety of communities including inner-city areas, middle-class areas, and affluent suburbs. This study included 25 shoppers who were most females (71 to 78%) and younger than 40 years (45% to 60%) or between 50 and 59y (31% to 33%). Only 9% to 22% were older than 60y. | The study aimed to determine the feasibility of conduction effective nutrition education campaigns in the supermarket emphasizing the advantages of reduced fat and total calorie food choices. Stores were grouped into four pairs, matched on shoppers’ education level and one store from each pair was randomised to treatment over a 6-month period:   - Intervention included display of large posters, shelf signs, and brochures, in three waves, throughout the dairy sections of the experimental stores. Wave 1 lasted 12 weeks and had 6 large posters containing pockets with brochures on low-fat diets which were changed at 4-week intervals. Wave 2 lasted 6 weeks and had one large poster displayed with 6 small message cards and six different small recipe tear-off pads. Wave 3 lasted 6 weeks and had one large poster used with 12 small tear-off pads containing a brief description of low-fat dietary recommendations. Materials at stores were checked weekly for damage and replaced when necessary. - Control stores had no education material   Weekly sales data for 25 dairy products (eggs, cottage cheese, yoghurt, milk and cream and frozen desserts) were collected during a 10-month period (July 1979 to April 1980) through wholesalers on the items shipped weekly to the 8 stores. 15, 1 and 9 items were tracked throughout 10, 9 and 6 months of the project and monthly averages were used in the analysis.  25 shoppers completed a 2-min nutrition survey before and after the intervention. (shoppers were randomly selected and there were 25% refusals at pre- and 31% post-intervention). | There were no clear-cut time trends in the sales data. The trends observed in sales data were likely to be unrelated to the intervention. There was a high level of consumer knowledge of high fat foods and risk of cardiovascular disease prior to intervention in both groups. The mean knowledge score increased post-intervention in both control and intervention groups (an increase of 0.75 vs 0.86). The increase in mean scores was slightly greater in the intervention stores but this difference was not statistically significant. pre-test post-test  Control χ2 =12.93 (2.45) χ2 =13.68 (3.26)  Treatment χ2 =12.72 (3.23) χ2 =13.58 (2.51) |
| Kocken et al. (2012) [[18](#_ENREF_18)] | RCT, 28 vending machines located in schools in 4 areas of the Netherlands. Mean sales volume of total food in the experimental and control school averaged for number of students per school was 7.77 and 7.29 in phase 1, 7.08 and 6.38 in phase 2 and 6.61 and 4.82 in phase 3, respectively in 28 vending machines. | - Intervention in 13 vending machines: Three strategies were introduced in 3 successive 6-week phases, finally combining all three strategies in phase 3. Phase 1: Increasing availability of lower-calorie extra products (75% of the products should be favourable (<100kcal per item) or moderately unfavourable (100-170 kcal per item) and no fewer than 25% should be favourable); Phase 2: labelling products (raised thumb in green for favourable, raised index finger for yellow for moderately unfavourable and downturned thumb in red for unfavourable products), posters and flyers containing information on the 3 food groups extra food, basic food, and beverage groups and their favorableness and, iii) Phase 3: reducing the price of lower-calorie products (an average reduction of 10%). Data on stock supplies, orders and sales of all vending machines were collected.  - Control in 15 vending machines: kept their original products and prices and did not use labelling | Mean sales volume of extra food in the experimental and control school averaged for number of students per school was 5.39 and 4.89 in phase 1, 4.59 and 4.1 in phase 2, 4.12 and 2.73 in phase 3, respectively. Mean sales volume of total food in the experimental and control school averaged for number of students per school was 7.77 and 7.29 in phase 1, 7.08 and 6.38 in phase 2 and 6.61 and 4.82 in phase 3, respectively. No baseline data due to limited resources. Mean proportion (sd) of favourable extra foods sold was similar at experimental school (1.6(1.9), 1.9(1.8) and 1.2(1.1)) to that of control schools (0.9(1.5), 0.8(1.1) and 0.8(1.2) at phase 1, 2 and 3 respectively. Proportion of moderately unfavourable foods sold was higher (p<0.05) at the experimental schools (44.2 (15.3), 44.9(13.5) and 46.5(14.0)) than at control schools (20.1(7.8), 18.3(8.1) and 19.7(9.0)) at phases 1, 2 and 3, respectively. Proportion of unfavourable foods sold was lower (p<0.05) at the experimental schools (54.2(16.4), 53.2(14.1) and 52.3(14.1)) than at control schools (78.9(7.6), 80.8(8.3) and 79.4(8.6)) at phases 1, 2 and 3, respectively. Proportion of favourable extra beverages sold was higher at experimental school (38.3(15.9), 37.2(14.0) and 42.2(14.4)) than at control schools (22.4(15.3), 26.0(14.1) and 26.6(15.0)) at phase 1, 2 and 3 respectively, significantly at 10% at phases 1 and 2 and at 5% at phase 3. Proportion of unfavourable extra beverages sold was lower at experimental school (61.7(15.9), 62.8(14.0) and 57.8(14.4)) than at control schools (77.6(15.3), 74.0(14.1) and 73.4(15.0)) at phase 1 (p<0.1), 2 (p<0.1) and 3 (p<0.05) respectively. |
| Kristal et al. (1997) [[19](#_ENREF_19)] | RCT, 8 supermarkets in small towns in Cincinnati, Ohio, separated by at least 35 miles and similar in size. Using tables of random numbers, participants were systematically selected from cash register lines for interview, 84% of the respondents were women, 68% lived in two-adult households and 60% had children living in household. 120 shoppers respondents were evenly distributed four across categories: 18-33, 34-49, 50-64 and 65+. | The aim of the study was to develop a theory-based point of purchase intervention and evaluate alternative approaches for supermarket intervention and education.  Intervention consisted of   - informational flyers and linked supermarket signage. The front side of the flyer listed F&V on sale with a message to eat more and save money. The other side gave recipes and menus using sale items. The flyers were initially weekly and mid-way than biweekly and included a store coupon for 50 cents of any fruit or vegetables. Store signage consisted of highly visible labels that linked the sale F&V items listed on the flyer to their shelf location. - store signage, - awareness raising activities, and food demonstrations twice each month   Control received no intervention.  At baseline and at 1-year post randomization, 120 shoppers’ exit interviews collected data on demographics, shopping habits, recall of store signage, and purchase of F&V.  Data collection methods included:   - monthly sales of F&V on total weight (fresh, frozen and dried) or volume (canned). - Customer recall of use of intervention material and behaviour changes.   A take home survey to mail back to study investigators assessed intake of F&V (FFQ), diet habits, and stages of change in adopting diets high in F&V. The scale score was the mean of six items  The primary outcome: total weight of fruit and vegetables sold each month (this outcome was not reported); Secondary outcome: customer recall and use of intervention materials and behaviour changes associated with shopping. | Intervention effects were 17.7% for recalling a flyer with prices and recipes for F&V and 27.2% for picking one up (P < 0.001). Among intervention respondents, almost 36% had used a 50-cent coupon and 18% had used a recipe. There were no effects on the purchases of F&V or recall of signage. Roughly 70% of the respondents at baseline and 80% at follow-up purchased some type of F&V and there was no difference in the percentage of any F&V purchased between intervention and control site at baseline (71.6 vs 70.4) or at follow up (80.3 vs 78.7). The percentage of frozen F&V was higher (P < 0.05) at follow-up at control site (24.0) compared to intervention site (18.3). Consumption of F&V was higher among intervention respondents at both time points. Statistical significance was not reported in the original study. |
| Levy (1985) [[20](#_ENREF_20)] | Quasi-experimental repeated measures design, 10 stores from a major food retailing outlet in Washington area, USA, which varied in size and socio-economic characteristics, matched on the basis of these characteristics with ten stores from the Baltimore area where the “Special Diet Alert” (SDA) program was not scheduled to be introduced until 1984. The stores span a broad range of size and neighbourhood characteristics. Median household incomes of stores’ shopping areas range from $20,000 to almost $40,000 with accompanying variations in age and ethnic make-up of the neighbourhoods. Stores with neighbourhood income above the median were classified as high socio-economic status (SES) stores; stores with less than median neighbourhood income were classified as low SES stores. | Intervention was broader and longer (2y) than previous programs and used an information delivery system based on computer-generated shelf tags distributed throughout the store, each containing one to three bits of information.  The intervention included:   - Brand-specific nutrition information provided on the shelf next to price information and flagged specific brand name items as low or reduced in one or more of the following: sodium, calories, fat and cholesterol for 400 products from over 20 different food categories - An alert 25-page guide available over 2 years in the store or take-away explaining the guidelines for defining low or reduced dietary components, listing products and quantitative nutrient declarations by food category, and offering dietary hints. - Media campaign using television and radio spots (300 spots mainly in the first 8 weeks) introduced the program to consumers in the area   Control received no intervention  Weekly changes in market shares of 1600 products in 23 separate food categories over the two-year study period were collapsed into 26 four-week intervals spanning the 2-year study period.  Two consumer surveys were conducted before and after the intervention with 100 adult shoppers from each of the 20 test and comparison stores.  Outcome measure: market shares of SDA products in 14 separate food categories over two year study period (ie sales of SDA product and a non-SDA product in same food category) | There were 14 food categories in 16 instances providing valid comparisons between intervention and control groups. Five low calorie/low fat/low cholesterol sectors and three low sodium sectors showed differential growth trends between treatment and control stores consistent with a positive effect of the intervention. Over two years, there was a mean change in market share for city versus trend effect (p<0.05) in butter of 3.81 for changed scores in market share of + 0.27 and + 0.57; in canned fish of 2.41 for changed scores of +0.54 and +0.63; in cottage cheese of 3.04 for changed scores of – 1.06 and -0.71; in mayonnaise of 3.18 for changed scores of – 0.37 and -0.01; and in fruit juice of 3.00 for changed scores of -0.37 and -0.16 for control and intervention, respectively. Low sodium products included frozen vegetables - effect of 3.37 for changed scores of -0.78 and -0.54; soft drinks - effect of 2.04 for changed scores of -0.52 and -0.14; and tomato sauce - effect of 9.15 for changed scores of -0.83 and +0.16 for control and intervention, respectively.  Only fruit juice out of 3 low or reduced calorie/fat/cholesterol sectors (soft drinks, fruit juice, and canned fruit) showed a positive effect of the intervention in achieving low calorie status by reducing sugar. The remaining food categories with low or reduced calorie/fat/cholesterol sectors were mostly dairy categories and all achieved low calorie/low fat status by reducing fat. Four of the categories showed positive program effects.  The long term effect of the program did not seem to be restricted to any particular kind of food category.  Product sector market share increased (p<0.05) on average by 4% to 8% over the 2 years in the intervention group compared to the same product sector market in the control group. The use of SDA shelf labels increased from 12% to 31% in intervention stores compared to no change from 17% to 16% in control stores after 2 year-intervention. Among the special diet shoppers, the use of SDA shelf labels increased (p<0.05) from 11% to 50% in intervention stores compared and from 18% to 23% in control stores.  Reported use of SDA shelf labels was lower for men than women and lower for younger and older shoppers than for middle-aged shoppers (31-64yrs). |
| Milliron (2012) [[21](#_ENREF_21)] | RCT, one supermarket located at Surprise, Arizona, USA part of a large, Arizona-based, family owned supermarket chain with more than 130 stores. Eligibility criteria for study included: 18yrs+, primary household shopper, planning to purchase 15+ items, able to speak and write English, able to shop unassisted, had access to transport and a home refrigerator. 164/500 study inquiries met eligibility criteria. Participants were 41y, 81% were female, the mean BMI was 27.6, 78% were white, 3% black and 14% Hispanic or Latino, 19% had graduate degree, 20 had college and 61% has less than college and the mean number in household was 3. 39% had an annual household income of less than $60,000. Final analysis included 153 (n=70 treatment; n=83 control). Household income level differed between the two groups. The control group reported a greater percentage of the federal poverty guideline compared with those in the intervention group. This was adjusted for in the analysis. No baseline measures were available. The intervention was based on a single shopping episode. | The study aimed to test efficacy of a multi-component point of purchase intervention featuring in-person nutrition education on nutritional composition of food purchases compared to usual practice (shelf signs only).  Point of purchase intervention included brief (10 min), face to face nutrition education and a packaged EatSmart program consisting of store signs and printed materials identifying healthful food choices, including shelf tags (healthier option, heart healthy, low sodium, calcium rich and immune booster) based on Food and Drug Administration labelling regulations and the American Heart Association. A healthful shopping list and a monthly newsletter and recipes were also available in stores.  Control received no intervention  Data were collected on weekdays and weekends for 6 consecutive hours (alternated between 10am and 4pm and between 1pm and 7 pm) during 4 months. Digital photographs of all food and beverage purchases, copies of receipts and field notes of unpackaged items were obtained. Participants also completed a demographic survey and a card on which participants were to mark any of 12 signs they had observed. Outcome measures were: i) purchases of total, saturated and trans fat (g/1000kcal); ii) fruits, vegetables and dark green/ yellow vegetables (serves/1000kcal – derived from people’s shopping baskets. | With the exception of household income level, descriptive characteristics were not significantly different between groups. Sixty-nine percent of the participants randomized to receive the intervention reported the program very or extremely useful, and 26% reported it somewhat useful. Sixty-five percent of participants in the intervention group reported that they would be more likely to shop in a supermarket that offered a healthful shopping program, and 32% reported that it would make no difference in their likelihood to shop. Approximately 65% of the intervention group and 29% of the control group reported seeing the shelf tags. There was no difference between intervention or control group in the purchased total fat (39 vs. 39g/1000kcal, p-0.49), saturated fat (14.0 vs. 14.0, p=0.46), trans fat (1.5 vs. 1.5g/1000kcal, p=0.99) or vegetables servings (1.3 vs. 1.0, p=0.06). However intervention group purchased more fruit (0.7 vs. 0.4 serving/1000kcal, p=0.002) and dark-green/yellow vegetables (0.2 vs. 0.1 serving/1000kcal, p=0.03) than the control group. |
| Ni Mhurchi et al. (2010) [[22](#_ENREF_22)] | RCT – 2x2 factorial blinded; intention to treat analysis, 8 supermarkets from a retail chain in the Lower North Island region of New Zealand. 1104 main household shoppers participated in the study. They were 18yr or older and 52% of the participants had annual household income < NZ$60,000, 42% had > NZ$60,000. Shoppers, mostly female (86%) participants were 44y, 23% were Maori, 9% Pacific and 68% New Zealander European or other ethnicity, 39% had a university degree, 10% had a certificate, 35% had secondary education and 17% had no schooling. 52% had an annual household income < NZ$60,000, 42% had > NZ$60,000 and 6% declined to answer. Number of people in household on average was 2. | The study aimed to evaluate the effect of price discounts and tailored nutrition education on supermarket food and nutrient purchases. A 12-wk baseline data collection phase was followed by a 24-wk intervention phase and a further 24-wk follow-up phase.  Participants were randomised with stratification by ethnicity and household income pretax to:   - Control without any intervention - price discount (12.5%) on healthier supermarket foods (35% of the 3000 top sellers (1032) met the Heart Foundation’s Tick program criteria but discount was applied only to core foods excluding chocolate, sports supplements, baby foods, etc). Printed list of discounted foods was mailed at regular intervals throughout the study and discounts were implemented when Shop cards were scanned at checkouts during the intervention period. The 3000 top selling items coded accounted for 78% of total sales volume and 65% of total expenditure. - tailored nutrition education promoting purchase of healthier supermarket foods suggesting brand-specific healthier alternatives to less healthy foods usually purchased. Printed package of food-group-specific nutrition information consisting of computer-generated messages and shopping lists tailored by individual shoppers’ usual food purchases and supportive generic resources including recipes and recommended serving sizes monthly were mailed for 6 months. - a combination of price discounts and tailored nutrition education.   Outcomes, assessed by using electronic scanner sales data, included percentage energy from saturated fat, total fat, protein, carbohydrate, energy density, sodium, and sugars and quantities of healthier foods purchased. Primary outcome was change in saturated fat at 6 months post randomisation | There were no significant differences in purchased saturated fat between those randomly assigned to receive price discounts and those not assigned to receive discounts at 6 months or 12 mo, nor between tailored nutrition education compared to no education. Saturated fat was similar among participants in all treatments at 6 mo and 12 mo. However change from baseline in saturated fat differed between intervention groups by <0.4% of energy at 6 mo post-randomization and <0.7% at 12mo post-randomization. Overall % fat to energy went down across all groups.  At 6 mo, no effect on fat purchased was detected (-0.02% (95% CI:-0.40%, 0.36%; p=0.91)) with price discount. Likewise no effect was shown with tailored nutrition education compared to no education -0.09% (CI -0.47, 0.30%;p=0.66) Participants receiving the price discount bought 11% more healthier discounted foods compared with those not receiving discounts, at 6 mo post-randomization (mean increase of 0.79kg/wk; 95% CI: 0.43, 1.16; p < 0.001) and this effect was sustained at 12 mo post-randomization (0.38 kg/wk; 95% CI: 0.01, 0.76; P = 0.045; in contrast there was no difference in the purchase of non-discounted, less-healthy foods at 6mo (0.07 kg/wk; 95% CI: -0.15, 0.29; p = 0.56) or 12 mo (0.05 kg/wk; 95% CI: -0.18, 0.27; p = 0.67). There was no overall difference in food expenditure between groups at 6mo post randomization.  Price discount had a positive effect on the amount of purchased healthy foods in most major food subgroups including F&V at 6 mo (0.48kg/wk 95% CI: 0.21, 0.75; p < 0.001) and at 12 mo (0.28kg/wk 95% CI: 0.00, 0.56; p = 0.05), and in cereals and cereal products, meat and meat alternatives, and milk and milk products at 6 mo but not at 12 mo. Participants reported that it took too long to sort through the list and 52% of the participants in the price discount intervention sometimes or never used the list.  In contrast, no effect of tailored nutrition education on foods purchases was evident other than a small increase in healthier cereal purchases at 12 mo, an effect likely to have arisen by chance. |
| Phipps 2014 [[23](#_ENREF_23)] | 4-phase prospective cohort study with 58 customers of a supermarket in Philadelphia, Pennsylvania randomized to intervention (30) and wait-listed control (28) groups.  The majority of participants were African American (95%) and women (81%; Table 1).  The average age of the primary shopper was 50.4 years. The average household size was 3.8 persons, with an average of 1.7 children living in the household. Annual income was $25 000 or less in 69% of households (eligibility was up to $60 000). Sixty-two percent of participants were enrolled in the Supplemental  Nutrition Assistance Program and 29% in the Supplemental Nutrition Program for Women,  Infants and Children (WIC).  Household and shopper characteristics were similar in the 2 study groups, with the exception that more control than intervention participants were enrolled in WIC (43% vs 17%). Most participants (95%) lived within 2 miles of the study supermarket. | The study aimed to assess the impact of a rewards-based incentive program on fruit and vegetable purchases by low-income families including four phases:  1. baseline (8 weeks; no rewards),  2. intervention (8 weeks; rewards = 50% of  produce spending),  3. tapering (4 weeks; rewards = 25% of produce  spending), and  4. follow-up (6 weeks; no rewards)  Gift cards were used for the study supermarket to provide the incentives to participants.  The cards were given to participants at the beginning of the study without any dollar amount added. During the (full active) intervention phase, study shoppers received a rebate of 50% of the dollar amount they spent on fresh or frozen produce. In the tapering phase, the amount fell to 25%. During the follow-up phase, there was no price incentive. Earned rewards were added to the store gift card at 2-week intervals. A total of 4 study specific newsletters containing nutritional information and recipes involving fruit and vegetables were sent to study households. During phases 2 and 3, written reports of their produce purchases and the dollar amount accrued on the gift card with the newsletters were included. One week before starting the intervention phase, the participants received a mailing with study materials including:  - Two plastic magnetic stripe cards: 1 store loyalty card (branded for the study), with instructions for use, and 1 store gift card (without any dollar amount added);  - A study calendar with the relevant dates of each phase;  - A user-friendly explanation about how the rewards worked in each phase;  - A study newsletter; and  - The name and phone number to call for questions about the study.  The primary outcomes were the number of servings of fresh and frozen fruit and of fresh and frozen vegetables purchased per week.  Participants completed 2 surveys; 1 at the time of enrolment and 1 during follow-up to elicit participant experience with the study and point-of- sale purchase data were collected to gather purchase information related to fruit and vegetables. | Intervention households purchased 10.4 (95% CI = 4.8, 17.8; P < .002) more servings of fruit and vegetables than control households. After adjustment for weekly changes in produce prices, the difference between average purchases in intervention and control households was 10.2 servings (95% CI = 3.6, 25.7; P < .001).  Half of the intervention households purchased more than 8.4 servings of vegetables per week; the median number of vegetable servings purchased by the intervention group was more than 6 times that of the control group.  On average, intervention households purchased 8 (95% CI = 4.1, 13.4; P < .002) more vegetable servings than did control households and this increase remained with adjustment for weekly changes in vegetable prices.  The median weekly number of fruit servings purchased by the intervention group was more than 3 times that of control households.  On average, intervention households purchased 2.4 (95% CI = –0.2, 4.6; P = .06) more fruit servings per week than did control households. However, in a price-adjusted, as-randomized analysis, the difference was 2.5 servings (95% CI = 0.3, 9.5; P = .01). Study households that received a 50% rebate (intervention group) purchased both more vegetables and more fruit than did control households in price-adjusted randomized comparisons. |
| Reger (1999) [[24](#_ENREF_24)] | CBA, 14 supermarkets in two rural communities in West Virginia, USA of 35,000 and 34,000 residents respectively. The communities are separated by 110 miles and had distinct media markets. The newspapers, television and radio stations from each city did not reach residents of the other city. 366 and 374 participated in a pre and post telephone survey, respectively. Participants were 49.5 and 51.5y, 67% and 66% female, most (88% and 85%) had 12 years or more of education and 28% and 29% had a household income < $15,000 in intervention and control communities, respectively. | - Control community outside the media market -  - Intervention for 6 weeks: Message campaign including paid advertisements and public events produced to garner free media coverage in local news.  The intervention used paid advertising (radio, print media and television) and public relations (generated news coverage, press conferences, taste-testing events, expert advisory committee formed) to encourage members of the intervention community to switch from whole or 2% milk to 1% or fat-free milk.  Pre and post intervention telephone surveys were conducted during the month immediately before (n=366; n=374 randomly selected in intervention and comparison cities) and the month immediately after the campaign (73% of baseline). Milk sales data from 14 out of 16 supermarkets in two communities were collected. | In the intervention community low-fat milk increased from 29% of overall milk sales before the campaign to 46% of sales in the month after the campaign and to 42% at the 6-month follow-up. No significant variation was observed in sales of low fat milk in comparison supermarkets.  There was a decrease in the high-fat milk sales in the intervention community from 71% before the campaign to 54% and 58% in the months immediately after and 6 months after the campaign, respectively. Volume sales of high-fat milk decreased significantly in the intervention community from an average of 8135 gallons per supermarket per month before the campaign to 6224 and 6156 gallons in the month after and 6 months after the campaign (F = 9.29, p < 0.03). Across the same periods, high-fat milk sales in the comparison community were 78%, 79%, and 77% of overall milk sales and averaged 7271, 7731 and 6127 gallons per supermarket per month for the months before, after, and 6 months after the campaign, respectively (F = 2.88, p = 0.102). Telephone surveys supported sales data: 34.1% of high-fat milk drinkers in intervention city switched to low fat milk compared to 3.6% in comparison city (z=13.1, p<0.0001). This difference was mostly amongst those who were already drinking 2% fat milk rather than full-fat milk drinkers. |
| Reger (2000) [[25](#_ENREF_25)] | CBA, 21 supermarkets in three rural communities in West Virginia and USA, Parkersburg with 34,000 people, Beckley with 18,000 people and Martinsburg with 14,000 people. The communities were separated by 135 miles and had distinct media markets (the newspapers, television and radio stations from each city did not reach residents of the other city. Across the three communities, 400 customer participants were from 48.5 to 50.3y, from 84% to 93% were white, from 59% to 71% were female, from 22% to 24% had a college degree, from 46% to 59% were employed and from 20% to 30% had household income < $15,000. | Aim of study was to examine the effectiveness of various educational approaches for communicating the “1% of less” milk message.  The treatments included:   - Control community outside the media market (Martinsburg) - Message campaign including paid advertisements (Beckley) for 6 weeks. (radio and television). Cost $50,000 - Public events and educational activities for 8 weeks (Parkersburg). A full-time health educator trained 150 community volunteers to conduct educational activities in different public settings; supermarkets displayed signage; schools conducted educational activities and taste-testing; health presentations and worksite campaigns. Cost $51,000. Health professionals gave presentations to 1100 people   1300 people participated in milk taste tests  Pre and post intervention telephone surveys were conducted during the month immediately before and the month immediately after the campaign (n=400 in each community).  Supermarket sales data on milk sales were collected (month before, month after and 6 mo post intervention – 33% dropout rate). | The differences in low-fat milk supermarket sales as a proportion of overall milk sales (%) after the campaign compared to baseline, immediately after intervention and 6 mo [post-intervention] were not statistically significant compared with sales in the comparison city.  Before After Post  Control 23 22 21  Media 28 34 27  Education 23 28 29  Both intervention cities showed a higher proportion of high-fat milk drinkers drinking low-fat milk post intervention (19.6% and 12.8%) compared to control (6.8%). The intervention effect of switching from high-fat to low-fat was stronger for Parkensburg compared to media only city (z=2.21, p<0.05). |
| Rodgers (1994) [[26](#_ENREF_26)] | CBA, 40 supermarkets from a major food retailing outlet in Washington and Baltimore metropolitan areas, USA. Intervention and control groups were matched on size and socio-economic characteristics and presence or not of a pharmacy. | Control without any intervention  Intervention: 2-year multi-component supermarket intervention aimed to stimulate changes in knowledge and food purchasing behaviour consistent with dietary recommendations (increasing consumption of F&V and other fibre-containing foods) for cancer risk reduction and included:   - Special shelf price labels - A good guide containing calorie, fat, cholesterol, sodium, and fibre values for all items carrying the special shelf price labels - A monthly bulletin containing nutrition information and recipes - Signs in the produce department - Intensive multimedia advertising campaign   Program monitoring included:  - 9 visits to the 20 intervention stores to monitor the program,  - 3 telephone consumer surveys (at baseline, during 3 mo of the first year of the intervention and during 3 mo of the second year of the intervention) using a shortened version of the food frequency portion of the National Cancer Institute Health Habits and History Questionnaire and  - an analysis of sales data of selected foods collected each week for 1 baseline year and 2 intervention years. Eight food categories (cereal, baked goods, fresh produce, frozen vegetables, canned vegetables, canned and frozen beans, and dried fruit) were analysed. Foods in each of these groups were subcategorised as recommended (>2g fibre and <30% of calories from fat) and “other than recommended”. Outcome measures for sales data were: sales in ounces of recommended food items in each category and percent recommended in each category.  Evaluation aimed to assess: i) stores’ ability to implement the strategy; ii) change in consumer purchases of fiber-rich and lower-fat foods; iii) change in knowledge about diet and health; iv) self-reported food purchasing and preparation. | Positive intervention effect reported for an increased awareness of link between diet and health and awareness of the Eat for health program. No intervention effect shown for other measures, ie. i) self-reported purchasing and consumption of foods high in fibre (with the exception of dried beans) and low in fat; ii) change in food preparation methods; iii) increase in food knowledge with regards to cancer risk. There was a greater decrease in sales of recommended cereals in intervention stores than in control stores but according to Patterson (1992) this difference was not significant and the 9% adjusted decrease in sales of recommended cereals in intervention relative to control, in face of similar changes in market share, could be explained by greater relative decrease in the sales of all dry cereals in the intervention stores. The amount of recommended breads sold was higher during intervention compared to baseline in both control and intervention. Out of eight food categories assessed one food category showed a modest relative increase in the proportion of the recommended foods (fresh produce compared to all fresh produce sales) in the intervention stores. There was a smaller decline in the sales of recommended frozen vegetables in the intervention stores compared to control stores. The amount of recommended canned vegetables, dried beans, dried fruits and meat fell more in the control than in the intervention stores. The amount of recommended poultry increased more in the intervention than in the control stores. |
| Silzer (1994) [[27](#_ENREF_27)] | CBA with a non-equivalent comparison group design. One supermarket in Hamilton, Ontario, Canada. 124 women participated in the study. Most participants were between 30 and 65y, 70% were younger than 50y and around 35% had completed post-secondary education. | - Intervention: Telephone survey including demographic characteristics with adults who registered for supermarket tours one week before the tour.  - Control: A comparison group was formed using female volunteers from organizations requesting nutrition presentation from health department.  Supermarket tours (2h long, with 10-12 participants) aimed to increase understanding of food labels and to identify changes in their food purchasing and meal preparation practices leading to a decreased intake of fat and salt and increased of dietary fibre.  Questionnaire (16 questions) at baseline (completed at the supermarket before the tour for intervention group or mailed to comparison group at the same time) and at post-test was mailed or hand-delivered on the month following the tours to measure food labelling reading, food purchasing and food preparation behaviours. An entry form for a draw to win a cookbook was provided as an incentive. | The post-tests were completed by all comparison participants, but only 49 of the 61 tour participants. Four comparison participants did not complete a minimum of 11 out 13 questions and were excluded from the analysis.  There were no differences in demographic characteristics or in pre-test skill/behaviour scores (t = 1.89, p = 0.063). Program group had a greater gain in skill/behaviour one month following supermarket tour (0.22 (0.21)) than comparison group (0.21 (0.13), p < 0.001). There was no change in the mean score for the group that did not receive the intervention (from 0.49(0.19) to 0.48 (0.20)).  Reading labelling more often (68%), purchasing food lower in fat (40%), a decrease in the amount of fat in food preparation (43%) and purchasing food higher in fibre (36%) were the most frequently reported changes. |
| Song et al. (2009) [[28](#_ENREF_28)] | CBA, 9 intervention food stores (2 supermarkets and 7 corner stores in East Baltimore and 8 stores (2 supermarkets and 6 corner stores) in West Baltimore, 2 of the poorest areas of Baltimore with an annual median household income of $US 17000 and where the main population is Korean-American (85%-90%). The estimated number of Korean-American merchants ranges from 1000 to 2000 and of these, 750 Korean American merchants are Association members and about 70% are engaged in grocery stores or food service areas. | Ten healthy foods (based on standard criteria eg. low-sugar (<10g/serving) or high fibre cereals (>10% of the daily value per serving) identified to contribute most energy, fat and sugar intakes, selected from 24h recall, were promoted.  The intervention, aimed at changing practice of store owners, was implemented over 10 months. They were asked to stock the promoted foods and to display in-store intervention materials for customers.  Control: no intervention  Intervention: included:   - small monetary incentive given to storeowners per intervention phase to cover initial stocking costs (USD 25-50 per intervention phase). - Cultural guidelines to assist storeowners build better relationships with community members including practical suggestions based on formative research in the community - Nutrition education training (45-60min session) and booklet to improve nutrition-related knowledge of corner storeowners and ensure longer sustainability of the program. - Guidelines with practical suggestions about food purchasing, stocking and placing/display for easy access. - In-store intervention materials (posters, educational displays, flyers and shelf labels) and in-store promotions such as taste testings, incentives and giveaways   Data was collected through:   - a store impact questionnaire at baseline and post-intervention including physical store characteristics and storeowners’ psychosocial factors including food-related knowledge, self-efficacy for healthy food stocking, outcome expectations for food sales and programme effect. Psychosocial scores included outcome expectations of food sales (for 23 items), program promotion (for 5 items), self-efficacy (based on 23 statements about healthy food stocking) and food knowledge (9 questions) - weekly food sales records in corner stores to assess promoted food stocking and sales of key promoted foods, and - unstructured interviews with storeowners about operation of corner stores.   A stocking score was created by adding one point for each type of promoted food in stock at baseline, post-phase and post-intervention, respectively. A sales score was calculated by adding one point for each type of promoted healthy food if at least one unit was sold at baseline, post-phase and post-intervention. An overall healthy food stocking and sales score was calculated to show the change in food stocking and sales for each store. From 0 to 2 points were assigned to each promoted food, based on its stocking and sales at baseline and post-intervention. The highest score 2, was given to a promoted healthy food when the food was available at post-intervention and sales of the promoted food increased from baseline to post-intervention. | Supermarkets were included only for the analysis of psychosocial variables. Stocking healthy foods was not a major issue at supermarkets compared with corner stores since supermarkets already stocked a wide variety of healthy foods. Also, the promoted food sales were reported only for corner stores because participating supermarkets later proved reluctant to release their sales data. Due to the lack of a computerized sales tracking system, food sales were determined from store owners’ recall using weekly food sales records. Overall, there were no changes from baseline to post-intervention in scales created to assess storeowners’ outcome expectations for healthy food sales or programme effects, self-efficacy or knowledge scores between intervention and comparison corner stores. However, significant changes were observed for some specific foods. Outcome expectations for low-salt cracker sales decreased significantly in comparison stores. Self-efficacy scores for stocking promoted healthy foods such as low-sugar cereals and low-fat salad dressing increased significantly in intervention stores, but decreased in comparison stores. While corner stores in the comparison area had no or little change, significantly more corner stores in the intervention area showed increased stocking of some healthy foods at post-intervention. The stocking of baked/low-fat chips, low-salt crackers, cooking spray and whole wheat breads was sustained well even six months after the programme.  The average sales scores for all promoted foods combined were significantly higher for intervention than control stores at both post-phase (following promotional activities) and post-intervention |
| Sturm 2013 [[29](#_ENREF_29)] | Controlled before and after study including participants in the HealthyFood program in over 400 designated supermarkets across all provinces in South Africa.  About 60% of the Vitality households activated the HealthyFood benefıt and initially received a 10% rebate on healthy food purchases;  67% of the households also completed the health risk assessment and became eligible for the 25% rebate. The remaining 40% did not receive a rebate on healthy food purchases. Purchases were collapsed into monthly observations, resulting in a total of 1,909,740 observations (household months). Households who became eligible for a rebate during the study period already had a larger proportion of overall food expenditure going toward healthy foods and a smaller proportion toward less desirable foods at baseline (when nobody was eligible for the rebate) than households that never participated in the program. Participants also lived closer to a Pick n Pay supermarket than to a competing supermarket; the opposite was true for nonparticipants. The difference between nonparticipants and participants is signifıcant for all variables. | Following activation online or by phone, members immediately receive a 10% rebate for healthy foods, which increases to a 25% rebate on completion of an online health risk assessment questionnaire.  The rebate is capped at a maximum monthly purchase amount of approximately $480 per household and a limit related to participation in health promotion activities.  A complete list of eligible items (more than 6000) can be found on Discovery’s website and is also distributed as brochures to program participants. Participating supermarkets have in-store signs identifying eligible foods; they are also marked on the store receipt. The labelling was implemented prior to the study period and was not changed during the study period. The panel identifıed less-desirable foods and beverages as those that are high in saturated fats, trans-fatty acids, added sugar, salt, or refıned starch. The less-desirable food group includes sweets, chocolates, ice cream, sugary foods, chips, sugar-sweetened beverages, and fried items. All foods not specifıcally classifıed as either healthy or less desirable were considered neutral and were neither encouraged nor discouraged. | Among program participants, the ratio of fruit/vegetables and healthy foods to total food expenditure increased and the ratio of less-desirable food to total food expenditure decreased substantially after participants became eligible for a rebate. No similar increase/decrease was found among the matched ineligible households. The change appeared immediately after participants became eligible, and there was neither a convergence back to baseline levels nor an increasing effect over time. For healthy food purchases, there was an increase a few months before the actual rebate eligibility, suggesting some type of anticipation effect. The results from household fıxed-effect regression models show the ratios of healthy food purchases and of fruit/vegetables to total food purchases increases, whereas the ratio of less-desirable food purchases declines. All estimated coeffıcients are signifıcant at *p*<0.001. The increase in healthy food purchases is larger than the decline in less desirable food purchases, which means that some of that shift is due to a decline in the ratio of neutral foods. Rebates of 10% and 25% for healthy foods were associated with an increase in the ratio of expenditure on healthy foods to total food expenditure by 6.0% (95% CI=5.3, 6.8) and 9.3% (95% CI=8.5, 10.0); an increase in the ratio of expenditure on fruit and vegetables to total food expenditure by 5.7% (95% CI=4.5, 6.9) and 8.5% (95% CI=7.3, 9.7); and a decrease in the ratio of expenditure on less-desirable to total food expenditure by 5.6% (95% CI=4.7, 6.5) and 7.2% (95% CI=6.3, 8.1). |
| Waterlander 2013[[30](#_ENREF_30)] | RCT including 199 participants randomized to 4 conditions: 50% discount on F&V, nutrition education (NE), 50% price discount + NE and control. The nutrition education was provided outside the store and therefore only the price discount treatment is discussed in this review. A sample of 173 was included in analysis. Most participants were female (96%), and around 80% purchased at least half of their F&Vs in the participating supermarket. | The pricing intervention consisted of a 50% price discount on F&Vs provided by coupons. Participants were sent discount coupons (by mail) for 7 types of vegetables and 5 types of fruit every 2 wk with 2 coupons for each item and with changing discount selections.  To prevent sharing, each coupon had a maximum amount of produce that could be purchased. Discounted products were mostly fresh produce, but every series also included some canned and frozen products. Discounts were given on F&Vs as a whole product only and not on products such as salads or ready-to-eat meals. Fruit juices, vegetable juices, apple sauce, tomato sauce, and potatoes were also excluded from the discount.  The main outcome measures were household F&V purchases (combined, measured in grams) and household expenditures on other supermarket based on supermarket cash receipts (from the participating supermarkets) that were collected at each measurement round during a 2-wk period.  To capture purchases outside the participating supermarkets, participants were additionally asked what proportion of F&Vs was purchased at the participating supermarket. Second, the supermarket owners provided information on the use of discount coupons in each period. Finally, F&V consumption was measured by a shortened food-frequency questionnaire (FFQ), to measure F&V consumption to detect participants who consumed sufficient (>400 g/d) or insufficient F&Vs.  Participants received several small gifts to prevent dropout including a substantial discount on F&Vs for participants in the discount group; small gifts sent to the whole sample, including fridge magnets, tokens for shopping carts, shopping bags, ballpoint pens, and flower seeds; and a St Nicholas gift and a Christmas card. At the end of the study, a special meeting was arranged at all 4 supermarkets where participants received a box filled with groceries and a gift coupon (nondiscount groups) or a discount coupon only (discount groups). | Participants in the discount group purchased more F&Vs compared with the control group; however, this was significant only at 6 mo. The largest part of the difference in effects found between the crude and adjusted models was explained by the proportion of purchases that were made in the participating supermarkets. At 9 mo, 3 mo after the intervention stopped, the differences between the groups had disappeared.  The percentage of participants who consumed sufficient amounts of F&Vs increased significantly from 42.5% at baseline to 61.3% at 6 mo in the discount groups (P = 0.03). |
| Winett (1991) [[31](#_ENREF_31)] | RCT in a 40,000-square-foot store owned by a large supermarket chain in Virginia, USA. 77 participated in the baseline but only 69 were included in the follow-up analysis, 80% were female, had a median income of $35000 and had a mean age of 40y. 50% of the participants had completed 4 years of college. | The project consisted of a 7-week baseline period, 7 to 8-week intervention period and 3-week follow-up. Participants completed paper-and-pencil forms, entered their intended food purchases into the Nutrition for a Lifetime System (NLS) and sent in their food receipts of actual purchases.  77 participants took part in the baseline and intervention phase, 8 dropped-out and 69 were followed-up. Participants were randomised to:   - Control condition without any intervention - NLS-1 including a custom-designated kiosk, a computer, software, video content in the form of 6 weekly programs, and a printed feedback-on-intended-purchase function. - NLS-2 had a few changes based on consumer’s response including more specific information via additional short video segments and more specific feedback and goal-setting information. There were 5 programs.   For the analysis, NLS-1 and NLS-2 were combined.  Measures of a participant’s intended and actual purchases were collected weekly using a checklist with 230 major food items further categorised into 13 categories for analysis.  Additional data derived from both intended and actual food purchase data included dollars spent per week on food purchases and estimates of percentage calories from fat and grams of fibre per day household member. | During the intervention phase, significant differences favouring the experimental participants were found for the high-fat meat category (lower amount, F = 14.87, p < 0.001; lower frequency (F = 17.60, p < 0.001), the high-fibre grains/cereals category (greater amount, F = 11.95, p < 0.001); greater frequency, F = 10.36, p < 0.01), and high-fat dairy category (lower amount, F = 4.53, p < 0.05).  During the follow-up phase, significant differences favouring the experimental participants were found for high-fibre grains/cereals (greater amount, F = 5.05, p < 0.05), low-fat dairy (greater amount, F = 5.16, p < 0.05) and high fat dairy (lower amount, F = 6.13, p < 0.05); lower frequency, F = 6.32, p < 0.05). There were no other significant differences between groups during intervention or follow-up for any other food category. Compared to the control group, substantial changes were made by the experimental group including decreased high-fat meat by 37% and 31%, increased high-fibre grains/cereals by 62% and 25%, and decreased high-fat dairy by 20% and 10%, in amount and frequency, respectively. However the shift in high-fat meat by experimental participants was not maintained during follow-up phase. Individual participant data analysis showed that during the intervention phase, the percentage of experimental participants decreasing their amount of purchases in the high-fat meat category (57.5%) and the high fat dairy category (67.5%) was about 25% greater than the percentage of control participants (32.5%; 43.2%) decreasing their amount of purchase compared to the baseline phase. For the high-fibre grains/cereal category, 77.7% of the experimental participants increased purchases compared to 51.3% of control participants. These differences were maintained in the follow-up phase. |
| Winett (1991) [[32](#_ENREF_32)] | RCT in a 40,000-square-foot store owned by a large supermarket chain in Virginia, USA, open 24 hours a day. 61 participated in the study, 80% were female, had a median income of $35000, had a mean age of 40y and 50% had completed college and had a median income of $35000. | The project consisted of a 7-week baseline period, 7 to 8-week intervention period and 3-week follow-up. Participants completed paper-and-pencil forms, entered their intended food purchases into the Nutrition for a Lifetime System (NLS) and sent in their food receipts of actual purchases.  Participants were assigned to:   - Intervention including a custom-designated kiosk, a computer, software, video content in the form of 6 weekly programs, and a printed feedback-on-intended-purchase function. - Control condition without any intervention   Measures of a participant’s intended and actual purchases were weekly collected. Major food items were further categorised into 13 categories according to food type and nutritional content.  Calories from fat and daily per capita consumption of fibre were derived from intended and actual food purchase.  Outcome measures were: % calories from fat; daily per capita consumption of fibre; and, cost. | 61 participants were enrolled but only 49 returned at least 3 shopping receipts and had data analysed at the intervention phase and 42 at the follow-up (who had at least 2 receipts).  Compared to control participants, experimental participants purchased more low-fat dairy products during follow-up (3679.9 vs 2841.6, p < 0.05). Positive changes were also observed for the high-fat meat category during intervention, with lower purchase in intervention group (789.2) compared to control group (1360.5) but not during follow-up (830.3 for intervention vs 1026.2 for control; p>0.05). Percentage calories from fat intended and actual was lower in experimental participants (34.3%) compared to control participants (39.5%) but this effect was no longer significant at follow-up (34.6%). |
| Winett (1997) [[4](#_ENREF_4)] | RCT, 2 small supermarkets in rural towns in south western Virginia. 127 shopper participants were predominantly white (95%), households with a female (86%) primary shopper who had a median age of 40 years. Mean household income was $30000 annually with over one quarter (28%) of the sample with incomes under $20000. Household size ranged from 1 to 8 members. | Participants were recruited in 2 small supermarkets by brief face-to-face contact. Interested shoppers received information about the project, completed a screening about their supermarket food purchases and took enrolment information with them. Participants completed initial assessment of nutrition knowledge and participant characteristics at home and returned them in postage-paid envelopes. Participants returned all supermarket receipts with annotated size and type of products.  Participants were randomly assigned to:   - no-treatment control condition (n=51) - intervention: using the system kiosk in the supermarket (n=54) during intervention and maintenance   Intervention consisting of   - access to kiosk to watch 10 content and 4 maintenance segments introduced weekly focused on increasing purchases and consumption of cruciferous vegetables, fruits, high-fibre cereals, low fat dairy and lean protein sources as well as on decreasing consumption of fat from butter, beef and snacks. Segment also suggested strategies for monitoring and planning food purchases and meal preparation, and provided opportunities for personalized goal setting and feedback for each targeted food group or behaviour change strategy. - Target food coupons valued $0.50 to $1.00 could be selected in each segment and redemption was limited to the participants at the kiosk store within 1 week of printing. Completion of all parts of each segment required 5 min.   Data from the 78,000 individual food items generated percent calories from fat, fibre grams per 100 calories, and F&V servings per 1000 calories purchased for each participant.  A nutrition knowledge questionnaire containing 95 items and a participant enrolment questionnaire measuring demographic characteristics were applied.  Outcome measures were: i) % of fat in purchased food calories; ii) gms of fibre and no. serves of fruit and vegetables purchased; iii) purchases of NLS users redeeming coupons. | Participants in the intervention decreased fat levels, increased fibre content, and increased serving of F&V in their post-treatment in supermarket purchases compared to control.  Changes in fat and fibre levels were maintained at follow-up.  Increases in F&V for both groups at follow-up reflected seasonal trends. Percentage of calories from fat was lower in the intervention group compared to control group when controlling for confounders.  Participants with higher baseline nutrition knowledge achieved lower levels of fat in post-intervention.  Smaller families and those with higher SES had higher overall post-test fibre and fibre from F&V purchased. Attainment of minimum recommended serving of F&V at post-test was more likely for kiosk users, participants with higher SES, and smaller household size.  53 participants completed maintenance weeks. Follow-up resulted in increases in F&V with associated increase in produce fibre and decreases in overall calories from fat.  Decreases in modified total calories from fat (χ2=3.12, p=0.08), fat levels in dairy foods (χ2=4.27, p=0.04) and increases in total fibre (χ2=12.96, p=0.002) were greater among participants redeeming greater amounts of program coupons. |

**References:**

1. Achabal DD, McIntyre SH, Bell CH, Tucker N: **The Effect of Nutrition P-O-P Signs on Consumer Attitudes and Behavior**. *Journal of Retailing* 1987, **63**(1):9.

2. Anderson ES, Winett RA, Wojcik JR, Winett SG, Bowden T: **A computerized social cognitive intervention for nutrition behavior: Direct and mediated effects on fat, fiber, fruits, and vegetables, self-efficacy, and outcome expectations among food shoppers**. *Annals of Behavioral Medicine* 2001, **23**(2):88-100.

3. Anderson ES, Winett RA, Bickley PG, Walberg-Rankin J, Moore JF, Leahy M, Harris CE, Gerkin RE: **The Effects of a Multimedia System in Supermarkets To Alter Shoppers' Food Purchases: Nutritional Outcomes and Caveats**. *Journal of Health Psychology* 1997, **2**(2):209-223.

4. Winett RA, Anderson ES, Bickley PG, Walberg-Rankin J, Moore JF, Leahy M, Harris CE, Gerkin RE: **Nutrition for a Lifetime System©: A multimedia system for altering food supermarket shoppers' purchases to meet nutritional guidelines**. *Computers in Human Behavior* 1997, **13**(3):371-392.

5. Ayala GX, Baquero B, Laraia BA, Ji M, Linnan L: **Efficacy of a store-based environmental change intervention compared with a delayed treatment control condition on store customers' intake of fruits and vegetables**. *Public Health Nutr* 2013, **16**(11):1953-1960.

6. Bergen D, Yeh MC: **Effects of energy-content labels and motivational posters on sales of sugar-sweetened beverages: Stimulating sales of diet drinks among adults study**. *Journal of the American Dietetic Association* 2006, **106**(11):1866-1869.

7. Booth-Butterfield S, Reger B: **The message changes belief and the rest is theory: the "1% or less" milk campaign and reasoned action**. *Preventive Medicine* 2004, **39**(3):581-588.

8. Connell D, Goldberg JP, Folta SC: **An intervention to increase fruit and vegetable consumption using audio communications: In-store public service announcements and audiotapes**. *Journal of Health Communication* 2001, **6**(1):31-43.

9. Ernst ND, Wu M, Frommer P, Katz E, Matthews O, Moskowitz J, Pinsky JL, Pohl S, Schreiber GB, Sondik E *et al*: **Nutrition education at the point of purchase: the foods for health project evaluated**. *Prev Med* 1986, **15**(1):60-73.

10. Fiske A, Cullen KW: **Effects of promotional materials on vending sales of low-fat items in teachers' lounges**. *Journal of the American Dietetic Association* 2004, **104**(1):90-93.

11. Foster GD, Karpyn A, Wojtanowski AC, Davis E, Weiss S, Brensinger C, Tierney A, Guo W, Brown J, Spross C *et al*: **Placement and promotion strategies to increase sales of healthier products in supermarkets in low-income, ethnically diverse neighborhoods: a randomized controlled trial**. *The American journal of clinical nutrition* 2014.

12. French SA, Jeffery RW, Story M, Breitlow KK, Baxter JS, Hannan P, Snyder MP: **Pricing and promotion effects on low-fat vending snack purchases: The CHIPS study**. *American Journal of Public Health* 2001, **91**(1):112-117.

13. Gittelsohn J, Vijayadeva V, Davison N, Ramirez V, Cheung LWK, Murphy S, Novotny R: **A Food Store Intervention Trial Improves Caregiver Psychosocial Factors and Children's Dietary Intake in Hawaii**. *Obesity* 2010, **18**:S84-S90.

14. Gittelsohn J, Song HJ, Suratkar S, Kumar MB, Henry EG, Sharma S, Mattingly M, Anliker JA: **An urban food store intervention positively affects food-related psychosocial variables and food behaviors**. *Health education & behavior : the official publication of the Society for Public Health Education* 2010, **37**(3):390-402.

15. Herman DR, Harrison GG, Afifi AA, Jenks E: **Effect of a targeted subsidy on intake of fruits and vegetables among low-income women in the special supplemental nutrition program for women, infants, and children**. *American Journal of Public Health* 2008, **98**(1):98-105.

16. Huang A, Barzi F, Huxley R, Denyer G, Rohrlach B, Jayne K, Neal B: **The effects on saturated fat purchases of providing internet shoppers with purchase- specific dietary advice: a randomised trial**. *PLoS clinical trials* 2006, **1**(5):e22.

17. Jeffery RW, Pirie PL, Rosenthal BS, Gerber WM, Murray DM: **Nutrition education in supermarkets: an unsuccessful attempt to influence knowledge and product sales**. *J Behav Med* 1982, **5**(2):189-200.

18. Kocken PL, Eeuwijk J, Kesteren NMCV, Dusseldorp E, Buijs G, Bassa-Dafesh Z, Snel J: **Promoting the Purchase of Low-Calorie Foods From School Vending Machines: A Cluster-Randomized Controlled Study**. *Journal of School Health* 2012, **82**(3):115-122.

19. Kristal AR, Goldenhar L, Muldoon J, Morton RF: **Evaluation of a supermarket intervention to increase consumption of fruits and vegetables**. *American Journal of Health Promotion* 1997, **11**(6):422-425.

20. Levy AS, Matthews O, Stephenson M, Tenney JE, Schucker RE: **The Impact of a Nutrition Information Program on Food Purchases**. *Journal of Public Policy & Marketing* 1985, **4**(1):1-13.

21. Milliron BJ, Woolf K, Appelhans BM: **A point-of-purchase intervention featuring in-person supermarket education affects healthful food purchases**. *J Nutr Educ Behav* 2012, **44**(3):225-232.

22. Ni Mhurchu C, Blakely T, Jiang YN, Eyles HC, Rodgers A: **Effects of price discounts and tailored nutrition education on supermarket purchases: a randomized controlled trial**. *American Journal of Clinical Nutrition* 2010, **91**(3):736-747.

23. Phipps EJ, Braitman LE, Stites SD, Singletary SB, Wallace SL, Hunt L, Axelrod S, Glanz K, Uplinger N: **Impact of a Rewards-Based Incentive Program on Promoting Fruit and Vegetable Purchases**. *Am J Public Health* 2014.

24. Reger B, Wootan MG, Booth-Butterfield S: **Using mass media to promote healthy eating: A community-based demonstration project**. *Preventive Medicine* 1999, **29**(5):414-421.

25. Reger B, Wootan MG, Booth-Butterfield S: **A comparison of different approaches to promote community-wide dietary change**. *American Journal of Preventive Medicine* 2000, **18**(4):271-275.

26. Rodgers AB, Kessler LG, Portnoy B, Potosky AL, Patterson B, Tenney J, Thompson FE, Krebs-Smith SM, Breen N, Mathews O *et al*: **"Eat for Health": A Supermarket Intervention for Nutrition and Cancer Risk Reduction**. *American Journal of Public Health* 1994, **84**(1):72-76.

27. Silzer JS, Sheeshka J, Tomasik HH, Woolcott DM: **AN EVALUATION OF SUPERMARKET SAFARI NUTRITION EDUCATION TOURS**. *Journal of the Canadian Dietetic Association-Revue De L Association Canadienne Des Dietetistes* 1994, **55**(4):179-183.

28. Song HJ, Gittelsohn J, Kim M, Suratkar S, Sharma S, Anliker J: **A corner store intervention in a low-income urban community is associated with increased availability and sales of some healthy foods**. *Public Health Nutrition* 2009, **12**(11):2060-2067.

29. Sturm R, An R, Segal D, Patel D: **A cash-back rebate program for healthy food purchases in South Africa: results from scanner data**. *Am J Prev Med* 2013, **44**(6):567-572.

30. Waterlander WE, de Boer MR, Schuit AJ, Seidell JC, Steenhuis IH: **Price discounts significantly enhance fruit and vegetable purchases when combined with nutrition education: a randomized controlled supermarket trial**. *The American journal of clinical nutrition* 2013, **97**(4):886-895.

31. Winett RA, Moore JF, Wagner JL, Hite LA, Leahy M, Neubauer TE, Walberg JL, Walker WB, Lombard D, Geller ES *et al*: **Altering shoppers' supermarket purchases to fit nutritional guidelines: an interactive information system**. *Journal of applied behavior analysis* 1991, **24**(1):95-105.

32. Winett RA, Wagner JL, Moore JF, Walker WB, Hite LA, Leahy M, Neubauer T, Arbour D, Walberg J, Geller ES *et al*: **An experimental evaluation of a prototype public access nutrition information system for supermarkets**. *Health Psychology* 1991, **10**(1):75-78.
