# Supplementary material for: Nutrition interventions at point-of-sale to encourage healthier food purchasing: a systematic review
Source: BMC Public Health. 2014 Sep 5;14:919. doi: 10.1186/1471-2458-14-919 (PMC4180547; doi:10.1186/1471-2458-14-919)
Supplement: Supplementary file 4 — Additional file 3: Table S3: Interactive and non-interactive nutrition education activity type, frequency and duration by intervention type. (DOCX 70 KB) [file 12889_2014_7082_MOESM4_ESM.docx]

**Additional file 3: Table S3 – Interactive and non-interactive nutrition education activity type, frequency and duration by intervention type**

| **Reference** | **Non-interactive activities** | | | | | | | **Interactive activities** | | | | | |  |  |
| --- | --- | --- | --- | --- | --- | --- | --- | --- | --- | --- | --- | --- | --- | --- | --- |
|  | **Media ad** | **Public events** | **Posters and banners** | **Point-of-purchase signs** | **Shelf takers** | **Hands out (flyers, guide, brochure)** | **Recipes** | **Cooking demo** | **Tour** | **Taste testing** | **Nutrition education session** | **Duration** | |  |  |
| **Nutrition education and promotion alone** | | | | | | | | | | | | |  |  |  |
| Achabal (1987) [[1](#_ENREF_1)] |  |  |  | 4 wk |  | 4 wk |  |  |  |  |  | 4 wk | |  |  |
| Booth-Butterfield (2004) [[2](#_ENREF_2)] | 98 paid TV advertisements, 168 cable TV, 120 radio, 1 daily newspaper and 5 weekly newspaper | 5 media events |  |  |  |  |  |  |  | 2 on low fat milk |  | 6 wk | |  |  |
| Connell (2001) [[3](#_ENREF_3)] |  | 30 to 60 sec every 30 min |  |  |  |  |  |  |  |  | 2x1hr video programs | 4 wk | |  |  |
| Ernst (1986) [[4](#_ENREF_4)] | a series of media cues in newspapers and 60-sec radio spots and a press briefing to introduce the project. | One press briefing | 3000 | 2000 |  | 4-page brochures every 2 wks | Every 2 wk |  |  |  |  | 48 wk | |  |  |
| Foster (2014) [[5](#_ENREF_5)] |  |  |  | Each month |  |  |  |  |  | 1 / mo for 2 to 3 h each |  | 24 wk | |  |  |
| Jeffery (1982) [[6](#_ENREF_6)] |  |  | 6 posters changed at 4-wk intervals over 12 weeks, 2 posters for 6 wks each | 12 small signs with messages over 12 weeks |  | 2 brochures for 12 wks, 6 small message cards for 6 wks and 12 small tear-off pads for 6 wks | 6 small different recipe tear-off pads for 6 wks |  |  |  |  | 12 wk + 6 wk + 6 wk over 6 mo | |  |  |
| Levy (1985) [[7](#_ENREF_7)] | 300 radio spots mainly in the first 8 weeks |  |  |  | 400 products | One 25-page guide available over 2 years in the store explaining the guidelines for defining low or reduced dietary components, listing products and quantitative nutrient declarations by food category, and offering dietary hints. |  |  |  |  |  | 2y | |  |  |
| Milliron (2012) [[8](#_ENREF_8)] |  |  |  | NFI | 600 | shopping lists and a monthly newsletter | NFI |  |  |  | Brief (10 min), face to face nutrition education | 4 mo | |  |  |
| Ni Mhurchi et al. (2010)^1^ [[9](#_ENREF_9)] |  |  |  |  |  | Monthly for 6 mo |  |  |  |  | Tailored at each purchase | 24 wk |  |  |  |
| Reger (1999) [[10](#_ENREF_10)] | 98 paid TV advertisements, 168 cable TV, 120 radio, 1 daily newspaper and 5 weekly newspaper | 5 media events |  |  |  |  |  |  |  | 2 on low fat milk |  | 6 wk | |  |  |
| Reger (2000) [[11](#_ENREF_11)] | 98 paid TV advertisements, 168 cable TV, 120 radio, 1 daily newspaper and 5 weekly newspaper | 5 media events; 150 trained community people gave presentations to 1100 people |  |  |  |  |  |  |  | 1300 people taste tested milk |  | 6-8 wk | |  |  |
| Rodgers (1994) [[12](#_ENREF_12)] | multimedia |  |  | NFI | NFI | Monthly | Monthly |  |  |  |  | 2y | |  |  |
| Silzer (1994) [[13](#_ENREF_13)] |  |  |  |  |  |  |  |  | 1 2h |  |  | 2h | |  |  |
| Winett (1991) [[14](#_ENREF_14)] |  |  |  |  |  |  |  |  |  |  | Video weekly | 6 wk | |  |  |
| Winett (1991) [[15](#_ENREF_15)] |  |  |  |  |  |  |  |  |  |  | Video weekly | 7 wk | |  |  |
| **Nutrition education plus enhanced availability of healthy foods** | | | | | | | | | | | | | | | |
| Gittelsohn (2010a) [[16](#_ENREF_16)] |  |  | NFI |  | NFI | 4-6 | 4-6 | 2-3 |  | 2-3 |  | 4 themed phases, each of 6-8 wk |  |  |  |
| **Monetary incentive alone** | | | | | | | | | | | | | | | |
| Herman et al.(2008) [[17](#_ENREF_17)] |  |  |  |  |  |  |  |  |  |  |  | 6 mo |  |  |  |
| Ni Mhurchi et al. (2010) ^1^ [[9](#_ENREF_9)] |  |  |  |  |  |  |  |  |  |  |  | 24 wk |  |  |  |
| Waterlander [[18](#_ENREF_18)] |  |  |  |  |  |  |  |  |  |  |  | 6 mo |  |  |  |
| Sturm [[19](#_ENREF_19)] |  |  |  |  |  |  |  |  |  |  |  | 28 mo |  |  |  |
| **Nutrition education plus monetary incentive** | | | | | | | | | | | | | | |  |
| **To customers** |  |  |  |  |  |  |  |  |  |  |  |  |  |  |  |
| Ni Mhurchi et al. (2010) ^1^ [[9](#_ENREF_9)] |  |  |  |  |  | Monthly for 6 mo |  |  |  |  | Tailored at each purchase | 24 wk |  |  |  |
| Anderson (1997) [[20](#_ENREF_20)] |  |  |  |  |  |  |  |  |  |  | Video weekly | 15 wk | |  |  |
| Anderson (2001) [[21](#_ENREF_21)] |  |  |  |  |  |  |  |  |  |  | Video weekly | 15 wk |  |  |  |
| Kristal et al. (1997) [[22](#_ENREF_22)] |  |  |  | NFI **^2^** |  | Weekly 6mo and biweekly 6mo | N/A |  |  | Twice each month |  | 12 mo |  |  |  |
| Winett (1997) [[23](#_ENREF_23)] |  |  |  |  |  |  |  |  |  |  | Video weekly | 15 wk |  |  |  |
| Phipps [[24](#_ENREF_24)]^3^ |  |  |  |  |  |  |  |  |  |  |  | 8 wk |  |  |  |
| **To both customers and store owners** | | | | | | | | | | | | |  |  |  |
| Song et al. (2009) [[25](#_ENREF_25)] |  |  | NFI |  | NFI | NFI |  |  |  | NFI |  | 5x2-mo themed phases over 10 mo |  |  |  |
| Gittelsohn (2010b) [[26](#_ENREF_26)] |  |  | NFI |  | NFI | NFI |  |  |  | Twice per mo |  | 5 themed phases, each lasting 2mo |  |  |  |
| Ayala [[27](#_ENREF_27)] | Store public announcement system describing the  journey of 8 fruit and vegetable characters |  | NFI | 8 wk | NFI | Weekly | Weekly | Weekly |  |  |  | 8 wk |  |  |  |
| **Vending machines** | | | | | | | | | | | | |  |  |  |
| Bergen (2006) [[28](#_ENREF_28)] |  |  | 5 wk |  | 5 wk | NFI |  |  |  |  |  | 5 wk |  |  |  |
| Fiske (2004) [[29](#_ENREF_29)] |  |  |  | 4 wk | 4 wk |  |  |  |  |  |  | 4 wk |  |  |  |
| French et al. (2001) [[30](#_ENREF_30)] |  |  |  | 4wk |  |  |  |  |  |  |  | 1 mo in each of the 24 sites over 12 mo |  |  |  |
| Kocken (2012) [[31](#_ENREF_31)] |  |  | NFI | 6 wk |  | NFI |  |  |  |  |  | 3 x 6-wk phases |  |  |  |
| **Online shopping** | | | | | | | | | | | | | | | |
| Huang (2006) [[32](#_ENREF_32)] |  |  |  |  |  |  |  |  |  |  | Advice tailored to the selected food items for purchase and opportunity to swap them | 5 months |  |  |  |

**^1^** Study mentioned three times because there are three treatments in addition to the control, and each of the treatments fit different intervention category. **^2^** NFI **=** No further information on frequency available. ^3^ Nutrition education was provided outside store but included feedback to customer on purchase data

**References**

1. Achabal DD, McIntyre SH, Bell CH, Tucker N: **The Effect of Nutrition P-O-P Signs on Consumer Attitudes and Behavior**. *Journal of Retailing* 1987, **63**(1):9.

2. Booth-Butterfield S, Reger B: **The message changes belief and the rest is theory: the "1% or less" milk campaign and reasoned action**. *Preventive Medicine* 2004, **39**(3):581-588.

3. Connell D, Goldberg JP, Folta SC: **An intervention to increase fruit and vegetable consumption using audio communications: In-store public service announcements and audiotapes**. *Journal of Health Communication* 2001, **6**(1):31-43.

4. Ernst ND, Wu M, Frommer P, Katz E, Matthews O, Moskowitz J, Pinsky JL, Pohl S, Schreiber GB, Sondik E *et al*: **Nutrition education at the point of purchase: the foods for health project evaluated**. *Prev Med* 1986, **15**(1):60-73.

5. Foster GD, Karpyn A, Wojtanowski AC, Davis E, Weiss S, Brensinger C, Tierney A, Guo W, Brown J, Spross C *et al*: **Placement and promotion strategies to increase sales of healthier products in supermarkets in low-income, ethnically diverse neighborhoods: a randomized controlled trial**. *The American journal of clinical nutrition* 2014.

6. Jeffery RW, Pirie PL, Rosenthal BS, Gerber WM, Murray DM: **Nutrition education in supermarkets: an unsuccessful attempt to influence knowledge and product sales**. *J Behav Med* 1982, **5**(2):189-200.

7. Levy AS, Matthews O, Stephenson M, Tenney JE, Schucker RE: **The Impact of a Nutrition Information Program on Food Purchases**. *Journal of Public Policy & Marketing* 1985, **4**(1):1-13.

8. Milliron BJ, Woolf K, Appelhans BM: **A point-of-purchase intervention featuring in-person supermarket education affects healthful food purchases**. *J Nutr Educ Behav* 2012, **44**(3):225-232.

9. Ni Mhurchu C, Blakely T, Jiang YN, Eyles HC, Rodgers A: **Effects of price discounts and tailored nutrition education on supermarket purchases: a randomized controlled trial**. *American Journal of Clinical Nutrition* 2010, **91**(3):736-747.

10. Reger B, Wootan MG, Booth-Butterfield S: **Using mass media to promote healthy eating: A community-based demonstration project**. *Preventive Medicine* 1999, **29**(5):414-421.

11. Reger B, Wootan MG, Booth-Butterfield S: **A comparison of different approaches to promote community-wide dietary change**. *American Journal of Preventive Medicine* 2000, **18**(4):271-275.

12. Rodgers AB, Kessler LG, Portnoy B, Potosky AL, Patterson B, Tenney J, Thompson FE, Krebs-Smith SM, Breen N, Mathews O *et al*: **"Eat for Health": A Supermarket Intervention for Nutrition and Cancer Risk Reduction**. *American Journal of Public Health* 1994, **84**(1):72-76.

13. Silzer JS, Sheeshka J, Tomasik HH, Woolcott DM: **AN EVALUATION OF SUPERMARKET SAFARI NUTRITION EDUCATION TOURS**. *Journal of the Canadian Dietetic Association-Revue De L Association Canadienne Des Dietetistes* 1994, **55**(4):179-183.

14. Winett RA, Moore JF, Wagner JL, Hite LA, Leahy M, Neubauer TE, Walberg JL, Walker WB, Lombard D, Geller ES *et al*: **Altering shoppers' supermarket purchases to fit nutritional guidelines: an interactive information system**. *Journal of applied behavior analysis* 1991, **24**(1):95-105.

15. Winett RA, Wagner JL, Moore JF, Walker WB, Hite LA, Leahy M, Neubauer T, Arbour D, Walberg J, Geller ES *et al*: **An experimental evaluation of a prototype public access nutrition information system for supermarkets**. *Health Psychology* 1991, **10**(1):75-78.

16. Gittelsohn J, Vijayadeva V, Davison N, Ramirez V, Cheung LWK, Murphy S, Novotny R: **A Food Store Intervention Trial Improves Caregiver Psychosocial Factors and Children's Dietary Intake in Hawaii**. *Obesity* 2010, **18**:S84-S90.

17. Herman DR, Harrison GG, Afifi AA, Jenks E: **Effect of a targeted subsidy on intake of fruits and vegetables among low-income women in the special supplemental nutrition program for women, infants, and children**. *American Journal of Public Health* 2008, **98**(1):98-105.

18. Waterlander WE, de Boer MR, Schuit AJ, Seidell JC, Steenhuis IH: **Price discounts significantly enhance fruit and vegetable purchases when combined with nutrition education: a randomized controlled supermarket trial**. *The American journal of clinical nutrition* 2013, **97**(4):886-895.

19. Sturm R, An R, Segal D, Patel D: **A cash-back rebate program for healthy food purchases in South Africa: results from scanner data**. *Am J Prev Med* 2013, **44**(6):567-572.

20. Anderson ES, Winett RA, Bickley PG, Walberg-Rankin J, Moore JF, Leahy M, Harris CE, Gerkin RE: **The Effects of a Multimedia System in Supermarkets To Alter Shoppers' Food Purchases: Nutritional Outcomes and Caveats**. *Journal of Health Psychology* 1997, **2**(2):209-223.

21. Anderson ES, Winett RA, Wojcik JR, Winett SG, Bowden T: **A computerized social cognitive intervention for nutrition behavior: Direct and mediated effects on fat, fiber, fruits, and vegetables, self-efficacy, and outcome expectations among food shoppers**. *Annals of Behavioral Medicine* 2001, **23**(2):88-100.

22. Kristal AR, Goldenhar L, Muldoon J, Morton RF: **Evaluation of a supermarket intervention to increase consumption of fruits and vegetables**. *American Journal of Health Promotion* 1997, **11**(6):422-425.

23. Winett RA, Anderson ES, Bickley PG, Walberg-Rankin J, Moore JF, Leahy M, Harris CE, Gerkin RE: **Nutrition for a Lifetime System©: A multimedia system for altering food supermarket shoppers' purchases to meet nutritional guidelines**. *Computers in Human Behavior* 1997, **13**(3):371-392.

24. Phipps EJ, Wallace SL, Stites SD, Uplinger N, Brook Singletary S, Hunt L, Axelrod S, Glanz K, Braitman LE: **Using rewards-based incentives to increase purchase of fruit and vegetables in lower-income households: design and start-up of a randomized trial**. *Public Health Nutr* 2013, **16**(5):936-941.

25. Song HJ, Gittelsohn J, Kim M, Suratkar S, Sharma S, Anliker J: **A corner store intervention in a low-income urban community is associated with increased availability and sales of some healthy foods**. *Public Health Nutrition* 2009, **12**(11):2060-2067.

26. Gittelsohn J, Song HJ, Suratkar S, Kumar MB, Henry EG, Sharma S, Mattingly M, Anliker JA: **An urban food store intervention positively affects food-related psychosocial variables and food behaviors**. *Health education & behavior : the official publication of the Society for Public Health Education* 2010, **37**(3):390-402.

27. Ayala GX, Baquero B, Laraia BA, Ji M, Linnan L: **Efficacy of a store-based environmental change intervention compared with a delayed treatment control condition on store customers' intake of fruits and vegetables**. *Public Health Nutr* 2013, **16**(11):1953-1960.

28. Bergen D, Yeh MC: **Effects of energy-content labels and motivational posters on sales of sugar-sweetened beverages: Stimulating sales of diet drinks among adults study**. *Journal of the American Dietetic Association* 2006, **106**(11):1866-1869.

29. Fiske A, Cullen KW: **Effects of promotional materials on vending sales of low-fat items in teachers' lounges**. *Journal of the American Dietetic Association* 2004, **104**(1):90-93.

30. French SA, Jeffery RW, Story M, Breitlow KK, Baxter JS, Hannan P, Snyder MP: **Pricing and promotion effects on low-fat vending snack purchases: The CHIPS study**. *American Journal of Public Health* 2001, **91**(1):112-117.

31. Kocken PL, Eeuwijk J, Kesteren NMCV, Dusseldorp E, Buijs G, Bassa-Dafesh Z, Snel J: **Promoting the Purchase of Low-Calorie Foods From School Vending Machines: A Cluster-Randomized Controlled Study**. *Journal of School Health* 2012, **82**(3):115-122.

32. Huang A, Barzi F, Huxley R, Denyer G, Rohrlach B, Jayne K, Neal B: **The effects on saturated fat purchases of providing internet shoppers with purchase- specific dietary advice: a randomised trial**. *PLoS clinical trials* 2006, **1**(5):e22.
