# Supplementary material for: Nutrition interventions at point-of-sale to encourage healthier food purchasing: a systematic review
Source: BMC Public Health. 2014 Sep 5;14:919. doi: 10.1186/1471-2458-14-919 (PMC4180547; doi:10.1186/1471-2458-14-919)
Supplement: Supplementary file 5 — Additional file 4: Table S4: Targeted products, criteria to identify healthy products, theoretical framework, intervention characteristics, data collection method and reported outcomes, of included studies by intervention type. (DOCX 78 KB) [file 12889_2014_7082_MOESM5_ESM.docx]

**Table 4 –Targeted products, criteria to identify healthy products, theoretical framework, intervention characteristics, data collection method and reported outcomes, of included studies by intervention type**

| **Reference** | **Target products** | | **Criteria to identify healthy foods** | | **Theoretical framework used to inform nutrition education** | | **Intervention intensity** | | **Intervention duration** | | | **Data collection method** | | **Product outcome reported** | | | **Mediator factor reported** | | | |
| --- | --- | --- | --- | --- | --- | --- | --- | --- | --- | --- | --- | --- | --- | --- | --- | --- | --- | --- | --- | --- |
| **Nutrition education alone** | | | | | | | | | | | | | | | | | | | |  |
| **Achabal [**[**1**](#_ENREF_1)**]** | Six produce items: carrots, broccoli, cabbage, cauliflower, kiwifruit, tomatoes | | Fruit and vegetables | | NI^2^ | | Non-interactive | | Short-term (4 weeks) | | | Point-of-sale data and survey | | Vegetable sales data | | | Signage signal | | | |
| **Booth-Butterfield (2004) [**[**2**](#_ENREF_2)**]** | Low-fat milk | | Low-fat milk | | Theory of Reasoned Action | | Non-interactive | | Short-term (6 weeks) | | | Survey | | Low-fat milk sold | | | Belief scores | | | |
| **Connell (2001) [**[**3**](#_ENREF_3)**]** | Fruit and vegetables | | Fruit and vegetables | | Consumer Information Processing model | | Interactive | | Short-term (4 weeks) | | | Survey | | F&V scores | | | Awareness  Knowledge  Beliefs  Attitudes | | | |
| **Ernst 1986 [**[**4**](#_ENREF_4)**]** | Food products low in fat, cholesterol and calories in the diet | |  | | NI | | Non-interactive | | Long-term (48 weeks) | | | Point-of-sale data and survey | | Low-fat milk sold (% of total milk) | | | Knowledge scores | | | |
| **Foster 2014 [**[**5**](#_ENREF_5)**]** | Milk, beverages, ready-to-eat cereals, frozen meals, canned pasta, ice cream, ground meat, salty snacks, frozen pizza, prepacked child lunches, and sliced bread | | Existence of lower calorie items in the category. | | NI | | Interactive | | Short-term (24 weeks) | | | Point-of-sale data | | Sales of target products | | | NA^3^ | | | |
| **Jeffery (1982) [**[**6**](#_ENREF_6)**]** | 25 dairy products (eggs, cottage cheese, yoghurt, milk and cream and frozen desserts) | |  | | NI | | Non-interactive | | Short-term (3 phases: 12 + 6 + 6 weeks over 6 months) | | | Point-of-sale data and survey | |  | | | Knowledge scores | | | |
| **Levy (1985) [**[**7**](#_ENREF_7)**]** | 400 products from over 20 different food categories | |  | | NI | | Non-interactive | | Long-term (2 years) | | | Market share of promoted foods and survey | | Market share | | | Shelf labels use | | | |
| **Milliron (2012) [**[**8**](#_ENREF_8)**]** | “Healthful food” choices (low sodium, calcium rich and immune booster) based on Food and Drug Administration labelling regulations and the American Heart Association | | Food and Drug Administration labelling regulations and the American Heart Association | | NI | | Interactive | | Short-term (4 months) | | | Food receipts and survey | | Total fat (g)/1000 kcal | | | Recall of shelf signs | | | |
| **Ni Mhurchi et al. (2010) ^1^ [**[**9**](#_ENREF_9)**]** | 1032 top selling healthy supermarket foods meeting the Heart Foundation’s Tick program criteria | | Heart Foundation’s Tick program | | NI | | Interactive | | Short-term (24 weeks) | | | Electronic scanner of individual purchase | | Saturated fat to energy (%) | | | NA | | | |
| **Reger (1999) [**[**10**](#_ENREF_10)**]** | Fat-free milk | | Fat-free milk | | NI | | Interactive | | Short-term (6 weeks) | | | Milk point-of-sale data and telephone survey | | Low-fat milk sold (% of total milk) | | | NA | | | |
| **Reger (2000) [**[**11**](#_ENREF_11)**]** | Fat-free milk | | Fat-free milk | | NI | | Interactive | | Short-term (6 -8 weeks) | | | Milk point-of-sale data and telephone survey | | Low-fat milk sold (% of total milk) | | | NA | | | |
| **Rodgers (1994) [**[**12**](#_ENREF_12)**]** | Increasing consumption of F&V and other fibre-containing recommended foods = >-2g fibre and <30% of calories from fat) | | >2g fibre and < 30% of calories from fat/serving | | NI | | Non-interactive | | Long-term (2 years) | | | Point-of-sale data and survey | | Point-of-sale data of eight food categories: dry cereals, baked goods, fresh produce, frozen vegetables, canned vegetables, canned and frozen beans, dried beans, dried fruit, and meat and poultry. | | | Health and diet awareness | | | |
| **Silzer (1994) [**[**13**](#_ENREF_13)**]** | Healthful food items (decreased fat and salt and increased dietary fibre) | |  | | NI | | Interactive | | Short-term (2 hours) | | | Survey | | Dietary scores based on fat and salt | | | Nutrition labelling use | | | |
| **Winett (1991) [**[**14**](#_ENREF_14)**]** | Cruciferous vegetables, fruits, high-fibre cereals, low fat dairy and lean protein sources as well as decreasing consumption of fat from butter, beef and snacks | |  | | Social cognitive theory | | Interactive | | Short-term (6 weeks) | | | Checklist with 230 major food items to collect intended and actual purchase | | Dairy fat, high fat meat, high fibre cereal | | | NA | | | |
| **Winett (1991) brief report* [**[**15**](#_ENREF_15)**]** | Cruciferous vegetables, fruits, high-fibre cereals, low fat dairy and lean protein sources as well as decreasing consumption of fat from butter, beef and snacks | |  | | Social cognitive theory | | Interactive | | Short-term (7 weeks) | | | Checklist with 230 major food items to collect intended and actual purchase | | Low-fat products | | | NA | | | |
| **Nutrition education plus enhanced availability of healthy foods** | | | | | | | | | | | | | | | | | | | |  |
| **Glittelsohn 2010a [**[**16**](#_ENREF_16)**]** | Healthier beverages, healthier snacks (whole grain, lower sugar cereals, low-fat milk, F&V with low-fat dips), healthier condiments (low-fat dressings) and healthier meals (tuna in water) | |  | | Social cognitive theory | | Interactive | | Long-term 4 themed phases, each of 6-8 weeks) | | | 24-h dietary recall | | Healthy eating index | | | Knowledge  awareness | | | |
| **Monetary incentive alone** | | | | | | | | | | | | | | | | | | | |  |
| **Herman [**[**17**](#_ENREF_17)**]** | Fresh fruit and vegetables | Fresh fruit and vegetables | | | NI | | NA | | Short-term (6 months) | | | 24-h dietary recall | | F&V serving / 4186 kJ | | | NA | | | |
| **Ni Mhurchi et al. (2010) ^1 [^**[**^9^**](#_ENREF_9)**^]^** | 1032 top selling healthy supermarket foods meeting the Heart Foundation’s Tick program criteria | Heart Foundation’s Tick program | | | NI | | NA | | Short-term (24 weeks) | | | Electronic scanner of individual purchase | | Saturated fat to energy (%) | | | NA | | | |
| **Sturm (2013) [**[**18**](#_ENREF_18)**]** | Healthy foods including minimally processed fruit and vegetables and non-fat dairy and excluding the less-desirable food group (sweets, chocolates, ice cream, sugary foods, chips, sugar-sweetened beverages, and fried items) | NI | | | NI | | NA | | Long-term (28 months) | | | Point-of-sale data | | Spending on healthy food and on F&V | | | NA | | | |
| **Waterlander (2013) ^3^ [**[**19**](#_ENREF_19)**]** | Fruit and vegetables | NI | | | NI | | NA | | Short-term (6 months) | | | Food receipts and FFQ | | F&V purchase and spending | | | NA | | | |
| **Nutrition education plus monetary incentive** | | | | | | | | | | | | | | | | | | | |  |
| **Aimed at customers** |  | | |  | | | |  | | |  | | | |  | | |  |  |  |
| **Ni Mhurchi et al. (2010) ^1^ [**[**9**](#_ENREF_9)**]** | 1032 top selling healthy supermarket foods meeting the Heart Foundation’s Tick program criteria | | Heart Foundation’s Tick program | | NI | | Interactive | | Short-term (24 weeks) | | | Electronic scanner of individual purchase | | Saturated fat to energy (%) | | | NA | | | |
| **Anderson (1997) [**[**20**](#_ENREF_20)**]** | Cruciferous vegetables, fruits, high-fibre cereals, low fat dairy and lean protein sources as well as decreasing consumption of fat from butter, beef and snacks | |  | | Social cognitive theory | | Interactive | | Short-term (15 weeks) | | | Food receipts | | F&V serving (g), fat (g) and fibre (g) | | | NA | | | |
| **Anderson 2001 [**[**21**](#_ENREF_21)**]** | Cruciferous vegetables, fruits, high-fibre cereals, low fat dairy and lean protein sources as well as decreasing consumption of fat from butter, beef and snacks | |  | | Social cognitive theory | | Interactive | | Short-term (15 weeks) | | | Food receipts, survey and FFQ | | F&V serving / 1000 kcal and fat (%) of energy | | | Self-efficacy, outcomes expectations | | | |
| **Kristal [**[**22**](#_ENREF_22)**]** | Fruit and vegetables | | Fruit and vegetables | | Consumer Information Processing model | | Interactive | | Long-term (12 months) | | | Survey | | F&V purchase and recall use of intervention material | | | Recall use of intervention material and behaviour changes | | | |
| **Winett 1997 [**[**23**](#_ENREF_23)**]** | Cruciferous vegetables, fruits, high-fibre cereals, low fat dairy and lean protein sources as well as decreasing consumption of fat from butter, beef and snacks | |  | | Social cognitive theory | | Interactive | | Short-term (15 weeks) | | | Food receipts and survey | | % calories from fat, fibre (g)/1000kcal and serves of F&V/1000kcal | | | Knowledge | | | |
| **Phipps (2014) [**[**24**](#_ENREF_24)**]** | Fruit and vegetables |  | | | NI | | Interactive | | Short-term (8 weeks) | | | Point-of-sale data and survey | | Serves of F&V | | | NA | | | |
| **Aimed at both store-owners and customers** | | | | | |  | | | |  | | |  | | |  | | |  |  |
| **Song [**[**25**](#_ENREF_25)**]** | Ten healthy foods (based on standard criteria e.g., Low-sugar (<10g/serving) or high fibre cereals (>10% of the daily value per serving)) identified to contribute most energy, fat and sugar intakes, | | <10g sugar/serving and > 10% if fibre/serving | | NI | | Interactive | | Long-term (5x 2-mo themed phases over 10 months) | | | Promoted food sales data and survey | | Stocking score of promoted food | | | Outcomes expectation and self-efficacy scores | | | |
| **Glittelsohn 2010b [**[**26**](#_ENREF_26)**]** | Lower sugar cereals, low-fat milk, F&V with low-fat dips, healthier condiments (low-fat dressings) and Healthier meals (tuna in water) | |  | | Social cognitive theory | | Interactive | | Long-term (5x 2-mo themed phases over 10 months) | | | FFQ | | Intervention exposure scores | | | Knowledge scores, outcomes expectation and self-efficacy scores, and intention | | | |
| **Ayala [**[**27**](#_ENREF_27)**]** | Fruit and vegetables | |  | | NI | | Interactive | | Short-term (8 weeks) | | | Receipts and FFQ | | Purchase and intake of F&V | | | NA | | | |
| **Vending machines** | | | | | | | | | | | | | | | | | | | |  |
| **Bergen 2006 [**[**28**](#_ENREF_28)**]** | Water, diet beverages and sugar-sweetened soft drinks | |  | | NI | | Non-interactive | | Short-term (5 weeks) | | | Point-of-sale data | | The total of all beverages sold | | | NA | | | |
| **Fiske 2004 [**[**29**](#_ENREF_29)**]** | Low-fat snack food items and gum selections | |  | | NI | | Non-interactive | | Short-term (4 weeks) | | | Point-of-sale data | | No. of low-fat items sold and; total machine revenue | | | NA | | | |
| **French 2001 [**[**30**](#_ENREF_30)**]** | Low fat snacks | | < 3g of fat per package | | NI | | Non-interactive | | Short-term (4 weeks) | | | Point-of-sale data | | Proportion of low fat snacks; absolute turnover of low fat snack items; and net profits. | | | NA | | | |
| **Kocken 2012 [**[**31**](#_ENREF_31)**]** | Lower-calorie extra products “favourable” or” moderately unfavourable” | | favourable (<100kcal per item) or moderately unfavourable (100-170 kcal per item) | | NI | | Non-interactive | | Short-term (3x 6- week phases) | | | Point-of-sale data | | Data on stock supplies, orders and sales of all vending machines. | | | NA | | | |
| **Online shopping** | | | | | | | | | | | | | | | | | | | |  |
| **Huang 2006 [**[**32**](#_ENREF_32)**]** | 524 foods including food items commonly purchased containing from 1% to 92% saturated fat and a suitable lower-fat alternate for each | |  | | NI | | Interactive | | Short-term (5 months) | | | Purchase online | | Percentage of saturated fat in 100g of food and cost of the purchased items. | | | NA | | | |

**^1^** Study is mentioned twice because there are two treatments in addition to the control, and each of the treatments fit a different intervention category.

**^2^** NI: No further information available

^3^ NA: Not applicable

1. Achabal DD, McIntyre SH, Bell CH, Tucker N: **The Effect of Nutrition P-O-P Signs on Consumer Attitudes and Behavior**. *Journal of Retailing* 1987, **63**(1):9.

2. Booth-Butterfield S, Reger B: **The message changes belief and the rest is theory: the "1% or less" milk campaign and reasoned action**. *Preventive Medicine* 2004, **39**(3):581-588.

3. Connell D, Goldberg JP, Folta SC: **An intervention to increase fruit and vegetable consumption using audio communications: In-store public service announcements and audiotapes**. *Journal of Health Communication* 2001, **6**(1):31-43.

4. Ernst ND, Wu M, Frommer P, Katz E, Matthews O, Moskowitz J, Pinsky JL, Pohl S, Schreiber GB, Sondik E, Tenney J, Wilbur C, Zifferblatt S: **Nutrition education at the point of purchase: the foods for health project evaluated**. *Prev Med* 1986, **15**(1):60-73.

5. Foster GD, Karpyn A, Wojtanowski AC, Davis E, Weiss S, Brensinger C, Tierney A, Guo W, Brown J, Spross C, Leuchten D, Burns PJ, Glanz K: **Placement and promotion strategies to increase sales of healthier products in supermarkets in low-income, ethnically diverse neighborhoods: a randomized controlled trial**. *The American journal of clinical nutrition* 2014, **99**(6):1359-1368.

6. Jeffery RW, Pirie PL, Rosenthal BS, Gerber WM, Murray DM: **Nutrition education in supermarkets: an unsuccessful attempt to influence knowledge and product sales**. *J Behav Med* 1982, **5**(2):189-200.

7. Levy AS, Matthews O, Stephenson M, Tenney JE, Schucker RE: **The Impact of a Nutrition Information Program on Food Purchases**. *Journal of Public Policy & Marketing* 1985, **4**(1):1-13.

8. Milliron BJ, Woolf K, Appelhans BM: **A point-of-purchase intervention featuring in-person supermarket education affects healthful food purchases**. *J Nutr Educ Behav* 2012, **44**(3):225-232.

9. Ni Mhurchu C, Blakely T, Jiang YN, Eyles HC, Rodgers A: **Effects of price discounts and tailored nutrition education on supermarket purchases: a randomized controlled trial**. *American Journal of Clinical Nutrition* 2010, **91**(3):736-747.

10. Reger B, Wootan MG, Booth-Butterfield S: **Using mass media to promote healthy eating: A community-based demonstration project**. *Preventive Medicine* 1999, **29**(5):414-421.

11. Reger B, Wootan MG, Booth-Butterfield S: **A comparison of different approaches to promote community-wide dietary change**. *American Journal of Preventive Medicine* 2000, **18**(4):271-275.

12. Rodgers AB, Kessler LG, Portnoy B, Potosky AL, Patterson B, Tenney J, Thompson FE, Krebs-Smith SM, Breen N, Mathews O, Kahle LL: **"Eat for Health": A Supermarket Intervention for Nutrition and Cancer Risk Reduction**. *American Journal of Public Health* 1994, **84**(1):72-76.

13. Silzer JS, Sheeshka J, Tomasik HH, Woolcott DM: **AN EVALUATION OF SUPERMARKET SAFARI NUTRITION EDUCATION TOURS**. *Journal of the Canadian Dietetic Association-Revue De L Association Canadienne Des Dietetistes* 1994, **55**(4):179-183.

14. Winett RA, Moore JF, Wagner JL, Hite LA, Leahy M, Neubauer TE, Walberg JL, Walker WB, Lombard D, Geller ES, Mundy LL: **Altering shoppers' supermarket purchases to fit nutritional guidelines: an interactive information system**. *Journal of applied behavior analysis* 1991, **24**(1):95-105.

15. Winett RA, Wagner JL, Moore JF, Walker WB, Hite LA, Leahy M, Neubauer T, Arbour D, Walberg J, Geller ES, Mundy LL, Lombard D: **An experimental evaluation of a prototype public access nutrition information system for supermarkets**. *Health Psychology* 1991, **10**(1):75-78.

16. Gittelsohn J, Vijayadeva V, Davison N, Ramirez V, Cheung LWK, Murphy S, Novotny R: **A Food Store Intervention Trial Improves Caregiver Psychosocial Factors and Children's Dietary Intake in Hawaii**. *Obesity* 2010, **18**:S84-S90.

17. Herman DR, Harrison GG, Afifi AA, Jenks E: **Effect of a targeted subsidy on intake of fruits and vegetables among low-income women in the special supplemental nutrition program for women, infants, and children**. *American Journal of Public Health* 2008, **98**(1):98-105.

18. Sturm R, An R, Segal D, Patel D: **A cash-back rebate program for healthy food purchases in South Africa: results from scanner data**. *Am J Prev Med* 2013, **44**(6):567-572.

19. Waterlander WE, de Boer MR, Schuit AJ, Seidell JC, Steenhuis IH: **Price discounts significantly enhance fruit and vegetable purchases when combined with nutrition education: a randomized controlled supermarket trial**. *The American journal of clinical nutrition* 2013, **97**(4):886-895.

20. Anderson ES, Winett RA, Bickley PG, Walberg-Rankin J, Moore JF, Leahy M, Harris CE, Gerkin RE: **The Effects of a Multimedia System in Supermarkets To Alter Shoppers' Food Purchases: Nutritional Outcomes and Caveats**. *Journal of Health Psychology* 1997, **2**(2):209-223.

21. Anderson ES, Winett RA, Wojcik JR, Winett SG, Bowden T: **A computerized social cognitive intervention for nutrition behavior: Direct and mediated effects on fat, fiber, fruits, and vegetables, self-efficacy, and outcome expectations among food shoppers**. *Annals of Behavioral Medicine* 2001, **23**(2):88-100.

22. Kristal AR, Goldenhar L, Muldoon J, Morton RF: **Evaluation of a supermarket intervention to increase consumption of fruits and vegetables**. *American Journal of Health Promotion* 1997, **11**(6):422-425.

23. Winett RA, Anderson ES, Bickley PG, Walberg-Rankin J, Moore JF, Leahy M, Harris CE, Gerkin RE: **Nutrition for a Lifetime System©: A multimedia system for altering food supermarket shoppers' purchases to meet nutritional guidelines**. *Computers in Human Behavior* 1997, **13**(3):371-392.

24. Phipps EJ, Braitman LE, Stites SD, Singletary SB, Wallace SL, Hunt L, Axelrod S, Glanz K, Uplinger N: **Impact of a Rewards-Based Incentive Program on Promoting Fruit and Vegetable Purchases**. *Am J Public Health* 2014:e1–e7.

25. Song HJ, Gittelsohn J, Kim M, Suratkar S, Sharma S, Anliker J: **A corner store intervention in a low-income urban community is associated with increased availability and sales of some healthy foods**. *Public Health Nutrition* 2009, **12**(11):2060-2067.

26. Gittelsohn J, Song HJ, Suratkar S, Kumar MB, Henry EG, Sharma S, Mattingly M, Anliker JA: **An urban food store intervention positively affects food-related psychosocial variables and food behaviors**. *Health education & behavior : the official publication of the Society for Public Health Education* 2010, **37**(3):390-402.

27. Ayala GX, Baquero B, Laraia BA, Ji M, Linnan L: **Efficacy of a store-based environmental change intervention compared with a delayed treatment control condition on store customers' intake of fruits and vegetables**. *Public Health Nutr* 2013, **16**(11):1953-1960.

28. Bergen D, Yeh MC: **Effects of energy-content labels and motivational posters on sales of sugar-sweetened beverages: Stimulating sales of diet drinks among adults study**. *Journal of the American Dietetic Association* 2006, **106**(11):1866-1869.

29. Fiske A, Cullen KW: **Effects of promotional materials on vending sales of low-fat items in teachers' lounges**. *Journal of the American Dietetic Association* 2004, **104**(1):90-93.

30. French SA, Jeffery RW, Story M, Breitlow KK, Baxter JS, Hannan P, Snyder MP: **Pricing and promotion effects on low-fat vending snack purchases: The CHIPS study**. *American Journal of Public Health* 2001, **91**(1):112-117.

31. Kocken PL, Eeuwijk J, Kesteren NMCV, Dusseldorp E, Buijs G, Bassa-Dafesh Z, Snel J: **Promoting the Purchase of Low-Calorie Foods From School Vending Machines: A Cluster-Randomized Controlled Study**. *Journal of School Health* 2012, **82**(3):115-122.

32. Huang A, Barzi F, Huxley R, Denyer G, Rohrlach B, Jayne K, Neal B: **The effects on saturated fat purchases of providing internet shoppers with purchase- specific dietary advice: a randomised trial**. *PLoS clinical trials* 2006, **1**(5):e22.
